# A draft genome sequence of the elusive giant squid, *Architeuthis dux*

**DOI:** 10.1093/gigascience/giz152

**Published:** 2020-01-16

**Authors:** Rute R da Fonseca, Alvarina Couto, Andre M Machado, Brona Brejova, Carolin B Albertin, Filipe Silva, Paul Gardner, Tobias Baril, Alex Hayward, Alexandre Campos, Ângela M Ribeiro, Inigo Barrio-Hernandez, Henk-Jan Hoving, Ricardo Tafur-Jimenez, Chong Chu, Barbara Frazão, Bent Petersen, Fernando Peñaloza, Francesco Musacchia, Graham C Alexander, Hugo Osório, Inger Winkelmann, Oleg Simakov, Simon Rasmussen, M Ziaur Rahman, Davide Pisani, Jakob Vinther, Erich Jarvis, Guojie Zhang, Jan M Strugnell, L Filipe C Castro, Olivier Fedrigo, Mateus Patricio, Qiye Li, Sara Rocha, Agostinho Antunes, Yufeng Wu, Bin Ma, Remo Sanges, Tomas Vinar, Blagoy Blagoev, Thomas Sicheritz-Ponten, Rasmus Nielsen, M Thomas P Gilbert

**Affiliations:** 1 Center for Macroecology, Evolution and Climate (CMEC), GLOBE Institute, University of Copenhagen, Universitetsparken 15, 2100 Copenhagen, Denmark; 2 The Bioinformatics Centre, Department of Biology, University of Copenhagen, Ole Maaløes Vej 5, 2200 Copenhagen, Denmark; 3 Department of Biochemistry, Genetics and Immunology, University of Vigo, Vigo 36310, Spain; 4 CIIMAR, Interdisciplinary Centre of Marine and Environmental Research, University of Porto, Terminal de Cruzeiros de Leixões, Av. General Norton de Matos, 4450'208 Matosinhos, Portugal; 5 Faculty of Mathematics, Physics and Informatics, Comenius University in Bratislava, Mlynská dolina, 842 48 Bratislava, Slovak Republic; 6 Eugene Bell Center for Regenerative Biology and Tissue Engineering, Marine Biological Laboratory, Woods Hole, MA 02543, USA; 7 Department of Biochemistry, University of Otago, 710 Cumberland Street, North Dunedin, Dunedin 9016, New Zealand; 8 Centre for Ecology and Conservation, University of Exeter, Penryn Campus, Penryn, Cornwall, TR10 9FE, UK; 9 Department of Biochemistry and Molecular Biology, University of Southern Denmark, Campusvej 55, 5230 Odense M, Denmark; 10 GEOMAR Helmholtz Centre for Ocean Research Kiel,Wischhofstraße 1-3, 24148 Kiel, Germany; 11 Instituto del Mar del Perú, Esq. Gamarra y Gral. Valle, Chucuito Apartado 22, Callao, Peru; 12 Department of Biomedical Informatics, Harvard Medical School, Boston, MA, USA; 13 IPMA, Fitoplâncton Lab, Rua C do Aeroporto, 1749-077, Lisboa, Portugal; 14 Centre of Excellence for Omics-Driven Computational Biodiscovery (COMBio), Faculty of Applied Sciences, AIMST University, Batu 3 1/2, Butik Air Nasi, 08100 Bedong, Kedah, Malaysia; 15 Evolutionary Genomics Section, Globe Institute, University of Copenhagen,Øster Farimagsgade 5, 1353 Copenhagen, Denmark; 16 Unidad de Investigación Biomédica en Cáncer, Instituto Nacional de Cancerología-Instituto de Investigaciones Biomédicas, Universidad Nacional Autónoma de México, Mexico City, México; 17 Genomic Medicine, Telethon Institute of Genetics and Medicine, Via Campi Flegrei, 34, 80078 Pozzuoli, Naples, Italy; 18 GCB Sequencing and Genomic Technologies Shared Resource, Duke University CIEMAS, 101 Science Drive, Durham, NC 27708, USA; 19 i3S-Instituto de Investigação e Inovação em Saúde, Universidade do Porto, Rua Alfredo Allen 208, 4200-135 Porto, Portugal; 20 IPATIMUP-Institute of Molecular Pathology and Immunology, University of Porto, Rua Júlio Amaral de Carvalho 45, 4200-135 Porto, Portugal; 21 Faculty of Medicine of the University of Porto, Alameda Prof. Hernani Monteiro, 4200-319 Porto, Portugal; 22 Section for GeoGenetics, GLOBE Institute, University of Copenhagen, Øster Voldgade 5-7, 1350 Copenhagen, Denmark; 23 Department of Molecular Evolution and Development, University of Vienna, Althanstrasse 14 (UZA1), A-1090 Vienna, Austria; 24 Novo Nordisk Foundation Center for Protein Research, Faculty of Health and Medical Sciences, University of Copenhagen, Blegdamsvej 3B, 2200 Copenhagen, Denmark; 25 Bioinformatics Solutions Inc, 470 Weber St N Suite 204, Waterloo, ON N2L 6J2, Canada; 26 School of Biological Sciences and School of Earth Sciences, University of Bristol, 24 Tyndall Avenue, Bristol, BS8 1TG, UK; 27 Howard Hughes Medical Institute, 4000 Jones Bridge Rd, Chevy Chase, MD 20815, USA; 28 The Rockefeller University, 1230 York Ave, New York, NY 10065, USA; 29 European Molecular Biology Laboratory, European Bioinformatics Institute (EMBL-EBI), Wellcome Genome Campus, Hinxton, Cambridge CB10 1SD, UK; 30 Section for Ecology and Evolution, Department of Biology, University of Copenhagen, Universitetsparken 15, 2100 Copenhagen, Denmark; 31 China National Genebank, BGI-Shenzhen, Shenzhen 518083, China; 32 State Key Laboratory of Genetic Resources and Evolution, Kunming Institute of Zoology, Chinese Academy of Sciences, 32 Jiaochang Donglu Kunming, Yunnan 650223, China; 33 CAS Center for Excellence in Animal Evolution and Genetics, Chinese Academy of Sciences, 32 Jiaochang Donglu Kunming, Yunnan 650223, China; 34 Centre for Sustainable Tropical Fisheries & Aquaculture, James Cook University, Townsville, Douglas QLD 4814, Australia; 35 Department of Ecology, Environment and Evolution, School of Life Sciences, La Trobe University, Melbourne Victoria 3086, Australia; 36 Department of Biology, Faculty of Sciences, University of Porto, Rua do Campo Alegre, 4169-007, Porto, Portugal; 37 BGI-Shenzhen, Shenzhen, China; 38 Biomedical Research Center (CINBIO), University of Vigo, Campus Universitario Lagoas-Marcosende, 36310 Vigo, Spain; 39 Department of Computer Science and Engineering, University of Connecticut, Storrs, CT 06269, USA; 40 School of Computer Science, University of Waterloo, 200 University Ave W, Waterloo, ON N2L 3G1, Canada; 41 Area of Neuroscience, Scuola Internazionale Superiore di Studi Avanzati (SISSA), Via Bonomea 265, 34136 Trieste, Italy; 42 Biology and Evolution of Marine Organisms, Stazione Zoologica Anton Dohrn, Villa Comunale, 80121 Napoli, Italy; 43 Departments of Integrative Biology and Statistics, University of California, 3040 Valley Life Sciences, Berkeley, CA 94720-3200, USA; 44 Norwegian University of Science and Technology, University Museum, Høgskolering 1, 7491 Trondheim, Norway

**Keywords:** cephalopod, invertebrate, genome assembly

## Abstract

**Background:**

The giant squid (*Architeuthis dux*; Steenstrup, 1857) is an enigmatic giant mollusc with a circumglobal distribution in the deep ocean, except in the high Arctic and Antarctic waters. The elusiveness of the species makes it difficult to study. Thus, having a genome assembled for this deep-sea–dwelling species will allow several pending evolutionary questions to be unlocked.

**Findings:**

We present a draft genome assembly that includes 200 Gb of Illumina reads, 4 Gb of Moleculo synthetic long reads, and 108 Gb of Chicago libraries, with a final size matching the estimated genome size of 2.7 Gb, and a scaffold N50 of 4.8 Mb. We also present an alternative assembly including 27 Gb raw reads generated using the Pacific Biosciences platform. In addition, we sequenced the proteome of the same individual and RNA from 3 different tissue types from 3 other species of squid (*Onychoteuthis banksii, Dosidicus gigas*, and *Sthenoteuthis oualaniensis*) to assist genome annotation. We annotated 33,406 protein-coding genes supported by evidence, and the genome completeness estimated by BUSCO reached 92%. Repetitive regions cover 49.17% of the genome.

**Conclusions:**

This annotated draft genome of *A. dux* provides a critical resource to investigate the unique traits of this species, including its gigantism and key adaptations to deep-sea environments.

## Context

Cephalopods are the most behaviourally complex of the invertebrate protostomes [1]. Their large, highly differentiated brains are comparable in relative size and complexity to those of vertebrates [2], as are their cognitive capabilities [1]. Cephalopods are distributed worldwide from tropical to polar marine habitats, from benthic to pelagic zones, and from intertidal areas down to the abyssal parts of the deep sea, with the only exception being the Black Sea. Cephalopod populations are thought to be currently increasing in some regions for a variety of reasons [3], including potential predator release as a consequence of the depletion of fish stocks [4]. The class Cephalopoda contains ∼800 species, with the vast majority belonging to the soft-bodied subclass Coleoidea (cuttlefishes, octopuses, and squids), and a small handful belonging to the Nautiloidea (nautiluses) [5]. Cephalopods are ecologically important as a primary food source for marine mammals, birds, and for many fish species. They are also increasingly important as a high-protein food source for humans and are a growing target for commercial fisheries and farming [6].

Cephalopods show a wide variety of morphologies, lifestyles, and behaviours [7], but with the exception of the nautiluses they are characterized by rapid growth and short lifespans, despite a considerable investment in costly sensory adaptations [2]. They range in size from the tiny pygmy squids (∼2 cm) to animals that are nearly 3 orders of magnitude larger, such as the giant squid, *A. du*x (average length 10–12 m, and reported up to 20 m total length) [6, 8, 9], to the colossal squid, *Mesonychoteuthis hamiltoni* (maximum length remains unclear, but a recorded weight of 500 kg makes it the largest known invertebrate [10]). Cephalopods can rapidly alter the texture, pattern, colour, and brightness of their skin, and this both enables a complex communication system, as well as provides exceptional camouflage and mimicry [11]. Together these allow cephalopods to both avoid predators, and hunt prey highly efficiently, making them some of the top predators in the ocean. The remarkable adaptations of cephalopods also extend to their genome, with recent work demonstrating increased levels of RNA editing to diversify proteins involved in neural functions [12].

Over recent years, oceanic warming and acidification, pollution, expanding hypoxia, and fishing [13–15] have been shown to affect cephalopod populations. Mercury has been found in high concentrations in the tissue of giant squid specimens [16], and accumulation of flame retardant chemicals has also been detected in the tissue of deep-sea cephalopods [17]. Consequently, there is an urgent need for greater biological understanding of these important, but rarely encountered animals, in order to aid conservation efforts and ensure their continued existence. A genome is an important resource for future population genomics studies aiming at characterizing the diversity of the legendary giant squid, the species which has inspired generations to tell tales of the fabled Kraken.

## Methods

### DNA extraction, library building, and *de novo* genome assembly

High-molecular-weight genomic DNA was extracted from a single *A. du*x individual (NCBI:txid256136; marinespecies.org:taxname:342218) using a cetyl trimethylammonium bromide–based buffer followed by organic solvent purification, following Winkelmann et al. [18] (details in the Supplementary Methods). We generated 116 Gb of raw reads from Illumina short-insert libraries, 76 Gb of paired-end reads from libraries ranging from 500 to 800 bp in insert size, and 5.4 Gb of mate-pair with a 5-kb insert (Supplementary Table S1). Furthermore, we generated 3.7 Gb of paired-end reads using Moleculo libraries (3 high-throughput libraries and 4 high-fidelity libraries). The *k*-mer distribution of the reads under a diploid model in kmergenie [19] predicted the genome size to be 2.7 Gb.

An initial assembly generated with Meraculous (Meraculous, RRID:SCR_010700) [20] using Illumina and Moleculo data (N50 of 32 kb, assembly statistics in Supplementary Table S2) was used as input for Dovetail Genomic's HiRise scaffolding software together with the Hi-C data generated from 2 Chicago libraries corresponding to a physical coverage of the genome of 52.1×. This “Meraculous + Dovetail” assembly (statistics in Table 1) was the one used for the genome annotation (non-coding RNAs, protein-coding genes, and repeats) and comparative genomics analyses presented in this article. Further scaffolding was performed using 23.38 Gb of Pacific Biosciences reads (19 single-molecule real-time sequencing [SMRT] cells, mean read length of 14.79 kb) using the default parameters in PBJelly (PBJelly, RRID:SCR_012091) [21] (see assembly statistics in Supplementary Table S2). The genome gene content completeness was evaluated through the BUSCO (BUSCO, RRID:SCR_015008) v.3.0.2 datasets: Eukaryota, Metazoan [22].

**Table 1:** Statistics of the giant squid genome assembly (Meraculous + Dovetail) and corresponding gene prediction and functional annotation

Global statistics	Genome	Gene models with evidence
Genome assembly*		
Input assembly	Meraculous	
Contig N50 length (Mb)	0.005	
Longest contig (Mb)	0.120	
Scaffold N50 length (Mb)	4.852	
Longest scaffold (Mb)	32.889	
Total length (Gb)	2.693	
BUSCO statistics (^1^Euk/^2^Met) (%)		
Complete BUSCOs	86.1/88.5	81.6/78.3
Complete and single-copy	85.1/87.6	79.9/77.7
Complete and duplicated	1.0/0.9	1.7/0.6
Partial	4.3/3.6	9.6/5.7
Missing	9.6/7.9	8.8/16.0
Total BUSCOs found	90.4/92.1	91.2/84.0
Genome annotation/gene prediction		
Protein-coding gene number	33,406	
Transcript evidence	30,472	
Mean protein length (aa)	339	
Longest protein (aa)	17,047	
Mean CDS length (bp)	1,015	
Longest CDS (bp)	51,138	
Mean exon length (bp)	199	
Mean exons per gene	5	
Functional annotation (number of hits)		
Swissprot	15,749	
Uniref90	29,553	
Gene Ontology terms	4,712	
Conserved Domains Database	15,280	

The transcript evidence was confirmed by blastp hits with e-value < 10E^−6^ using the transcriptomes of 3 other species of squid (see the “Transcriptome sequencing” section).

*The presented statistics are to contigs/scaffolds with length ≥500 bp.

1 Euk: Database of Eukaryota orthologs genes, containing a total of 303 BUSCO groups.

2 Met: Database of Metazoa orthologs genes, containing a total of 978 BUSCO groups.

### Transcriptome sequencing and de novo assembly

Given the extreme rarity of live giant squid sightings, we were unable to collect fresh organ samples (following the recommendations of Moltschaniwskyj et al. [23]) containing intact RNA from the species to assist with the genome annotation. As an alternative, we extracted total RNA from gonad, liver, and brain tissue from live-caught specimens of 3 other oegopsid squid species (*Onychoteuthis banksii*, NCBI:txid392296;*Dosidicus gigas*, NCBI:txid346249; and *Sthenoteuthis oualaniensis*, NCBI:txid34553; Supplementary Fig. S1), using the Qiagen RNeasy extraction kit (Qiagen, ValenciaCA, USA). The RNA integrity and quantity were measured on a Qubit fluorometer (Invitrogen, Eugene, OR, USA) and on the Agilent Bioanalyzer 2100 (Agilent, Santa Clara, CA, USA). The Illumina TruSeq Kit v.2.0 was used to isolate the messenger RNA and prepare complementary DNA libraries for sequencing, following the recommended protocol. Compatible index sequences were assigned to individual libraries to allow for multiplexing on 4 lanes of 100-bp paired-end technology on an Illumina HiSeq 2000 flow cell. Sequencing of the complementary DNA libraries was performed at the National High-Throughput Sequencing Center at the University of Copenhagen in Denmark. We assessed the quality of the raw reads using FastQC (FastQC, RRID:SCR_014583) v0.10.0 [24]. After removing indexes and adaptors with CutAdapt [25], we trimmed the reads with the FASTX-toolkit [26], removing bases with a Phred-scale quality score <25. Reference transcriptomes for each species were built after pooling the reads from all tissues and using these as input in Trinity (Trinity, RRID:SCR_013048) [27]. This software was used with the default settings including a fixed *k*-mer size of 25 as suggested by the authors. Annotation of coding regions was performed with the EvidentialGene pipeline [28].

### Protein extraction, separation by 1D SDS-PAGE, MALDI-TOF/TOF, and protein identification

Given the practical impossibility of obtaining RNA from a giant squid specimen, we produced a library of giant squid peptide sequences to guide the gene annotation process.

Proteins were solubilized from a giant squid mantle tissue sample according to the procedure described by Kleffmann et al. [29] and using the following buffers: (i) 40 mM Tris–HCl, 5 mM MgCl_2_, and 1 mM dithiothreitol (DTT), pH 8.5; (ii) 8 M urea, 20 mM Tris, 5 mM MgCl_2_, and 20 mM DTT; (iii) 7 M urea, 2 M thiourea, 20 mM Tris, 40 mM DTT, 2% CHAPS (w/v), and 1% Triton X-100 (v/v), and (iv) 40 mM Tris, 4% sodium dodecyl sulfate (SDS) (w/v), and 40 mm DTT. All buffers were augmented with protease inhibitors (Halt™ Protease Inhibitor Cocktail, EDTA-Free, Thermo Scientific). Tissue samples were ground in liquid nitrogen before homogenization, or homogenized directly with ultrasound (probe sonication at 60 Hz, for 3 min) in buffer (i). Solubilized proteins were collected by ultracentrifugation at 100,000*g* and 4°C. Each extraction was performed in duplicate for each specific buffer and extracts were pooled. Protein extracts were subsequently stored at −20°C. Total protein content was estimated according to the Bradford method [30].

Protein separation by 1D SDS-PAGE (polyacrylamide gel electrophoresis) was carried out as described in Santos et al. [31]. A total of 53 µL of sample (39 µg protein) was diluted in 72 µL of loading buffer (0.01% bromophenol blue, 2% SDS, 20% glycerol, 5% β-mercaptoethanol [w/v/v] in 62.5 mM Tris-HCl, pH 6.8). The resulting solution was heated for 3 min at 99°C. Proteins were separated by SDS-PAGE with 12% (w/v) polyacrylamide gels. Electrophoresis was carried out using the mini Protean Cell (BioRad) at a constant voltage of 150 V. The separated proteins were visualized by staining with colloidal Coomassie brilliant blue [32], and lanes were cut into 15 gel sections for subsequent LC-MS/MS analysis.

### LC-MS/MS analyses

All samples were analysed with the Easy-nLC system (Thermo Fisher Scientific), connected online to a Q Exactive mass spectrometer (Thermo Fisher Scientific) equipped with a nanoelectrospray ion source (Thermo Fisher Scientific). Tryptic peptides were loaded in a fused silica column (75  µm inner diameter) packed with C18 resin (3-µm beads, Reprosil, Dr. Maisch), with solvent A (0.5% acetic acid). They were then eluted with a 120-minute gradient of solvent B (80% acetonitrile, 0.5% acetic acid) with a constant flow of 250 nL/min. The Q exactive was operated in positive mode with a capillary temperature of 250 °C, using the data-dependent acquisition method, which switches from full MS scans to MS/MS scans for the 12 most intense ions. Fragmentation was achieved by higher-energy collisional dissociation with a normalized collisional energy of 25. Full MS ranged from 300 to 1,750 *m/z* at a resolution of 70,000, an automatic gain control of 1e6, and a maximum injection time of 120 ms, whereas MS/MS events were scanned at a resolution of 35,000, an automatic gain control of 1e5, maximum injection time of 124 ms, isolation windows of 2 *m/z*, and an exclusion window of 45 seconds.

### *de novo* peptide prediction

Raw LC-MS/MS data were read using Thermo Fisher MSRawFileReader 2.2 library and imported into PEAKS Studio 7.0 and subsequently pre-processed for precursor mass and charge correction, MS/MS de-isotoping, and deconvolution. PEAKS *de novo* sequencing [32] was performed on each refined MS/MS spectrum with a precursor and fragment ion error tolerance of 7 ppm and 0.02 Da, respectively. Carbamidomethylation (Cys) was set as a fixed modification, and oxidation (Met) and N-terminal Acetylation as variable modifications. At most, 5 variable modifications per peptide were allowed. For each tandem spectrum, 5 *de novo* candidates were reported along with their local confidence scores (the likelihood of each amino acid assignment in a *de novo* candidate peptide). This score was used to determine the accuracy of the *de novo* peptide sequences. The top *de novo* peptide for each spectrum was determined by the highest average local confidence score among the candidates for that spectrum.

### Genome annotation

Protein-coding genes were predicted by ExonHunter [33], which combines probabilistic models of sequence features with external evidence from alignments. As external evidence, we have used the transcriptomes of oegopsid squid species obtained as a part of this project (*O. banksii, D. gigas*, and *S. oualaniensis*); these transcripts were translated into proteins to facilitate cross-species comparison. In addition, known proteins from *(Octopus bimaculoides*), *Crassostrea gigas* (Pacific oyster), and *Lottia gigantea* (Giant owl limpet) were used to inform the gene prediction process. The proteins were aligned to the genome by BLASTX. *De novo* identified MS/MS-based peptides were initially also considered as external evidence but were later omitted owing to low coverage. Evidence from predicted repeat locations was used to discourage the model from predicting genes overlapping repeats. Because no sufficiently close annotated genome was available for training gene-finding parameters, ExonHunter was first run using *Drosophila melanogaster* parameters on a randomly chosen subset of 118 scaffolds longer than 200 kb (total length 199 Mb). Out of 12,912 exons predicted in this run, 5,716 were supported by protein alignment data and selected to train the parameters of the gene-finding model for *A. dux*, using the methods described by Brejová et al. [33]. Such iterative training has been previously shown to yield gene prediction results similar to those of training on curated gene sets [33–35]. Rerunning ExonHunter with the resulting *A. dux* model parameters on the entire genome yielded 51,225 candidate gene prediction genes. Gene prediction in *A. dux* is challenging owing to the fragmentary nature of the genome assembly (60% of predictions span a sequencing gap). This results in a significant number of artifacts, e.g., short genes with long introns spanning gaps in the assembly. A total of 18,054 predictions yield protein product shorter than 100 amino acids (aa), yet the median span of these predictions is >4 kb and only 32% of them are supported by transcript or protein alignments. In contrast, 83% of genes with product longer than 100 aa are supported. Another factor contributing negatively to gene prediction quality is the lack of RNA-sequencing data from *A. dux* due to unavailability of fresh organ samples. In most of the analyses below, we consider only 33,406 genes that were found to have transcript evidence (blastp match to a sequence from a cephalopod transcriptome, with ≥50% of the giant squid coding region covered) and/or matches in Swissprot or UniRef90 databases (Table 1). This supported set contains much fewer extremely short genes (Supplementary Fig. S4).

The function of the protein-coding genes was inferred with Annocript 0.2 [36], which is based on the results from blastp [37] runs against SwissProt and UniRef90. In addition, we performed a rpsblast search using matrices from the Conserved Domain Database to annotate specific domains present on the protein queries.

Non-coding RNAs were annotated using the cmsearch program from INFERNAL 1.1 (INFERNAL, RRID:SCR_011809) and the covariance models (CMs) from the Rfam database v12.0 [38, 39]. All matches above the curated GA threshold were included. INFERNAL was selected because it implements the CMs that provide the most accurate bioinformatic annotation tool for non-coding RNAs (ncRNAs) available [40]. tRNA-scan v.1.3.1 was subsequently used to refine the annotation of transfer RNA (tRNA) genes (Supplementary Table S3). The method uses a number of heuristics to increase the search speed, annotates the isoacceptor type of each prediction, and infers whether predictions are likely to be functional or tRNA-derived pseudogenes [41, 42]. This method uses CMs to identify tRNAs. Rfam matches and the tRNA-scan results for families belonging to the same clan were then “competed,” so that only the best match was retained for any genomic region [39].

### Transposable element annotation

Repetitive elements were first identified using RepeatMasker (RepeatMasker, RRID:SCR_012954) v.4.0.8 [43] with the eukaryota RepBase [44] repeat library. Low-complexity repeats were ignored (-nolow) and a sensitive (-s) search was performed. Following this, a *de novo* repeat library was constructed using RepeatModeler (RepeatModeler, RRID:SCR_015027) v.1.0.11 [45], including RECON v.1.08 [46] and RepeatScout (RepeatScout, RRID:SCR_014653) v.1.0.5 [47]. Novel repeats identified by RepeatModeler were analyzed with a “BLAST, Extract, Extend” process to characterize elements along their entire length [48]. Consensus sequences and classification information for each repeat family were generated. The resulting *denovo* repeat library was used to identify repetitive elements using RepeatMasker.

## Data Analyses

We present a main draft genome assembly produced using 200 Gb of Illumina reads, 4 Gb of Moleculo synthetic long reads, and 108 Gb of Chicago libraries, with a final size matching the estimated genome size of 2.7 Gb, and a scaffold N50 of 4.8 Mb (assembly and annotation statistics in Table 1). Genome completeness estimated by BUSCO reached 90.4% (Eukaryota) and 92.1% (Metazoa), and the completeness for the 33,406 protein-coding genes was 91.2% (Eukaryota) and 84.0% (Metazoa).

We also produced an alternative assembly including 27 Gb raw reads generated using the Pacific Biosciences platform, but this showed minimal improvement in assembly statistics, genome size larger than predicted, and lower BUSCO completeness (Supplementary Table S2).

### Comparative analyses of transposable elements

**Figure 1:**
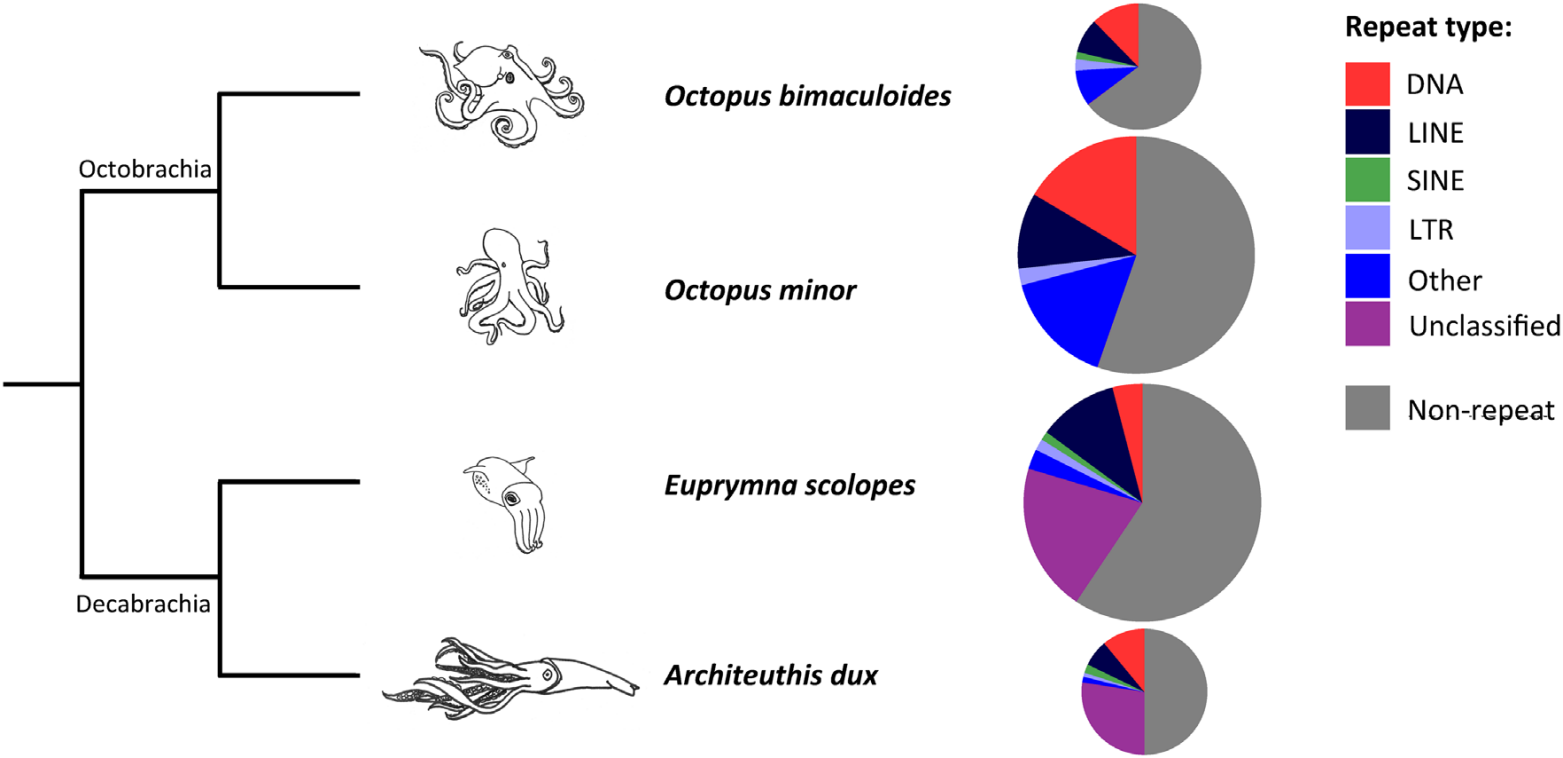
Comparison of genome repeat content among available cephalopod genomes with assembled genomes (repeat data for *O. minor* and *O. bimaculoides* from [52] and for *E. scolopes* from [53]). The tree indicates evolutionary relationships among the 2 available octopod cephalopods and the 2 available decapod cephalopods. Pie charts are scaled according to genome size (*O. bimaculoides*: 2.7 Gb, *O. minor*: 5.09 Gb, *E. scolopes*: 5.1 Gb, *A. dux*: 2.7 Gb), with repeat types indicated by colour.

We estimated the total repeat content of the giant squid genome to be approximately half its total size (∼49.1%) (Fig. 1, Supplementary Table S4). Of all the repeats present in the giant squid genome, only a few were predicted to be small RNAs, satellites, or simple or low-complexity repeats (∼0.89% of the total genome), with the vast majority (∼48.21%) instead consisting of transposable elements (TEs; i.e., short interspersed nuclear elements [SINEs], long interspersed nuclear elements [LINEs], long terminal repeat [LTR] retrotransposons, and DNA transposons; Fig. 1, Supplementary Table S4). Of the TE portion of the giant squid genome, the main contribution from annotated TEs is from DNA elements (11.06%) and LINEs (6.96%), with only a small contribution from SINEs (1.99%) and LTR elements (0.72%). TEs are a nearly universal feature of eukaryotic genomes, often comprising a large proportion of the total genomic DNA (e.g., the maize genome is ∼85% TEs [49], stick insect genome is ∼52% TEs [50], and the human genome is >45% TEs [51]); consequently, these account for the majority of observed genome size variation among animals.

In Fig. 1, we summarize the recently reported TE analyses performed on assembled cephalopod genomes, as follows: California two-spot octopus (*Octopus bimaculoides*) [11] and long-arm octopus (*Octopus minor*) [52], Hawaiian bobtail squid (*Euprymna scolopes*) [53], and giant squid (*A. dux*). The varying sequencing strategies used to generate currently available cephalopod genomes (and accompanying variation in assembly quality) complicate the comparative analysis of TE content for this group. However, notwithstanding this caveat, it does seem clear that TEs make up a large fraction of the total genomic content across all cephalopod genomes published to date (Fig. 1). DNA transposons and LINEs dominate in available cephalopod genomes, while LTR elements and SINEs generally represent a minor portion of cephalopod TEs (Fig. 1). Within decapod cephalopods (i.e., squid and cuttlefish), patterns in TE content are generally similar; however, the giant squid has a notably larger proportion of DNA transposons (1,626,482 elements, 11.06% of the total genome) than the Hawaiian bobtail squid (855,308 elements, 4.05% of the total genome), with the bobtail squid in turn having a similar proportion of LINEs (752,629 elements, 6.83% of the total genome) to the giant squid (766,382 elements, 6.96% of the total genome; Fig. 1).

**Figure 2:**
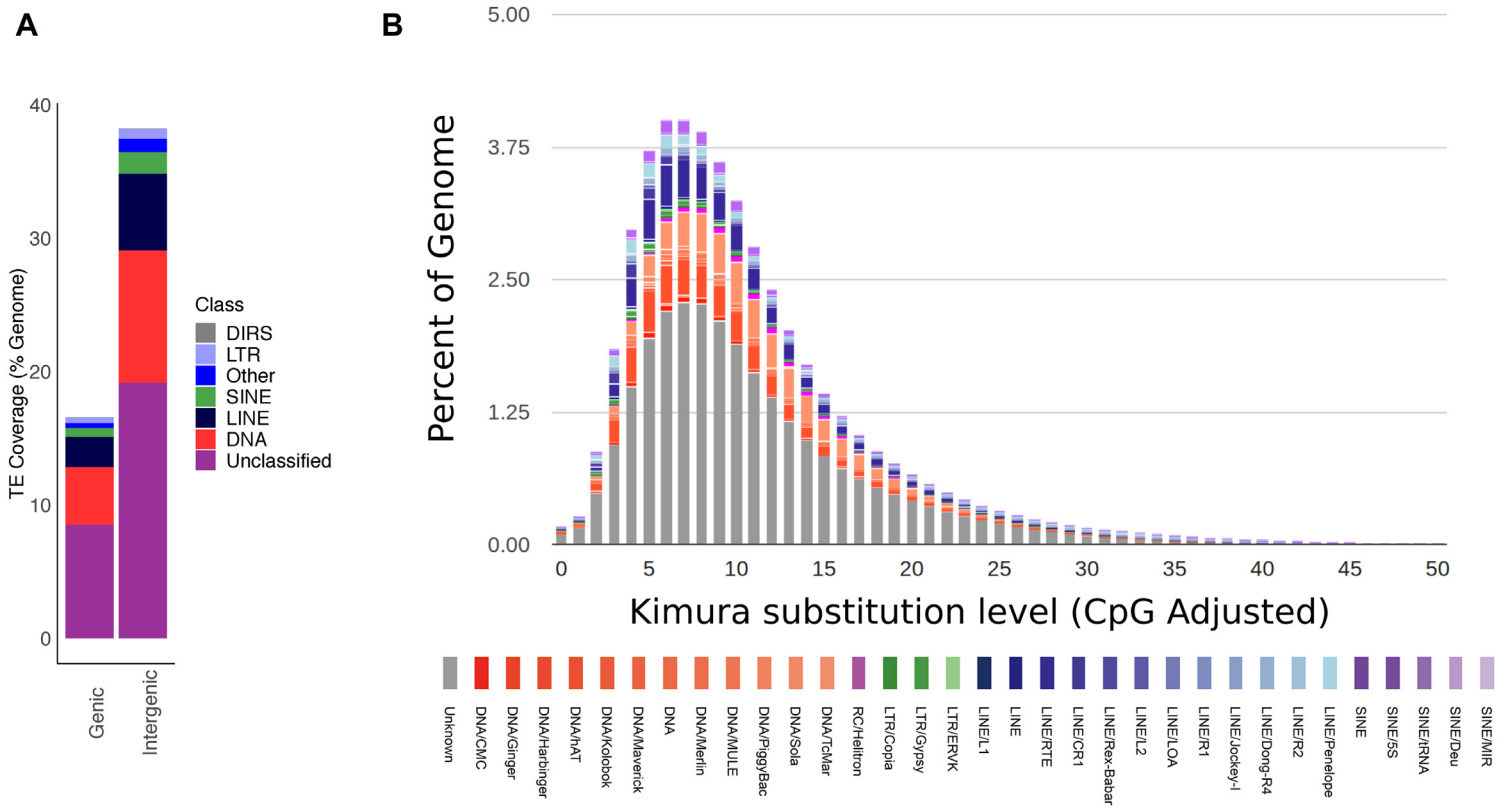
**(A)** Stacked bar chart illustrating the proportions (expressed as percentage of the total genome) of repeats found in genic (≤2 kb from an annotated gene) and intergenic regions (>2 kb from an annotated gene) for the giant squid genome. TE classes include DIRS: *Dictyostelium* intermediate repeat sequence 1 - like elements**. (B)** Transposable element (TE) accumulation history in the giant squid genome, based on a Kimura distance-based copy divergence analysis of TEs, with Kimura substitution level (CpG adjusted) illustrated on the x-axis, and percentage of the genome represented by each repeat type on the y-axis. Repeat type is indicated by bar colour.

The defining ability of TEs to mobilize, in other words, to transfer copies of themselves into other parts of the genome, can result in harmful mutations. However, TEs can also facilitate the generation of genomic novelty, and there is increasing evidence of their importance for the evolution of host-adaptive processes [54]. In the giant squid genome, all classes of TEs were more frequent (∼38.23%) in intergenic regions (here defined as regions >2 kb upstream or downstream of an annotated gene) than in genic regions versus percentage of the genome in intergenic regions (∼16.6%; Fig. 2A). These findings are broadly similar to those reported for other cephalopods, although a larger proportion of the giant squid genome is composed of repeats located within genic regions (percentage of the genome represented by TEs for *Octopus bimaculoides*: ∼6% genic versus ∼30% intergenic, and for *O. minor*: ∼6% genic versus ∼40% intergenic [52]).

A Kimura distance-based copy divergence analysis revealed that the most frequent TE sequence divergence relative to the TE consensus sequence in the giant squid genome was ∼5–8% across all repeat classes, suggesting a relatively recent transposition burst across all major TE types (Fig. 2B). Divergence peaks were most pronounced in LINE RNA transport elements, Tc/Mar and hAT DNA transposons, and unclassified TEs, with smaller divergence peaks in SINE tRNA elements and Penelope LINE elements (Fig. 2B). Divergence peaks were most pronounced in LINE RNA transport elements, Tc/Mar and hAT DNA transposons, and unclassified TEs, with smaller divergence peaks in SINE tRNA elements and Penelope LINE elements (Fig. 2B). In comparison to observations from other cephalopods, these results suggest a shorter and more intense burst of recent TE activity in the giant squid genome. Overall, further genomic sampling within each of the cephalopod clades will be needed to understand TE evolution, as closely related species can show significant differences (e.g., *O. bimaculoides* to *O. vulgaris*) [55].

### Non-coding RNAs

We identified 50,598 ncRNA-associated loci in the squid sequencing data, using curated homology-based probabilistic models from the Rfam database [56] and the specialized tRNAscan-SE (tRNAscan-SE, RRID:SCR_010835) tRNA annotation tool [41]. The essential and well-conserved Metazoan ncRNAs: tRNAs, ribosomal RNAs (rRNAs) (5S, 5.8S, SSU, and LSU), RNase P, RNase MRP, SRP, and the major spliceosomal snRNAs (U1, U2, U4, U5, U6), as well as the minor spliceosomal snRNAs (U11, U12, U4atac, and U6atac), are all found in the *A. dux* genome. Some of the copy numbers associated with the core ncRNAs are extreme. For example, we identified (i) ∼24,000 loci that seem to derive from 5S rRNA, (ii) ∼17,000 loci that are predicted to be tRNA derived, (iii) ∼3,200 valine tRNAs isotypes and ∼1,300 U2 spliceosomal RNAs. The microRNA mir-598 also exhibits high copy numbers at 172. Many of these are likely to be SINEs derived by transposition. All 20 tRNA isotypes were identified in the *A. dux* genome. Again, many of these had relatively large copy numbers (summarized in Table 1). These ranged from 46 (Cys) up to 2,541 (Val). We identified 174 loci that share homology with 34 known small nucleolar RNA (snoRNA) families; these included 15 small Cajal body–specific RNA, 41 H/ACA box, and 118 C/D box snoRNA-associated loci [10]. The snoRNAs are predominantly involved in rRNA maturation. We identified 7,049 loci that share homology with 283 families of microRNA. Some of these may be of limited reliability because CMs for simple hairpin structures can also match other, non-homologous, hairpin-like structures in the genome, e.g., inverted repeats. A number of *cis*-regulatory elements were also identified. These included 235 hammerhead 1 ribozymes, 133 Histone 30 untranslated region stem-loops, and 14 potassium channel RNA editing signal sequences. There are very few matches to obvious non-metazoan RNA families in the current assemblies. The only notable exceptions are bablM, IMES-2, PhotoRC-II, and rspL. Each of these families are also found in marine metagenomic datasets, possibly explaining their presence as “contamination” from the environment.

### Analyses of specific gene families

Several gene families involved in development, such as transcription factors or signaling ligands, are highly conserved across metazoans and may therefore reveal signatures of genomic events, such as a whole-genome duplication.

WNT is a family of secreted lipid-modified signaling glycoproteins that plays a key role during development [57]. Comparative analysis of molluscan genomes indicates that the ancestral state was 12 *WNT* genes, as *Wnt3* is absent in all protostomes examined thus far [58]. The giant squid has the typical 12 lophotrochozoan WNTs (1, 2, 4, A, 5, 6, 7, 8, 9, 10, 11, and 16; Supplementary Fig. S2) and therefore has retained the ancestral molluscan complement, including *Wnt8*, which is absent, for instance, in the genome of the slipper snail *Lottia gigantea* [59].

Protocadherins are a family of cell adhesion molecules that seem to play an important role in vertebrate brain development [60]. It is thought that they act as multimers at the cell surface in a manner akin to DSCAM in flies, which lack protocadherins [61]. Cephalopods have massively expanded this family, with 168 identified in the *O. bimaculoides* genome, whereas only 17–25 protocadherins have been identified in the genomes of annelids and non-cephalopod molluscs [11]. We identified ∼135 protocadherin genes in *A. dux*, many of which are located in clusters in the genome. The possibility that this gene family plays a developmental role parallel to that of protocadherins in vertebrate neurodevelopment thus remains a compelling hypothesis.

**Figure 3:**
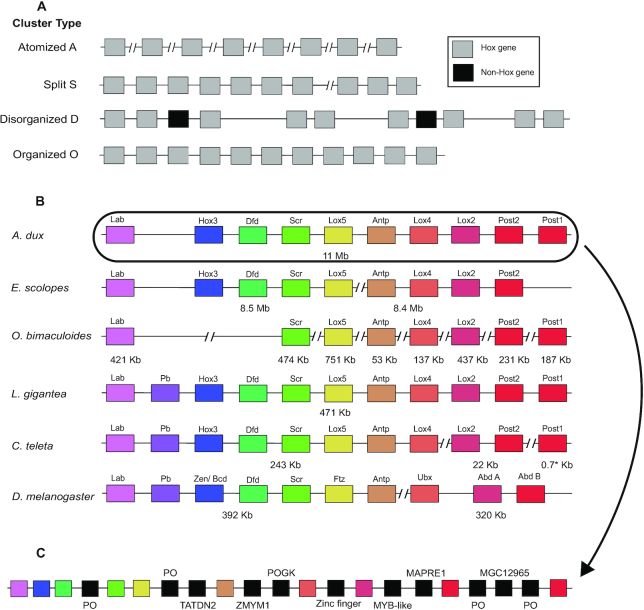
Schematic representation of the Hox gene clusters. Different scaffolds are separated by 2 slashes. **(A)** Simplified classification of the Hox clusters genomic organization. Type A identifies the lack of a “typical” Hox cluster configuration, i.e., genes are scattered through the genome (not closely placed); Type S indicates a Hox cluster that is separated by a chromosomal breakpoint; Type D clusters comprehend all the genes in the same location but encompassing a larger region than in organized clusters and may display non-Hox genes and repeats in between; Type O indicates a very compact cluster embracing a short region with only Hox genes. Non-coding RNA and microRNA can be found. **(B)** Simplified scheme of the chromosomal organization in various invertebrates. Scaffold length is shown underneath. Unlike in other coleoids, for *Architeuthis dux* all Hox genes were found in the same scaffold. However, the distance between the genes was larger than expected for invertebrate organisms, and non-homeobox genes were also present within the cluster. Hox2 remains undetected in coleoids. *A. dux* cluster can be found in scaffold 25. *E. scolopes, O. bimaculoides, L. gigantea, C. teleta*, and *D. melanogaster* assemblies and Hox cluster details can be found in [11, 53, 59, 70]. The asterisk indicates a gene that was reported in a different scaffold, adjacent to non-Hox genes (the length corresponds to the size of the gene). **(C)** Complete representation of the Hox cluster found in *A. dux* including the non-Hox genes. PO—predicted open reading frame; TATDN2–putative deoxyribonuclease TATDN2; ZMYM1–zinc finger MYM-type protein 1; POGK—pogo transposable element with KRAB; zinc finger—zinc finger protein; MYB-like—putative Myb-like DNA-binding domain protein; MAPRE1–microtubule-associated protein RP/EB family member 1; MGC12965–similar to cytochrome c, somatic.

Development organization of the highly diverse body plans found in the Metazoa is controlled by a conserved cluster of homeotic genes, which includes, among others, the Hox genes. These are characterized by a DNA sequence referred to as the homeobox, comprising 180 nucleotides that encode the homeodomain [62]. Hox genes are usually found in tight physical clusters in the genome and are sequentially expressed in the same chronological order as they are physically located in the DNA (temporal and spatial collinearity) [63]. Different combinations of Hox gene expression in the same tissue type can lead to a wide variety of different structures [64]. This makes the Hox genes a key subject for understanding the origins of the multitude of forms found in the cephalopods. In the *O. bimaculoides* genome assembly no scaffold contained more than a single Hox gene, meaning that they are fully atomized [11]. However, in *E. scolopes*, the Hox cluster was found spanning 2 scaffolds [53]. In the giant squid, we recovered a full Hox gene cluster in a single scaffold (Fig. 3B). The Hox gene organization found in the giant squid genome suggests either the presence of a disorganized cluster, so-called type D, or atomized clusters, type A [64], or possibly a combination of the two (the genes are still organized but physically distant from each other). The existence of a “true” cluster seems unlikely, given the presence of other unrelated genes in between and the relatively large distances (Fig. 3C). The classification as type A (atomized) might seem most obvious, despite the co-presence of the genes in a single scaffold, due to these large distances. However, the definition of type D (disorganized) does allow for the presence of non-Hox genes in between members of the cluster (Fig. 3A). Thus, it is difficult to clearly categorize the recovered “cluster,” but it does remain clear that these genes are not as tightly bundled as they are in other Bilateria lineages. The *A. dux* Hox cluster is spread across 11 Mb of a 38-Mb scaffold, and this suggests a far larger size range in the cephalopods than in other described animals, as recently suggested based on the genome of *E. scolopes* [53]. It is possible that this is the reason for the apparent atomization of Hox genes in the more fragmented *O. bimaculoides* assembly. Hox clusters are usually found in contigs of ∼100 kb length in vertebrates [6, 7] and between 500 and 10,000 kb in invertebrates [8]. An assembled contig easily containing the complete cluster for these smaller cluster sizes would manage to cover only 1 member of the Hox gene cluster in the studied coleoids. As such, our results suggest that the Hox cluster may not be fully atomized in *O. bimaculoides* as previously hypothesized. Further improvements of genome assemblies in cephalopods will be required to address this question. The biological reason for this dramatic increase in the distance between the genes in the Hox cluster presents an intriguing avenue of future research. The homeodomain of all the obtained Hox genes in cephalopods was compared with those of other mollusks. Few differences were found relative to a previous study [65] because no significant modifications were observed in Hox1, Hox4, ANTP, Lox2, Lox5, Post1, and Post2. Hox1 did, however, show reduced conservation in residues 22–25 in the *A. dux* sequence. This observation for Hox1 in *A. dux* is visible only in the PacBio assembly. Additionally, the Hox3 homeodomain analysis supports a basal placement of the nautiloids within cephalopods. The Lox4 gene was the most variable among all groups. To date, Hox2 remains undetected in the coleoid cephalopods [66]. Assembly errors notwithstanding, gain and loss of Hox genes has been attributed to fundamental changes in animal body plans, and the apparent loss of Hox2 may therefore be significant. For example, Hox gene loss has been associated with the reduced body-plan segmentation of spider mites [43]. The circumstance that Hox2 has been readily found in *Nautilus*, but remains undetected in all coleoids sequenced thus far, might signify an important developmental split within the Cephalopoda. Alternatively, and equally intriguing, this Hox gene may have undergone such drastic evolutionary modifications that it is presently undetectable by conventional means.

On a final note, we analyzed genes encoding reflectins, a class of cephalopod-specific proteins first described in *E. scolopes* [67]. Reflectins form flat structures that reflect ambient light (other marine animals use purine-based platelets), thus modulating iridescence for communication or camouflage purposes [68]. The giant squid genome contains 7 reflectin genes and 3 reflectin-like genes (Supplementary Fig. S3). All of these genes, with the exception of 1 reflectin gene, appear on the same scaffold, which corresponds very well with the distribution pattern of octopus reflectin genes [11].

## Conclusions

Not only because of its astonishing proportions, but also for the lack of knowledge of the key facets of its deep-sea lifestyle, the giant squid has long captured the imagination of scientists and the general public alike. With the release of this annotated giant squid genome, we set the stage for future research into the enigmas that enshroud this awe-inspiring creature. Furthermore, given the paucity of available cephalopod genomes, we provide a valuable contribution to the genomic description of cephalopods, and more widely to the growing number of fields that are recognizing the potential that this group of behaviourally advanced invertebrates holds for improving our understanding of the diversity of life on Earth in general.

## Availability of Supporting Data and Materials

The datasets supporting the results of this article are available in the NCBI database via Bioproject PRJNA534469. The 3 transcriptome datasets (TSA) have IDs GHKK01000000, GHKL01000000, and GHKH01000000 and the sequence data used for the genome assemblies have ID VCCN01000000. Proteomics data are available via ProteomeXchange with identifier PXD016522. Supporting data are also available via the *Gigascience* repository GigaDB [69].

## Additional Files

Supplementary Methods:

DNA extraction
MALDI-TOF/TOF and protein identification
Repeat assembly from raw reads with REPdenovo
Repeat assembly from raw reads with RaAS

Supplementary Figures:

Figure S1. Sample details.
Figure S2. Phylogenetic tree: WNTs.
Figure S3. Phylogenetic tree: reflectins.
Figure S4. Size distribution of protein-coding genes.

Supplementary Tables:

Table S1. Sequence data.
Table S2. Assembly statistics.
Table S3. Copy numbers of RNA isotypes.
Table S4. Repeat summary.

giz152_GIGA-D-19-00236_Original_Submission

giz152_GIGA-D-19-00236_Revision_1

giz152_GIGA-D-19-00236_Revision_2

giz152_Response_to_Reviewer_Comments_Original_Submission

giz152_Response_to_Reviewer_Comments_Revision_1

giz152_Reviewer_1_Report_Original_Submission Kevin Kocot -- 7/25/2019 Reviewed

giz152_Reviewer_1_Report_Revision_1 Kevin Kocot -- 11/2/2019 Reviewed

giz152_Reviewer_2_Report_Original_Submission Yi-Jyun Luo -- 7/25/2019 Reviewed

giz152_Reviewer_2_Report_Revision_1 Yi-Jyun Luo -- 10/31/2019 Reviewed

giz152_Supplemental_File

## Abbreviations

aa: amino acids; BLAST: Basic Local Alignment Search Tool; bp: base pairs; BUSCO: Benchmarking Universal Single-copy Orthologs; CDS: coding sequence; CM: covariance model; DIRS: *Dictyostelium* intermediate repeat sequence 1 - like elements; DTT: dithiothreitol; Gb: gigabase pairs; kb: kilobase pairs; LC-MS/MS: liquid chromatography tandem mass spectrometry; LINE: long interspersed nuclear element; LTR: long terminal repeat; MALDI-TOF: matrix-assisted laser desorption/ionization-time of flight; Mb: megabase pairs; NCBI: National Center for Biotechnology Information; ncRNA: non-coding RNA; PAGE: polyacrylamide gel electrophoresis; rRNA: ribosomal RNA; SDS: sodium dodecyl sulfate; SINE: short interspersed nuclear element; SMRT: single-molecule real-time sequencing; snoRNA: small nucleolar RNA; TE: transposable element; tRNA: transfer RNA.

## Ethics Statement

Sampling followed the recommendations from Moltschaniwskyj et al. 2007 [23].

## Competing interests

The authors declare that they have no competing interests.

## Funding

R.R.F. thanks the Villum Fonden for grant VKR023446 (Villum Fonden Young Investigator Grant), the Portuguese Science Foundation (FCT) for grant PTDC/MAR/115347/2009; COMPETE-FCOMP-01-012; FEDER-015453, Marie Curie Actions (FP7-PEOPLE-2010-IEF, Proposal 272927), and the Danish National Research Foundation (DNRF96) for its funding of the Center for Macroecology, Evolution, and Climate. H.O. thanks the Rede Nacional de Espectrometria de Massa, ROTEIRO/0028/2013, ref. LISBOA-01-0145-FEDER-022125, supported by COMPETE and North Portugal Regional Operational Programme (Norte2020), under the PORTUGAL 2020 Partnership Agreement, through the European Regional Development Fund (ERDF). A.C. thanks FCT for project UID/Multi/04423/2019. M.P. acknowledges the support from the Wellcome Trust (grant number WT108749/Z/15/Z) and the European Molecular Biology Laboratory. M.P.T.G. thanks the Danish National Research Foundation for its funding of the Center for GeoGenetics (grant DNRF94) and Lundbeck Foundation for grant R52–5062 on Pathogen Palaeogenomics. S.R. was supported by the Novo Nordisk Foundation grant NNF14CC0001. A.H. is supported by a Biotechnology and Biological Sciences Research Council David Phillips Fellowship (fellowship reference: BB/N020146/1). T.B. is supported by the Biotechnology and Biological Sciences Research Council-funded South West Biosciences Doctoral Training Partnership (training grant reference BB/M009122/1). This work was partially funded by the Lundbeck Foundation (R52-A4895 to B.B.).

H.J.T.H. was supported by the David and Lucile Packard Foundation, the Netherlands Organization for Scientific Research (#825.09.016), and currently by the Deutsche Forschungsgemeinschaft (DFG) under grant HO 5569/2-1 (Emmy Noether Junior Research Group). T.V. and B. Brejova were supported by grants from the Slovak grant agency VEGA (1/0684/16, 1/0458/18). F.S. was supported by a PhD grant (SFRH/BD/126560/2016) from FCT. A.A. was partly supported by the FCT project PTDC/CTA-AMB/31774/2017. C.C. and Y.W. are partly supported by grant IIS-1526415 from the US National Science Foundation. Computation for the work described in this article was partially supported by the DeiC National Life Science Supercomputer at DTU.

## Authors' Contributions

R.D.F. and M.T.P.G. designed the study. J.S., H-J.H., and R.T. carried out the sampling. A. Campos, A.R., B.F., G.C.A., H.O., and I.W. performed the laboratory work. R.D.F., A. Couto, A.M., C.B.A., F.S., P.G., T.B., A.H., I.B.H., C.C., B.P., F.P., M.P., F.M., O.S., S.R., M.Z.R., and D.P. analyzed the data. E.J., G.Z., J.V., O.F., and Q.L. contributed with genomic resources. R.D.F., L.F.C.C., A.A., Y.W., B.M., R.S., T.V., B.B., T.S-P., and M.T.P.G. contributed with supervision and computational resources. R.R.F., T.S-P., R.N., and M.T.P.G paid for sequencing. R.D.F. wrote the manuscript with contributions from all authors. All authors have read and approved the manuscript.

## References

[1] Zullo L, Hochner B A new perspective on the organization of an invertebrate brain. Commun Integr Biol. 2011;4:26–9. 21509172 10.4161/cib.4.1.13804 PMC3073264

[2] Nixon M, Young JZ The Brains and Lives of Cephalopods. Oxford, UK: Oxford University Press; 2003.

[3] Doubleday ZA, Prowse TAA, Arkhipkin A, et al. Global proliferation of cephalopods. Curr Biol. 2016;26:R406–7. 27218844 10.1016/j.cub.2016.04.002

[4] Vecchione M, Allcock L, Piatkowski U, et al. Persistent elevated abundance of octopods in an overfished Antarctic area. In: Krupnik I, Lang MA, Miller SE, eds. Smithsonian at the Poles: Contributions to International Polar Year Science. Washington, DC: Smithsonian; 2009:197–204.

[5] Young RE, Vecchione M, Mangold KM Cephalopoda, Cuvier 1797. Tree of Life 2018 http://tolweb.org/Cephalopoda. Accessed 2 May 2019.

[6] Roper CF, Sweeney MJ, Nauen CE FAO Species Catalogue Vol. 3. Cephalopods of the World. An annotated and illustrated catalogue of species of interest to fisheries. Rome, Italy: Food and Agriculture Organization of the United Nations 1984;125:277.

[7] Jereb P, Roper CFE Cephalopods of the World. An annotated and illustrated catalogue of cephalopod species known to date. Myopsid and Oegopsid Squids. Rome, Italy: Food and Agriculture Organization of the United Nations 2010;2:605.

[8] McClain CR, Balk MA, Benfield MC, et al. Sizing ocean giants: patterns of intraspecific size variation in marine megafauna. PeerJ. 2015;3:e715. 25649000 10.7717/peerj.715 PMC4304853

[9] Paxton CGM Unleashing the Kraken: on the maximum length in giant squid (*Architeuthis* sp.). J Zool. 2016;300:82–8.

[10] Rosa R, Seibel BA Slow pace of life of the Antarctic colossal squid. J Mar Biol Assoc United Kingdom. 2010;90:1375–8.

[11] Albertin CB, Simakov O, Mitros T, et al. The octopus genome and the evolution of cephalopod neural and morphological novelties. Nature. 2015;524:220–4. 26268193 10.1038/nature14668 PMC4795812

[12] Liscovitch-Brauer N, Alon S, Porath HT, et al. Trade-off between transcriptome plasticity and genome evolution in cephalopods. Cell. 2017;169:191–202.e11. 28388405 10.1016/j.cell.2017.03.025 PMC5499236

[13] Gilly WF, Beman JM, Litvin SY, et al. Oceanographic and biological effects of shoaling of the oxygen minimum zone. Ann Rev Mar Sci. 2013;5:393–420. 10.1146/annurev-marine-120710-100849 22809177

[14] Golikov AV, Sabirov RM, Lubin PA, et al. Changes in distribution and range structure of Arctic cephalopods due to climatic changes of the last decades. Biodiversity. 2013;14:28–35.

[15] Balmaseda MA, Trenberth KE, Källén E Distinctive climate signals in reanalysis of global ocean heat content. Geophys Res Lett. 2013;40:1754–9.

[16] Bustamante P, González AF, Rocha F, et al. Metal and metalloid concentrations in the giant squid *Architeuthis dux* from Iberian waters. Mar Environ Res. 2008;66:278–87. 18514304 10.1016/j.marenvres.2008.04.003

[17] Unger MA, Harvey E, Vadas GG, et al. Persistent pollutants in nine species of deep-sea cephalopods. Mar Pollut Bull. 2008;56:1498–500. 18501382 10.1016/j.marpolbul.2008.04.018

[18] Winkelmann I, Campos PF, Strugnell J, et al. Mitochondrial genome diversity and population structure of the giant squid *Architeuthis*: genetics sheds new light on one of the most enigmatic marine species. Proceedings Biol Sci. 2013;280:20130273. 10.1098/rspb.2013.0273 PMC3619516 23516246

[19] Chikhi R, Medvedev P Informed and automated k-mer size selection for genome assembly. Bioinformatics. 2014;30:31–7. 23732276 10.1093/bioinformatics/btt310

[20] Chapman Ja, Ho I, Sunkara S, et al. Meraculous: de novo genome assembly with short paired-end reads. PLoS One. 2011;6:e23501. 21876754 10.1371/journal.pone.0023501 PMC3158087

[21] English AC, Richards S, Han Y, et al. Mind the gap: upgrading genomes with Pacific Biosciences rs long-read sequencing technology. PLoS One. 2012;7:e47768. 23185243 10.1371/journal.pone.0047768 PMC3504050

[22] Simão FA, Waterhouse RM, Ioannidis P, et al. BUSCO: assessing genome assembly and annotation completeness with single-copy orthologs. Bioinformatics. 2015;31:3210–2. 26059717 10.1093/bioinformatics/btv351

[23] Moltschaniwskyj NA, Hall K, Lipinski MR, et al. Ethical and welfare considerations when using cephalopods as experimental animals. Rev Fish Biol Fish. 2007;17:455–76.

[24] Patel RK, Jain M NGS QC toolkit: a toolkit for quality control of next generation sequencing data. PLoS One. 2012;7:e30619. 22312429 10.1371/journal.pone.0030619 PMC3270013

[25] Martin M Cutadapt removes adapter sequences from high-throughput sequencing reads. EMBnet J. 2011;17:10–2.. :

[26] FASTX-Toolkit. http://hannonlab.cshl.edu/fastx_toolkit. Accessed January 2014.

[27] Haas BJ, Papanicolaou A, Yassour M, et al. De novo transcript sequence reconstruction from RNA-seq using the Trinity platform for reference generation and analysis. Nat Protoc. 2013;8:1494–512. 23845962 10.1038/nprot.2013.084 PMC3875132

[28] Gilbert D Gene-omes built from mRNA seq not genome DNA. Poster presented at: 7th Annual Arthropod Genomics Symposium, Notre Dame, IN 2013.

[29] Kleffmann T, Russenberger D, von Zychlinski A, et al. The *Arabidopsis thaliana* chloroplast proteome reveals pathway abundance and novel protein functions. Curr Biol. 2004;14:354–62. 15028209 10.1016/j.cub.2004.02.039

[30] Bradford MM A rapid and sensitive method for the quantitation of microgram quantities of protein utilizing the principle of protein-dye binding. Anal Biochem. 1976;72:248–54. 942051 10.1016/0003-2697(76)90527-3

[31] Santos R, da Costa G, Franco C, et al. First insights into the biochemistry of tube foot adhesive from the sea urchin *Paracentrotus lividus* (Echinoidea, Echinodermata). Mar Biotechnol. 2009;11:686–98. 19221839 10.1007/s10126-009-9182-5

[32] Neuhoff V, Arold N, Taube D, et al. Improved staining of proteins in polyacrylamide gels including isoelectric focusing gels with clear background at nanogram sensitivity using Coomassie Brilliant Blue G-250 and R-250. Electrophoresis. 1988;9:255–62. 2466658 10.1002/elps.1150090603

[33] Brejová B, Vinar T, Chen Y, et al. Finding genes in *Schistosoma japonicum*: annotating novel genomes with help of extrinsic evidence. Nucleic Acids Res. 2009;37:e52. 19264800 10.1093/nar/gkp052 PMC2673418

[34] Korf I Gene finding in novel genomes. BMC Bioinformatics. 2004;5:59. 15144565 10.1186/1471-2105-5-59 PMC421630

[35] Lomsadze A, Ter-Hovhannisyan V, Chernoff YO, et al. Gene identification in novel eukaryotic genomes by self-training algorithm. Nucleic Acids Res. 2005;33:6494–506. 16314312 10.1093/nar/gki937 PMC1298918

[36] Musacchia F, Basu S, Petrosino G, et al. Annocript: a flexible pipeline for the annotation of transcriptomes able to identify putative long noncoding RNAs. Bioinformatics. 2015;31:2199–201. 25701574 10.1093/bioinformatics/btv106

[37] Camacho C, Coulouris G, Avagyan V, et al. BLAST+: architecture and applications. BMC Bioinformatics. 2009;10:421. 20003500 10.1186/1471-2105-10-421 PMC2803857

[38] Burge SW, Daub J, Eberhardt R, et al. Rfam 11.0: 10 years of RNA families. Nucleic Acids Res. 2013;41:D226–32. 23125362 10.1093/nar/gks1005 PMC3531072

[39] Gardner PP, Daub J, Tate J, et al. Rfam: Wikipedia, clans and the "decimal" release. Nucleic Acids Res. 2011;39:D141–5. 21062808 10.1093/nar/gkq1129 PMC3013711

[40] Freyhult EK, Bollback JP, Gardner PP Exploring genomic dark matter: a critical assessment of the performance of homology search methods on noncoding RNA. Genome Res. 2007;17:117–25. 17151342 10.1101/gr.5890907 PMC1716261

[41] Chan PP, Lowe TM GtRNAdb: a database of transfer RNA genes detected in genomic sequence. Nucleic Acids Res. 2009;37:D93–7. 18984615 10.1093/nar/gkn787 PMC2686519

[42] Lowe TM, Eddy SR tRNAscan-SE: a program for improved detection of transfer RNA genes in genomic sequence. Nucleic Acids Res. 1997;25:955–64. 9023104 10.1093/nar/25.5.955 PMC146525

[43] Smit AFA, Hubley RR, Green PR RepeatMasker Open-4.0, www.repeatmasker.org. Accessed April 2019 2013.

[44] Bao W, Kojima KK, Kohany O Repbase Update, a database of repetitive elements in eukaryotic genomes. Mob DNA. 2015;6:11. 26045719 10.1186/s13100-015-0041-9 PMC4455052

[45] Smit A, Hubley R RepeatModeler Open-1.0 2015 www.repeatmasker.org. Accessed April 2019.

[46] Bao Z, Eddy SR Automated de novo identification of repeat sequence families in sequenced genomes. Genome Res. 2002;12:1269–76. 12176934 10.1101/gr.88502 PMC186642

[47] Price AL, Jones NC, Pevzner PA De novo identification of repeat families in large genomes. Bioinformatics. 2005;21:i351–8. 15961478 10.1093/bioinformatics/bti1018

[48] Platt RN, Blanco-Berdugo L, Ray DA Accurate transposable element annotation is vital when analyzing new genome assemblies. Genome Biol Evol. 2016;8:403–10. 26802115 10.1093/gbe/evw009 PMC4779615

[49] Schnable PS, Ware D, Fulton RS, et al. The B73 maize genome: complexity, diversity, and dynamics. Science. 2009;326:1112–5. 19965430 10.1126/science.1178534

[50] Wu C, Twort VG, Crowhurst RN, et al. Assembling large genomes: analysis of the stick insect (*Clitarchus hookeri*) genome reveals a high repeat content and sex-biased genes associated with reproduction. BMC Genomics. 2017;18:884. 29145825 10.1186/s12864-017-4245-x PMC5691397

[51] Lander ES, Linton LM, Birren B Initial sequencing and analysis of the human genome. Nature. 2001;409:860–921. 11237011 10.1038/35057062

[52] Kim B-M, Kang S, Ahn D-H, et al. The genome of common long-arm octopus *Octopus minor*. Gigascience. 2018;7, doi:10.1093/gigascience/giy119. PMC6279123 30256935

[53] Belcaid M, Casaburi G, McAnulty SJ, et al. Symbiotic organs shaped by distinct modes of genome evolution in cephalopods. Proc Natl Acad Sci U S A. 2019;116:3030–5. 30635418 10.1073/pnas.1817322116 PMC6386654

[54] Schrader L, Schmitz J The impact of transposable elements in adaptive evolution. Mol Ecol. 2018;28:1537–49. 30003608 10.1111/mec.14794

[55] Zarrella I, Herten K, Maes GE, et al. The survey and reference assisted assembly of the *Octopus vulgaris* genome. Sci Data. 2019;6:13. 30931949 10.1038/s41597-019-0017-6 PMC6472339

[56] Nawrocki EP, Burge SW, Bateman A, et al. Rfam 12.0: updates to the RNA families database. Nucleic Acids Res. 2015;43:D130–7. 25392425 10.1093/nar/gku1063 PMC4383904

[57] Cadigan KM, Nusse R Wnt signaling: a common theme in animal development. Genes Dev. 1997;11:3286–305. 9407023 10.1101/gad.11.24.3286

[58] Cho S-J, Valles Y, Giani VC, et al. Evolutionary dynamics of the wnt gene family: a Lophotrochozoan perspective. Mol Biol Evol. 2010;27:1645–58. 20176615 10.1093/molbev/msq052 PMC2912473

[59] Simakov O, Marletaz F, Cho S-J, et al. Insights into bilaterian evolution from three spiralian genomes. Nature. 2012;493:526–31. 23254933 10.1038/nature11696 PMC4085046

[60] Chen WV, Maniatis T Clustered protocadherins. Development. 2013;140:3297–302. 23900538 10.1242/dev.090621 PMC3737714

[61] Zipursky SL, Sanes JR Chemoaffinity revisited: dscams, protocadherins, and neural circuit assembly. Cell. 2010;143:343–53. 21029858 10.1016/j.cell.2010.10.009

[62] Pratihar S, Prasad Nath R, Kumar Kundu J Hox genes and its role in animal development. Int J Sci Nat. 2010;1:101–3.

[63] Fröbius AC, Matus DQ, Seaver EC Genomic organization and expression demonstrate spatial and temporal hox gene colinearity in the Lophotrochozoan *Capitella* sp. PLoS One. 2008;3:e4004. 19104667 10.1371/journal.pone.0004004 PMC2603591

[64] Mallo M, Wellik DM, Deschamps J Hox genes and regional patterning of the vertebrate body plan. Dev Biol. 2010;344:7–15. 20435029 10.1016/j.ydbio.2010.04.024 PMC2909379

[65] Pernice M, Deutsch JS, Andouche A, et al. Unexpected variation of Hox genes’ homeodomains in cephalopods. Mol Phylogenet Evol. 2006;40:872–9. 16759883 10.1016/j.ympev.2006.04.004

[66] Barucca M, Canapa A, Biscotti MA, et al. An overview of hox genes in Lophotrochozoa: evolution and functionality. J Dev Biol. 2016;4:1–15. 10.3390/jdb4010012 PMC5831810 29615580

[67] Crookes WJ, Ding L-L, Huang QL, et al. Reflectins: the unusual proteins of squid reflective tissues. Science. 2004;303:235–8. 14716016 10.1126/science.1091288

[68] Wardill TJ, Gonzalez-Bellido PT, Crook RJ, et al. Neural control of tuneable skin iridescence in squid. Proc R Soc B Biol Sci. 2012;279:4243–52. 10.1098/rspb.2012.1374 PMC3441077 22896651

[69] da Fonseca RR, Couto A, Machado AM, et al. Supporting data for “A draft genome sequence of the elusive giant squid, *Architeuthis dux*.”. GigaScience Database. 2019 10.5524/100676. PMC6962438 31942620

[70] Pace RM, Grbić M, Nagy LM Composition and genomic organization of arthropod Hox clusters. Evodevo. 2016;7:11. 27168931 10.1186/s13227-016-0048-4 PMC4862073

