# Supplementary material for: A draft genome sequence of the elusive giant squid, Architeuthis dux
Source: Gigascience. 2020 Jan 16;9(1):giz152. doi: 10.1093/gigascience/giz152 (PMC6962438; doi:10.1093/gigascience/giz152)
Supplement: giz152_GIGA-D-19-00236_Original_Submission [file giz152_giga-d-19-00236_original_submission.pdf]

GigaScience

# A draft genome sequence of the elusive giant squid, Architeuthis dux

--Manuscript Draft--

| | |
|---|---|
| **Manuscript Number:** | GIGA-D-19-00236 |
| **Full Title:** | A draft genome sequence of the elusive giant squid, Architeuthis dux |
| **Article Type:** | Data Note |

**Funding Information:**



| | |
|---|---|
| Villum Fonden (VKR023446) | Dr Rute R. da Fonseca |
| FP7 People: Marie-Curie Actions (272927) | Dr Rute R. da Fonseca |
| Fundação para a Ciência e a Tecnologia (PTDC/MAR/115347/2009) | Dr Rute R. da Fonseca |
| Danmarks Grundforskningsfond (DNRF96) | Dr Rute R. da Fonseca |
| Programa Operacional Temático Factores de Competitividade (PT) (COMPETE-FCOMP-01-012) | Dr Rute R. da Fonseca |
| Rede Nacional de Espectrometria de Massa (ROTEIRO/0028/2013) | Dr Hugo Osorio |
| Fundação para a Ciência e a Tecnologia (UID/Multi/04423/2019) | Dr Alexandre Campos |
| Wellcome Trust (WT108749/Z/15/Z) | Dr Mateus Patricio |
| Danmarks Grundforskningsfond (DNRF94) | Dr M. Thomas P. Gilbert |
| Lundbeckfonden (R52-5062) | Dr M. Thomas P. Gilbert |
| Novo Nordisk Fonden (NNF14CC0001) | Dr Simon Rasmussen |
| Biotechnology and Biological Sciences Research Council (BB/N020146/1) | Dr Alex Hayward |
| Biotechnology and Biological Sciences Research Council (BB/M009122/1) | Dr Tobias Baril |
| Lundbeckfonden (R52-A4895) | Dr Blagoy Blagoev |
| Nederlandse Organisatie voor Wetenschappelijk Onderzoek (NL) (#825.09.016) | Dr Henk-Jan Hoving |
| Deutsche Forschungsgemeinschaft (DE) (HO 5569/1-2) | Dr Henk-Jan Hoving |
| Slovak grant agency VEGA (VEGA 1/0684/16) | Dr Brona Brejova |
| Slovak grant agency VEGA (VEGA 1/0458/18) | Dr Tomas Vinar |



**Abstract:**


Background

The giant squid (Architeuthis dux; Steenstrup, 1857) is an enigmatic giant mollusk with a circumglobal distribution in the deep ocean, except in the high Arctic and Antarctic waters. The elusiveness of the species makes it difficult to study. Thus, having a genome assembled for this deep-sea dwelling species will allow unlocking several pending evolutionary questions.

Findings

We present a draft genome assembly that includes 200 Gb of Illumina reads, 4 Gb of Moleculo synthetic long-reads and 108 Gb of Chicago libraries, with a final size matching the estimated genome size of 2.7 Gb, and a scaffold N50 of 4.8 Mb. We also


Powered by Editorial Manager® and ProduXion Manager® from Aries Systems Corporation

| | |
|---|---|
| | present an alternative assembly including 27 Gb raw reads generated using the Pacific Biosciences platform. In addition, we sequenced the proteome of the same individual and RNA from three different tissue types from three other species of squid to assist genome annotation. We annotated 51,225 unique protein coding genes, from which 30,472 have transcript evidence. Genome completeness estimated by BUSCO reached 92%. Repetitive regions cover 49.17% of the genome.<br><br>Conclusions<br><br>This annotated draft genome of A. dux provides a critical resource to investigate the unique traits of this species, including its gigantism and key adaptations to deep-sea environments. |
| **Corresponding Author:** | Rute R. da Fonseca<br>University of Copenhagen<br>Copenhagen, DENMARK |
| **Corresponding Author Secondary Information:** | |
| **Corresponding Author's Institution:** | University of Copenhagen |
| **Corresponding Author's Secondary Institution:** | |
| **First Author:** | Rute R. da Fonseca |
| **First Author Secondary Information:** | |
| **Order of Authors:** | Rute R. da Fonseca |
| | Alvarina Couto |
| | Andre Machado |
| | Brona Brejova |
| | Caroline B. Albertin |
| | Filipe Silva |
| | Paul Gardner |
| | Tobias Baril |
| | Alex Hayward |
| | Alexandre Campos |
| | Angela Ribeiro |
| | Inigo Barrio Hernandez |
| | Henk-Jan Hoving |
| | Ricardo Tafur-Jimenez |
| | Chong Chu |
| | Barbara Frazao |
| | Bent Petersen |
| | Fernando Penalosa |
| | Francesco Musacchia |
| | Graham C. Alexander Jr. |
| | Hugo Osorio |
| | Inger Winkelmann |
| | Oleg Simakov |
| | |

| | |
|---|---|
| | Simon Rasmussen |
| | M. Ziaur Rahman |
| | Davide Pisani |
| | Erich Jarvis |
| | Guojie Zhang |
| | Jakob Vinther |
| | Jan Strugnell |
| | L. Filipe C. Castro |
| | Olivier Fedrigo |
| | Mateus Patricio |
| | Qiye Li |
| | Sara Rocha |
| | Agostinho Antunes |
| | Yufeng Wu |
| | Bin Ma |
| | Remo Sanges |
| | Tomas Vinar |
| | Blagoy Blagoev |
| | Thomas Sicheritz-Ponten |
| | Rasmus Nielsen |
| | M. Thomas P. Gilbert |
| **Order of Authors Secondary Information:** | |
| **Additional Information:** | |
| **Question** | **Response** |
| Are you submitting this manuscript to a special series or article collection? | No |
| **Experimental design and statistics**<br><br>Full details of the experimental design and statistical methods used should be given in the Methods section, as detailed in our Minimum Standards Reporting Checklist. Information essential to interpreting the data presented should be made available in the figure legends.<br><br>Have you included all the information requested in your manuscript? | Yes |
| **Resources** | Yes |

Powered by Editorial Manager® and ProduXion Manager® from Aries Systems Corporation

| | |
|---|---|
| A description of all resources used, including antibodies, cell lines, animals and software tools, with enough information to allow them to be uniquely identified, should be included in the Methods section. Authors are strongly encouraged to cite Research Resource Identifiers (RRIDs) for antibodies, model organisms and tools, where possible.<br><br>Have you included the information requested as detailed in our Minimum Standards Reporting Checklist? | |
| **Availability of data and materials**<br><br>All datasets and code on which the conclusions of the paper rely must be either included in your submission or deposited in publicly available repositories (where available and ethically appropriate), referencing such data using a unique identifier in the references and in the "Availability of Data and Materials" section of your manuscript.<br><br>Have you have met the above requirement as detailed in our Minimum Standards Reporting Checklist? | Yes |

Powered by Editorial Manager® and ProduXion Manager® from Aries Systems Corporation

Main text, Table1, Figure legens Click here to download Manuscript RFonseca_main.docx

# A draft genome sequence of the elusive giant squid, *Architeuthis dux*

Rute R. da Fonseca*[1,2], Alvarina Couto[3], Andre Machado[4], Brona Brejova[5], Carolin B. Albertin[6], Filipe Silva[4], Paul Gardner[7], Toby Baril[8], Alex Hayward[8], Alexandre Campos[4], Ângela Ribeiro[4], Inigo Barrio Hernandez[9], Henk-Jan Hoving[10], Ricardo Tafur-Jimenez[11], Chong Chu[12], Barbara Frazão[4,13], Bent Petersen[14,15], Fernando Peñaloza[16], Francesco Musacchia[17], Graham C. Alexander Jr. [18], Hugo Osório[19,20,21], Inger Winkelmann[22], Oleg Simakov[23], Simon Rasmussen[24], M. Ziaur Rahman[25], Davide Pisani[26], Jakob Vinther[27], Erich Jarvis[28], Guojie Zhang[30,31,32,33], Jan Strugnell[34], L. Filipe C. Castro[4,36], Olivier Fedrigo[28], Mateus Patricio[29], Qiye Li[37], Sara Rocha[3], Agostinho Antunes[4,36], Yufeng Wu[38], Bin Ma[39], Remo Sanges[40,41], Tomas Vinar[5], Blagoy Blagoev[9], Thomas Sicheritz-Ponten[14,15], Rasmus Nielsen[22,42], M. Thomas P. Gilbert[22,43]

[1]Center for Macroecology, Evolution and Climate, Natural History Museum of Denmark, University of Copenhagen, Copenhagen, Denmark.

[2]The Bioinformatics Centre, Department of Biology, University of Copenhagen, Copenhagen, Denmark.

[3]Department of Biochemistry, Genetics and Immunology, University of Vigo, Vigo, Spain.

[4]CIIMAR, Interdisciplinary Centre of Marine and Environmental Research, University of Porto, Porto, Portugal.

[5]Faculty of Mathematics, Physics and Informatics, Comenius University in Bratislava, Bratislava, Slovak Republic.

[6]Department of Organismal Biology and Anatomy, University of Chicago, Chicago, USA.

[7]Department of Biochemistry, University of Otago, New Zealand.

[8]Centre for Ecology and Conservation, University of Exeter, Penryn Campus, Cornwall, UK.

[9]Department of Biochemistry and Molecular Biology, University of Southern Denmark, Odense, Denmark.
[10]GEOMAR Helmholtz Centre for Ocean Research Kiel, Kiel, Germany.
[11]Instituto del Mar del Perú.
[12]Department of Biomedical Informatics, Harvard Medical School, Boston, USA.
[13]IPMA, Fitoplâncton Lab, Lisboa, Portugal.
[14]Centre of Excellence for Omics-Driven Computational Biodiscovery (COMBio), Faculty of Applied Sciences, AIMST University, Kedah, Malaysia.
[17]Genomic Medicine, Telethon Institute of Genetics and Medicine, Pozzuoli, Naples, Italy
[18]GCB Sequencing and Genomic Technologies Shared Resource, Duke University, Durham, NC, USA.
[19]i3S-Instituto de Investigação e Inovação em Saúde, Universidade do Porto, Porto, Portugal.
[20]IPATIMUP -Institute of Molecular Pathology and Immunology, University of Porto, Porto, Portugal.
[21]Faculty of Medicine of the University of Porto, Porto, Portugal.
[22]Centre for GeoGenetics, Natural History Museum of Denmark, University of Copenhagen, Copenhagen, Denmark.
[23]Department of Molecular Evolution and Development, University of Vienna, Vienna, Austria.
[24]Novo Nordisk Foundation Center for Protein Research, Faculty of Health and Medical Sciences, University of Copenhagen, Copenhagen, Denmark
[25]Bioinformatics Solutions Inc, Waterloo, Ontario, Canada.
[26]Departments of Biological sciences and Earth Sciences, University of Bristol, Bristol, UK.
[27]Departments of Biological sciences and Earth Sciences, University of Bristol, Bristol, UK.
[28]The Rockefeller University, New York, USA.
[29]European Molecular Biology Laboratory, European Bioinformatics Institute (EMBL-EBI), Wellcome Genome Campus, Hinxton, UK.

[30]Section for Ecology and Evolution, Department of Biology, University of Copenhagen, Copenhagen, Denmark.
[31]China National Genebank, BGI-Shenzhen, Shenzhen, China.
[32]State Key Laboratory of Genetic Resources and Evolution, Kunming Institute of Zoology, Chinese Academy of Sciences, Kunming, China.
[33]CAS Center for Excellence in Animal Evolution and Genetics, Chinese Academy of Sciences, Kunming, China.
[34]Centre for Sustainable Tropical Fisheries & Aquaculture, James Cook University, Townsville, Queensland, Australia
[35]Department of Ecology, Environment and Evolution, School of Life Sciences, La Trobe University, Melbourne, Victoria, Australia
[36]Department of Biology, Faculty of Sciences, University of Porto, Portugal.
[37]BGI-Shenzhen, Shenzhen, China
[38]Department of Computer Science and Engineering, University of Connecticut, Storrs, USA.
[39]School of Computer Science, University of Waterloo, Canada.
[40]Area of Neuroscience, Scuola Internazionale Superiore di Studi Avanzati (SISSA), Trieste, Italy.
[41]Biology and Evolution of Marine Organisms, Stazione Zoologica Anton Dohrn, Napoli, Italy.
[42]Departments of Integrative Biology and Statistics, University of California, Berkeley, U.S.A.
[43]Norwegian University of Science and Technology, University Museum, Trondheim, Norway

Email addresses:
Rute R da Fonseca: rfonseca@bio.ku.dk (corresponding author)
Alvarina Couto: alvarinacouto@gmail.com
Andre M.Machado: andre.machado@ciimar.up.pt
Brona Brejova: brejova@fmph.uniba.sk
Caroline B.Albertin: calbertin@mbl.edu

Filipe Silva: filipecgsilva@gmail.com
Paul Gardner: paul.gardner@otago.ac.nz
Tobias Baril: tb529@exeter.ac.uk
Alex Hayward Hayward: Alex.Hayward@exeter.ac.uk
Alexandre Campos: acampos@ciimar.up.pt
Ângela Ribeiro ribeiro.angela@gmail.com
Inigo Barrio Hernandez: ibarrioh@ebi.ac.uk
Henk-Jan Hoving: hhoving@geomar.de
Ricardo Tafur-Jiménez: rtafur@imarpe.gob.pe
Chong Chu: Chong_Chu@hms.harvard.edu
Barbara Frazão: bmfrazao@gmail.com
Bent Petersen: bent.petersen@bio.ku.dk
Fernando Peñaloza: fpenaloz@lcg.unam.mx
Francesco Musacchia: f.musacchia@tigem.it
Graham C. Alexander Jr.:gca2@duke.edu
Hugo Osório: hosorio@ipatimup.pt
Inger E. Winkelmann: inger.winkelmann@gmail.com
Oleg Simakov: oleg.simakov@univie.ac.at
Simon Rasmussen: simon.rasmussen@cpr.ku.dk
M. Ziaur Rahman: zrahman@bioinfor.com
Davide Pisani: Davide.Pisani@bristol.ac.uk
Erich D. Jarvis: ejarvis@rockefeller.edu
Guojie Zhang: zhanggjconi@gmail.com
Jakob Vinther: vinther.jakob@gmail.com
Jan M. Strugnell: jan.strugnell@jcu.edu.au
L. Filipe C. Castro: filipe.castro@ciimar.up.pt
Olivier Fedrigo: ofedrigo@rockefeller.edu
Mateus Patricio: mateus@ebi.ac.uk
Qiye Li: liqiye@genomics.cn
Sara Rocha: sprocha@gmail.com
Agostinho Antunes: aantunes@ciimar.up.pt
Yufeng Wu: ywu@engr.uconn.edu
Bin Ma: binma@uwaterloo.ca
Remo Sanges: remo.sanges@gmail.com
Tomas Vinar: tomas.vinar@fmph.uniba.sk
Blagoy Blagoev: bab@bmb.sdu.dk
Thomas Sicheritz-Ponten: thomassp@bio.ku.dk
Rasmus Nielsen: rasmus_nielsen@berkeley.edu
M. Thomas P. Gilbert: tgilbert@snm.ku.dk

## Abstract

### Background

The giant squid (*Architeuthis dux*; Steenstrup, 1857) is an enigmatic giant mollusk with a circumglobal distribution in the deep ocean, except in the high Arctic and Antarctic waters. The elusiveness of the species makes it difficult to study. Thus, having a genome assembled for this deep-sea dwelling species will allow unlocking several pending evolutionary questions.

### Findings

We present a draft genome assembly that includes 200 Gb of Illumina reads, 4 Gb of Moleculo synthetic long-reads and 108 Gb of Chicago libraries, with a final size matching the estimated genome size of 2.7 Gb, and a scaffold N50 of 4.8 Mb. We also present an alternative assembly including 27 Gb raw reads generated using the Pacific Biosciences platform. In addition, we sequenced the proteome of the same individual and RNA from three different tissue types from three other species of squid to assist genome annotation. We annotated 51,225 unique protein coding genes, from which 30,472 have transcript evidence. Genome completeness estimated by BUSCO reached 92%. Repetitive regions cover 49.17% of the genome.

### Conclusions

This annotated draft genome of *A. dux* provides a critical resource to investigate the unique traits of this species, including its gigantism and key adaptations to deep-sea environments.



### Keywords

Cephalopod, invertebrate, genome assembly.

## Data description

### Context

Cephalopods are the most behaviourally complex of the invertebrate protostomes [1] . Their large, highly differentiated brains are comparable in relative size and complexity to those of vertebrates [2], as are their cognitive capabilities [1]. Cephalopods are distributed worldwide from tropical to polar marine habitats, from benthic to pelagic zones and from intertidal areas down to the abyssal parts of the deep sea, with the only exception being the Black Sea. Cephalopod populations are thought to be currently increasing locally for a variety of reasons [3], including potential predator release as a consequence of the depletion of fish stocks [4]. The class Cephalopoda contains approximately 800 species, with the vast majority belonging to the soft-bodied subclass Coleoidea (cuttlefishes, octopuses and squids), and a small handful belonging to the Nautiloidea (nautiluses) [5]. Cephalopods are ecologically important as a primary food source for marine mammals, birds and for many fish species. They are also increasingly important as a high-protein food source for humans and are a growing target for commercial fisheries and farming [6]. Cephalopods show a wide variety of morphologies, lifestyles and behaviours [7], but with the exception of the nautiluses they are characterised by having rapid growth and short lifespans, despite a considerable investment in costly sensory adaptations [2]. They range in size from the tiny pygmy squids (~2cm) to animals that are nearly 3 orders of magnitude larger, such as the giant squid, *Architeuthis dux* (at least 10-12m and reported up to 20m total length) [6,8,9], to the colossal squid, *Mesonychoteuthis hamiltoni* (maximum length remains unclear, but a recorded weight of 500kg makes it the largest known invertebrate [10]). A sophisticated adaptive body patterning system that can rapidly alter the texture, pattern, colour and brightness of its skin, facilitates a complex communication system, while also providing exceptional camouflage and mimicry [11]. Together these allow cephalopods to both avoid predators, and hunt prey highly efficiently, making them some of the top predators in the ocean. The remarkable adaptations of cephalopods also extend to their genome, with recent work demonstrating

increased levels of RNA editing to diversify proteins involved in neural functions [12].

Over recent years, oceanic warming and acidification, pollution, expanding hypoxia and fishing [13–15] have been shown to affect cephalopod populations. Depletion due to high rates of cephalopod by-catch in commercial fisheries can also result in regional extinction [16]. Mercury has been found in high concentrations in the tissue of giant squid specimens [17], and accumulation of flame retardant chemicals has also been detected in the tissue of deep-sea cephalopods [18]. Consequently, there is an urgent need for greater biological understanding of these important, but rarely encountered animals, in order to aid conservation efforts and ensure their continued existence. A genome is an important resource for future population genomics studies aiming at characterizing the diversity of the legendary giant squid, the species which has inspired generations to tell tales of the fabled Kraken.

## Methods

### *DNA extraction, library building, and de novo genome assembly*

High-molecular-weight genomic DNA was extracted from a *Architeuthis du*x (NCBI taxon id: 256136) sample using a CTAB based buffer followed by organic solvent purification, following Winkelmann et al [19] (details in the Supplementary Information). We generated 116 Gb of raw reads from Illumina short-insert libraries, 76 Gb of paired-end reads from libraries ranging from 500 bp to 800 bp in insert size, and 5.4 Gb of mate-pair with a 5 kb insert (Table S1). Furthermore, we generated 3.7 Gb of Moleculo libraries (3 High-Throughput libraries and 4 High-Fidelity libraries). The kmer distribution of the reads under a diploid model in kmergenie [20] predicted the genome size to be 2.7 Gb.

An initial assembly generated with Meraculous [21] using Illumina and Moleculo data (N50 of 32 Kb, assembly statistics in Table S2) was used as input for Dovetail Genomic's HiRise scaffolding software together with the Hi-C data generated from two Chicago libraries corresponding to a physical coverage of the genome of 52.1X. The final assembly with an N50 of 4.8 Mb (other statistics in Table 1) was used

for the genome annotation presented in this paper. The genome gene content completeness was evaluated through the Benchmarking Universal Single-Copy Orthologs (BUSCO v.3.0.2, datasets: Eukaryota, Metazoan) [22]. For Eukaryota and Metazoa we identified a total of 90.4 % and 92.1 % of BUSCO ortholog genes, respectively. Further scaffolding was done using 23.38Gb of PacBio reads (19 SMRT cells, average read length is 14.79kb) using the default parameters in PBJelly [23] (see assembly statistics in Table S2).

### *Transcriptome sequencing and de novo assembly*

Given the extreme rarity of live giant squid sightings, we were unable to collect fresh organ samples (following the recommendations in [24]) containing intact RNA from the species to assist with the genome annotation. As an alternative, we extracted total RNA from gonad, liver and brain tissue from live caught specimens of three other oegopsid squid species (*Onychoteuthis banksii*, *Dosidicus gigas*, and *Sthenoteuthis oualaniensis*; NCBI taxon ids 392296, 346249 and 34553, respectively; Supplementary Figure S1), using the Qiagen RNeasy extraction kit (Qiagen,CA, USA). The RNA integrity and quantity was measured on a Qubit fluorometer (Invitrogen, OR, USA) and on the Agilent Bioanalyzer 2100 (Agilent, CA, USA). The Illumina TruSeq Kit v.2.0 was used to isolate the mRNA and prepare cDNA libraries for sequencing, following the recommended protocol. Compatible index sequences were assigned to individual libraries to allow for multiplexing on four lanes of 100bp paired-end technology on an Illumina HiSeq 2000 flow cell. Sequencing of the cDNA libraries was done at the National High-hroughput Sequencing Center at the University of Copenhagen in Denmark. We assessed the quality of the raw reads using FastQC v0.10.0 [25]. After removing indexes and adaptors with CutAdapt [26], we trimmed the reads with the FASTX-toolkit (http://hannonlab.cshl.edu/fastx_toolkit) removing bases with a Phred-scale quality score lower than 25. Reference transcriptomes were built with Trinity [27]. This software was used with the default settings including a fixed k-mer size of 25 as suggested by the authors. Annotation of coding regions was done with the EvidencialGene pipeline [28].

*Protein extraction, separation by 1D SDS–PAGE, MALDI-TOF/TOF and Protein Identification*

Given the practical impossibility of obtaining RNA from a giant squid specimen, we produced a library of giant squid peptide sequences to guide the gene annotation process.

Proteins were solubilised from a giant squid mantle tissue sample according to the procedure described by Kleffmann et al. [29] and employing the following buffers: (1) 40 mM Tris–HCl, 5 mM MgCl2 and 1 mM DTT, pH 8.5; (2) 8 M urea, 20 mM Tris, 5 mM $MgCl_2$ and 20 mM DTT; (3) 7 M urea, 2 M thiourea, 20 mM Tris, 40 mM DTT, 2% CHAPS (w ⁄ v) and 1% Triton X-100 (v ⁄ v) and (4) 40 mM Tris, 4% SDS (w ⁄ v) and 40 mm DTT. All buffers were augmented with protease inhibitors (Halt™ Protease Inhibitor Cocktail, EDTA-Free, Thermo Scientific). Tissue samples were ground in liquid nitrogen before homogenization, or homogenized directly with ultrasound (probe sonication at 60 Hz, for 3 min) in buffer 1. Solubilised proteins were collected by ultracentrifugation at 100,000 g and 4 ºC. Each extraction was performed in duplicate for each specific buffer and extracts were pooled. Protein extracts were subsequently stored at -20 ºC. Total protein content was estimated according to the Bradford (1976) method [30].

Protein separation by 1D SDS–PAGE electrophoresis was carried out as described in Santos et al. [31]. 53 µL of sample (39 µg protein) was diluted in 72 µL of Loading Buffer (0.01% bromophenol blue, 2% SDS (Sodium-DodecylSulfate), 20% glycerol, 5% β-mercaptoethanol (w/v/v) in 62.5 mM Tris – HCl, pH 6.8). The resulting solution was heated for 3 min at 99°C. Proteins were separated by SDS–PAGE with 12% (w/v) polyacrylamide gels. Electrophoresis was carried out using the mini Protean Cell (BioRad) at a constant voltage of 150 V. The separated proteins were visualized by staining with Colloidal Coomassie Brilliant Blue (CCB) [32], and lanes were cut into 15 gel sections for subsequent LC-MS/MS analysis.

*LC–MS/MS analyses*

All samples were analysed with the Easy-nLC system (Thermo Fisher Scientific), connected online to a Q Exactive mass spectrometer (Thermo Fisher Scientific) equipped with a nanoelectrospray ion source (Thermo Fisher Scientific). Tryptic peptides were loaded in a fused silica column (75 µm inner diameter)

packed with C18 resin (3-µm beads, Reprosil, Dr. Maisch), with solvent A (0.5% acetic acid). They were then eluted with a 120 minute gradient of solvent B (80% ACN, 0.5% acetic acid) with a constant flow of 250 nL/min. The Q exactive was operated in positive mode with a capillary temperature of 250 °C, using the data dependent acquisition method, which switches from full MS scans to MS/MS scans for the 12 most intense ions. Fragmentation was achieved by higher-energy collisional dissociation (HCD) with a normalized collisional energy (NCE) of 25. Full MS ranged from 300 to 1750 m/z at a resolution of 70,000, an Automatic Gain Control (AGC) of 1e6 and a maximum injection time of 120 ms, whereas MS/MS events were scanned at a resolution of 35,000, an AGC of 1e5, maximum injection time of 124 ms, isolation windows of 2 m/z and an exclusion window of 45 seconds.

### *de novo peptide prediction*

Raw LC-MS/MS data were read using Thermo Fisher MSRawFileReader 2.2 library and imported into PEAKS Studio 7.0 and subsequently preprocessed for precursor mass and charge correction, MS/MS de-isotoping, and deconvolution. PEAKS de novo sequencing [31] was performed on each refined MS/MS spectrum with a precursor and fragment ion error tolerance of 7 ppm and 0.02 da respectively. Carbamidomethylation (Cys) was set as a fixed modification and oxidation (Met) and N-terminal Acetylation as variable modifications. At most, five variable modifications per peptide were allowed. For each tandem spectrum, five *de novo* candidates were reported along with their Local Confidence Scores (the likelihood of each amino acid assignment in a de novo candidate peptide). This score was used to determine the accuracy of the de novo peptide sequences. The top de novo peptide for each spectrum was determined by the highest Average Local Confidence score (ALC) among the candidates for that spectrum.

### *Genome annotation*

Protein-coding genes were predicted by ExonHunter [33] , combining probabilistic models of sequence features with external evidence from alignments. As external evidence, we have used proteins from Octopus bimaculatus, Crassostrea gigas (Pacific oyster) and Lottia gigantea (Giant owl limpet) and

predicted proteins encoded by the transcriptomes of the three other oegopsid species analysed. These proteins were aligned to the genome by BLASTX. De-novo identified MS/MS-based peptides were initially also used as evidence, but these were later omitted due to low coverage. Evidence from predicted repeat locations was used to discourage the model to predict genes overlapping repeats. Initially, ExonHunter was run using Drosophila parameters on a randomly chosen subset of 118 contigs longer than 200kb (total length 199Mb). Out of 12,912 exons predicted in this run, 5,716 were supported by alignment data and selected to train parameters of the model for *A. dux*, using the methods described in [33]. The final predicted gene set using this model on the entire genome contained 51,225 protein-coding genes. The function of the protein-coding genes was inferred with Annocript 0.2 [34], which is based on the results from blastp [35] runs against the SwissProt (SP) and UniRef90 (Uf). In addition, we performed an rpsblast search using matrices from the conserved domain database (CDD) to annotate specific domains present on the protein queries.

Non-coding RNAs were annotated using the cmsearch program from INFERNAL 1.1 and the covariance models (CMs) from the Rfam database v12.0 [36,37]. All matches above the curated GA threshold were included. INFERNAL was selected because it implements the CMs that provide the most accurate bioinformatic annotation tool for ncRNAs available [38]. tRNA-scan v.1.3.1 was subsequently used to refine the annotation of tRNA genes (Table S3). The method uses a number of heuristics to increase the search-speed, annotates the Isoacceptor Type of each prediction, infers if predictions are likely to be functional or tRNA-derived pseudogenes [39,40]. This method uses CMs to identify tRNAs. Rfam matches and the tRNA-scan results for families belonging to the same clan were then "competed", so that only the best match was retained for any genomic region [37].

### *Transposable element annotation*

Repetitive elements were identified using a bespoke pipeline. Firstly, elements were identified using RepeatMasker v.4.0.8 [41] with the eukaryota RepBase [42] repeat library. Low-complexity repeats were

ignored (-nolow) and a sensitive (-s) search was performed. Following this, a de novo repeat library was constructed using RepeatModeler v.1.0.11 [43] , including RECON v.1.08 [44] and RepeatScout v.1.0.5 [45]. Novel repeats identified by RepeatModeler were analyzed with a 'BLAST, Extract, Extend' process to characterise elements along their entire length [46]. Consensus sequences and classification information for each repeat family were generated. The resulting de novo repeat library was utilized to identify repetitive elements using RepeatMasker.

## Data analyses

### *Comparative analyses of transposable elements*

We estimated the total repeat content of the giant squid genome to be approximately half its total size (~49.1%) (Figure 1, Supplementary Table S4). Out of all the repeats present in the giant squid genome, only a few were predicted to be small RNAs, satellites, simple or low complexity repeats (~0.89% of the total genome), with the vast majority (~48.21%) instead consisting of Transposable elements (TEs; i.e. SINEs, LINEs, LTR retrotransposons, and DNA transposons; Figure 1, Supplementary Table S4). Of the TE portion of the giant squid genome, the main contribution from annotated TEs is from DNA elements (11.06%) and LINEs (6.96%), with only a small contribution from SINEs (1.99%) and LTR elements (0.72%). TEs are a nearly universal feature of eukaryotic genomes, often comprising a large proportion of the total genomic DNA (e.g. the maize genome is ~85% TEs [47], stick insect genome is ~52% TEs [48], and the human genome is >45% TEs [49]), consequently these account for the majority of observed genome size variation among animals.

In Figure 1, we summarise the recently reported TE analyses performed on assembled cephalopod genomes, as follows: California two-spot octopus (*Octopus bimaculatus*) [11] and long-arm octopus (*O. minor*) [50], Hawaiian bobtail squid (*Euprymna scolopes*) [51], and giant squid (*Architeuthis dux*). The varying sequencing strategies employed to generate currently available cephalopod genomes (and accompanying variation in assembly quality) complicates the comparative analysis of TE content for this group. However, notwithstanding this caveat, it does seem clear that TEs make up a large fraction of the

total genomic content across all cephalopod genomes published to date (Figure 1). DNA transposons and LINEs dominate in available cephalopod genomes, while LTR elements and SINEs generally represent a minor portion of cephalopod TEs (Figure 1). Within decapod cephalopods (i.e. squid and cuttlefish), patterns in TE content are generally similar, however, the giant squid has a notably larger proportion of DNA transposons (1,626,482 elements, 11.06% of the total genome) than the Hawaiian bobtail squid (855,308 elements, 4.05% of the total genome), with the bobtail squid in turn having a similar proportion of LINEs (752,629 elements, 6.83% of the total genome) than the giant squid (766,382 elements, 6.96% of the total genome; Figure 1).

The defining ability of TEs to mobilise, in other words, to transfer copies of themselves into other parts of the genome, can result in harmful mutations. However, TEs can also facilitate the generation of genomic novelty, and there is increasing evidence of their importance for the evolution of host-adaptive processes [52]. In the giant squid genome, all classes of TEs were more frequent (~38.23) in intergenic regions (here defined as regions >2kb upstream or downstream of an annotated gene), than in genic regions versus % of the genome in intergenic regions (~16.6%; Figure 2A). These findings are broadly similar to those reported for other cephalopods, although a larger proportion of the giant squid genome is composed of repeats located within genic regions (percentage of the genome represented by TEs for *O. bimaculoides*: ~6% genic versus ~30% intergenic, and for *O. minor* ~6% genic versus ~40% intergenic [50]).

A Kimura distance-based copy divergence analysis revealed that the most frequent TE sequence divergence relative to the TE consensus sequence in the giant squid genome was ~5-8% across all repeat classes, suggesting a relatively recent transposition burst across all major TE types (Figure 2B). Divergence peaks were most pronounced in LINE RTE elements, Tc/Mar and hAT DNA transposons, and unclassified TEs, with smaller divergence peaks in SINE tRNA elements and Penelope LINE elements (Figure 2B). Divergence peaks were most pronounced in LINE RTE elements, Tc/Mar and hAT DNA

transposons, and unclassified TEs, with smaller divergence peaks in SINE tRNA elements and Penelope LINE elements (Figure 2B). In comparison to observations from other cephalopods, these results suggest a shorter and more intense burst of recent TE activity in the giant squid genome. Overall, further genomic sampling within each of the cephalopod clades will be needed to understand TE evolution, as closely related species can show significant differences (*e.g.*, *O. bimaculoides* to *O. vulgaris*) [53].

### *Non-coding RNAs*

We identified 50,598 ncRNA associated loci in the squid sequencing data, using curated homology-based probabilistic models from the Rfam database[54] and the specialized tRNAscan-SE transfer RNA annotation tool [39]. The essential and well conserved Metazoan ncRNAs: tRNAs, rRNAs (5S, 5.8S, SSU and LSU), RNase P, RNase MRP, SRP and the major spliceosomal snRNAs (U1, U2, U4, U5, U6), as well as the minor spliceosomal snRNAs (U11, U12, U4atac & U6atac), are all found in the *A. dux* genome. Some of the copy numbers associated with the core ncRNAs are extreme. For example, we identified: i) approximately 24,000 loci that appear to derive from 5S rRNA; ii) approximately 17,000 loci that are predicted to be tRNA derived; iii) approximately 3,200 Valine tRNAs isotypes and approximately 1,300 U2 spliceosomal RNAs. The microRNA mir-598 also exhibits high copy-numbers at 172. Many of these are likely to be SINEs derived by transposition. All 20 tRNA isotypes were identified in *A. dux* genome. Again, many of these had relatively large copy numbers (summarised in Table 1). These ranged from 46 (Cys) up to 2,541 (Val). We identified 174 loci that share homology with 34 known snoRNA families, these included 15 scaRNA, 41 H/ACA box and 118 C/D box snoRNA associated loci [10]. The snoRNAs are predominantly involved in rRNA maturation. We identified 7,049 loci that share homology with 283 families of microRNA. Some of these may be of limited reliability, as CMs for simple hairpin structures can also match other, non-homologous, hairpin-like structures in the genome e.g. inverted repeats. A number of cis-regulatory elements were also identified. These included 235 hammerhead 1 ribozymes, 133 Histone 30 UTR stem-loops, and 14 Potassium channel RNA editing signal sequences. There are very

few matches to obvious non-metazoan RNA families in the current assemblies. The only notable exceptions are bablM, IMES-2, PhotoRC-II and rspL. Each of these families are also found in marine metagenomic datasets, possibly explaining their presence as “contamination” from the environment.

### *Analyses of specific gene families*

A number of gene families involved in development, such as transcription factors or signalling ligands, are highly conserved across metazoans and may therefore reveal signatures of genomic events, such as a whole genome duplication.

WNT is a family of secreted lipid-modified signaling glycoproteins with a key role during development [55]. Comparative analysis of molluscan genomes indicates that the ancestral state was 12 *WNT* genes, as *Wnt3* is absent in all protostomes examined thus far [56]. The giant squid has the typical 12 lophotrochozoan WNTs (1, 2, 4, A, 5, 6, 7, 8, 9, 10, 11 and 16; Supplementary Figure S2), and therefore has retained the ancestral molluscan complement, including *Wnt8*, which is absent, for instance, in the genome of the slipper snail *Lottia gigantea* [57].

Protocadherins are a family of cell adhesion molecules that appear to play an important role in vertebrate brain development [58]. It is thought that they act as multimers at the cell surface in a manner akin to DSCAM in flies, which lack protocadherins [59]. Cephalopods have massively expanded this family, with 168 identified in the *O. bimaculoides* genome, whereas only 17-25 protocadherins have been identified in the genomes of annelids and non-cephalopod molluscs [11]. We identified approximately 135 protocadherin genes in *A. dux*, many of which are located in clusters in the genome. The possibility that this gene family plays a developmental role parallel to that of protocadherins in vertebrate neurodevelopment thus remains a compelling hypothesis.

Development organisation of the highly diverse body plans found in the Metazoa is controlled by a conserved cluster of homeotic genes, which includes, among others, the Hox genes. These are

characterized by a DNA sequence referred to as the homeobox, comprising 180 nucleotides that encode the homeodomain [60]. Hox genes are usually found in tight physical clusters in the genome and are sequentially expressed in the same chronological order as they are physically located in the DNA (temporal and spatial collinearity) [61]. Different combinations of Hox gene expression in the same tissue type can lead to a wide variety of different structures [62]. This makes the Hox genes a key subject for understanding the origins of the multitude of forms found in the cephalopods. In *Octopus bimaculoides* genome assembly no scaffold contained more than a single Hox gene, meaning that they are fully atomised [11]. However, in Euprymna scolopes, the Hox cluster was found spanning two scaffolds [51]. In the giant squid, we recovered a full Hox gene cluster in a single scaffold (Figure 3). The Hox gene organization found in the giant squid genome suggests either the presence of a disorganised cluster, so-called type D, or atomised clusters, type A [62], or possibly a combination of the two (the genes are still organized, but physically distant from each other). The existence of a "true" cluster seems unlikely, given the presence of other unrelated genes in between and the relatively large distances. The classification as type D (atomised) might seem most obvious, despite the co-presence of the genes in a single scaffold, due to these large distances. However, the definition of type A (disorganised) does allow for the presence of non-Hox genes in between members of the cluster. Thus, it is difficult to clearly categorise the recovered "cluster", but it does remain clear that these genes are not as tightly bundled as they are in other Bilateria lineages. The *A. dux* Hox “cluster” is spread across 11 Mb of a 38 Mb scaffold, and this suggests a far larger size range in the cephalopods than in other described animals, as recently suggested based on the genome of *Euprymna scolopes* [51]. It is possible that this is the reason for the apparent atomisation of Hox genes in the more fragmented *O. bimaculoides* assembly. Hox clusters are usually found in contigs of around 100 kb length in vertebrates [6, 7] and between 500 - 10,000 kb in invertebrates [8] An assembled contig easily containing the complete cluster for these smaller cluster sizes, would manage to cover only one member of the Hox gene cluster in the studied

coleoids. As such, our results suggest that the Hox cluster may not be fully atomised in *O. bimaculoides* as previously hypothesised. Further improvements of genome assemblies in cephalopods will be required to address this question. The biological reason for this dramatic increase in the distance between the genes in the Hox cluster presents an intriguing avenue of future research. The homeodomain of all the obtained Hox genes in cephalopods were compared with those of other mollusks. Few differences were found relative to a previous study [63], as no significant modifications were observed in Hox 1, Hox 4, ANTP, Lox 2, Lox 5, Post 1 and Post 2. Hox 1 did, however, show reduced conservation in residues 22 to 25 in the *A. dux* sequence. This observation for Hox 1 in *A. dux* is visible only in the Pacbio assembly. Additionally, the Hox 3 homeodomain analysis supports a basal placement of the nautiloids within cephalopods. The Lox 4 gene was the most variable among all groups. As of to date, Hox 2 still remains undetected in the coleoid cephalopods [64]. Assembly errors notwithstanding, gain and loss of Hox genes has been attributed to fundamental changes in animal body plans, and the apparent loss of Hox 2 may therefore be significant. For example, Hox gene loss has been associated with the reduced body-plan segmentation of spider mites [42]. The circumstance that Hox 2 has been readily found in *Nautilus*, but remains undetected in all coleoids sequenced thus far, might signify an important developmental split within the Cephalopoda. Alternatively, and equally intriguing, this Hox gene may have undergone such drastic evolutionary modifications that it is presently undetectable by conventional means.

On a final note, we analyzed genes encoding reflectins, a class of cephalopod-specific proteins first described in *E. scolopes* [65]. Reflectins form flat structures that reflect ambient light (other marine animals use purine-based platelets), thus modulating iridescence for communication or camouflage purposes [66]. The giant squid genome contains 7 reflectin genes and 3 reflectin-like genes (Supplementary Figure S3). All of these genes, with the exception of 1 reflectin gene, appear on the

same scaffold, which corresponds very well with the distribution pattern of octopus reflectin genes [11]).

## Conclusions

Not only because of its astonishing proportions, but also for the lack of knowledge of the key facets of its deep-sea lifestyle, the giant squid has long captured the imagination of scientists and the general public alike. With the release of this annotated giant squid genome, we set the stage for future research into the enigmas that enshroud this truly awe-inspiring creature. Further, given the paucity of available cephalopod genomes, we provide a valuable contribution to the genomic description of cephalopods, and more widely to the growing number of fields that are recognizing the potential, which this group of behaviourally advanced invertebrates holds for improving our understanding of the diversity of life on Earth in general.

## Availability of supporting data

The data sets supporting the results of this article are available in the NCBI database a Bioproject PRJNA534469. The three transcriptome data sets (tsa) have ids GHKK01000000, GHKL01000000 and GHKH01000000 and the sequence data used for the genome assemblies has id VCCN01000000.

## Additional files

Supplement.txt. Supplementary methods, tables and figures.

# Declarations

## Abbreviations

Gb: gigabase pairs; Mb: megabase pairs; BUSCO: Benchmarking Universal Single-copy Orthologs; bp: base pair; NCBI: National Center for Biotechnology Information; LC-MS/MS: liquid chromatography (LC) tandem mass spectrometry (MS); CCB: Colloidal Coomassie Brilliant Blue; HCD: higher-energy collisional

dissociation; NCE: normalized collisional energy; AGC: Automatic Gain Control; ALC: Average Local Confidence; SP: SwissProt; Uf: UniRef90; CDD: conserved domain database; CM: covariance model; TE: transposable element; LINE: Long interspersed nuclear element; SINE: Short interspersed nuclear element; LRT: long terminal repeat.

## Ethics statement

Sampling was following the recommendations from Moltschaniwskyj et al., 2007 [24].

## Consent for publication

Not applicable.

## Competing interests

The authors declare that they have no competing interests.

## Funding

R.R.F. thanks the Villum Fonden for grant VKR023446 (Villum Fonden Young Investigator Grant), the Portuguese Science Foundation (FCT) for grant PTDC/MAR/115347/2009;COMPETE-FCOMP-01-012; FEDER-015453, Marie Curie Actions (FP7-PEOPLE-2010-IEF, Proposal 272927), and the Danish National Research Foundation (DNRF96) for its funding of the Center for Macroecology, Evolution, and Climate. H.O. thanks the Rede Nacional de Espectrometria de Massa, ROTEIRO/0028/2013, ref. LISBOA-01-0145-FEDER-022125, supported by COMPETE and North Portugal Regional Operational Programme (Norte2020), under the PORTUGAL 2020 Partnership Agreement, through the European Regional Development Fund (ERDF). A.C. thanks FCT for project UID/Multi/04423/2019. M.P. acknowledges the support from the Wellcome Trust (grant number WT108749/Z/15/Z) and the European Molecular Biology Laboratory. M.P.T.G. thanks the Danish National Research Foundation for its funding of the Center for GeoGenetics (grant DNRF94) and Lundbeck Foundation for grant R52-5062 on Pathogen

Palaeogenomics. S.R. was supported by the Novo Nordisk Foundation grant NNF14CC0001. A.H. is supported by a Biotechnology and Biological Sciences Research Council David Phillips Fellowship [fellowship reference: BB/N020146/1]. T.B. is supported by the Biotechnology and Biological Sciences Research Council-funded South West Biosciences Doctoral Training Partnership [training grant reference BB/M009122/1]. This work was partially funded by the Lundbeck Foundation (R52-A4895 to BB).

HJTH was supported by the David and Lucile Packard Foundation, the Netherlands Organization for Scientific Research (#825.09.016) and currently by the Deutsche Forschungsgemeinschaft (DFG) under grant HO 5569/1-2 (Emmy Noether Junior Research Group). T.V. and B.Br. were supported by grants from the Slovak grant agency VEGA (1/0684/16, 1/0458/18). Computation for the work described in this paper was partially supported by the DeiC National Life Science Supercomputer at DTU.

## Authors contributions

R.D.F. and M.T.P.G. designed the study. J.S., H-J.H. AND R.T. carried out the sampling. Alex.C., A.R., B.F., G.C.A.Jr, H.O. and I.W. performed the laboratory work. R.D.F., Alv.C., A.M., C.B.A., F.S., P.G., T.B., A.H., I.B.H., C.C., B.P., F.P., M.P., F.M., O.S., S.R., M.Z.R. and D.P. analyzed the data. E.J., G.Z., J.V., O.F. and Q.L. contributed with genomic resources. R.D.F., L.F.C.C., A.A., Y.W., B.M., R.S., T.V., B.B., T.S-P., M.T.P.G. contributed with supervision and computational resources. R.R.F., T.S-P., R.N., M.T.P.G paid for sequencing. R.D.F. wrote the manuscript with contributions from all authors. All authors have read and approved the manuscript.

## Acknowledgments

We would like to thank: Anders Hansen, Tobias Mourier, Kristin Rós Kjartansdóttir and Lars Hestbjerg Hansen for help with generating sequencing data; Shawn Hoon for sharing transcriptome data; Annie Lingren for help with sample shipping; Peter Smith for providing samples and the support of the entire team at Dovetail.

## References

1. Zullo L, Hochner B. A new perspective on the organization of an invertebrate brain. Commun. Integr. Biol. 2011;4:26–9.

2. Nixon M, Young JZ. The brains and lives of cephalopods. Oxford: Oxford University Press, Oxford; 2003.

3. Doubleday ZA, Prowse TAA, Arkhipkin A, Pierce GJ, Semmens J, Steer M, et al. Global proliferation of cephalopods. Curr. Biol. 2016;26:R406–7.

4. Vecchione M, Allcock L, Piatkowski U, Jorgensen E, Barratt I. Persistent Elevated Abundance of Octopods in an Overfished Antarctic Area. Smithson. Poles Contrib. to Int. Polar Year Sci. 2009:197–204.

5. Young RE, Vecchione M, Mangold KM. Cephalopoda, Cuvier 1797. Tree Life. 2018. Available from: http://tolweb.org/Cephalopoda/19386

6. Roper CF, Sweeney MJ, Nauen CE. FAO Species Catalogue Vol. 3. Cephalopods of the world. An annotated and illustrated catalogue of species of interest to fisheries. FAO Fish. Synopsis. 1984;125:277.

7. Jereb P, Roper CFE. Cephalopods of the world. An annotated and illustrated catalogue of cephalopod species known to date. Myopsid and Oegopsid Squids. FAO Species Cat. Fish. Purp. 2010;2:605.

8. McClain CR, Balk MA, Benfield MC, Branch TA, Chen C, Cosgrove J, et al. Sizing ocean giants: patterns of intraspecific size variation in marine megafauna. PeerJ. 2015;3:e715.

9. Paxton CGM. Unleashing the Kraken: on the maximum length in giant squid (Architeuthis sp.). J. Zool. 2016;300:82–8.

10. Rosa R, Seibel BA. Slow pace of life of the Antarctic colossal squid. J. Mar. Biol. Assoc. United Kingdom. 2010;90:1375–8.

11. Albertin CB, Simakov O, Mitros T, Wang ZY, Pungor JR, Edsinger-Gonzales E, et al. The octopus genome and the evolution of cephalopod neural and morphological novelties. Nature. 2015;524:220–4.

12. Liscovitch-Brauer N, Alon S, Porath HT, Elstein B, Unger R, Ziv T, et al. Trade-off between Transcriptome Plasticity and Genome Evolution in Cephalopods. Cell. 2017;169:191-202.e11.

13. Gilly WF, Beman JM, Litvin SY, Robison BH. Oceanographic and Biological Effects of Shoaling of the Oxygen Minimum Zone. Ann. Rev. Mar. Sci. 2013;5:393–420.

14. Golikov AV, Sabirov RM, Lubin PA, Jørgensen LL. Changes in distribution and range structure of Arctic cephalopods due to climatic changes of the last decades. Biodiversity. 2013;14:28–35.

15. Balmaseda MA, Trenberth KE, Källén E. Distinctive climate signals in reanalysis of global ocean heat content. Geophys. Res. Lett. 2013;40:1754–9.

16. Freeman D, Marshall B, Ahyong S, Wing S, Hitchmough R. Conservation status of New Zealand marine invertebrates, 2009. New Zeal. J. Mar. Freshw. Res. 2010;44:129–48.

17. Bustamante P, González AF, Rocha F, Miramand P, Guerra A. Metal and metalloid concentrations in the giant squid Architeuthis dux from Iberian waters. Mar. Environ. Res. 2008;66:278–87.

18. Unger MA, Harvey E, Vadas GG, Vecchione M. Persistent pollutants in nine species of deep-sea cephalopods. Mar. Pollut. Bull. 2008;56:1498–500.

19. Winkelmann I, Campos PF, Strugnell J, Cherel Y, Smith PJ, Kubodera T, et al. Mitochondrial genome diversity and population structure of the giant squid Architeuthis: genetics sheds new light on one of the most enigmatic marine species. Proceedings. Biol. Sci. 2013;280:20130273.

20. Chikhi R, Medvedev P. Informed and automated k-mer size selection for genome assembly. Bioinformatics. 2014;30:31–7.

21. Chapman J a, Ho I, Sunkara S, Luo S, Schroth GP, Rokhsar DS. Meraculous: de novo genome assembly with short paired-end reads. PLoS One. 2011;6:e23501.

22. Simão FA, Waterhouse RM, Ioannidis P, Kriventseva E V., Zdobnov EM. BUSCO: assessing genome assembly and annotation completeness with single-copy orthologs. Bioinformatics. 2015;31:3210–2.

23. English AC, Richards S, Han Y, Wang M, Vee V, Qu J, et al. Mind the Gap: Upgrading Genomes with Pacific Biosciences RS Long-Read Sequencing Technology. Liu Z, editor. PLoS One. 2012;7:e47768.

24. Moltschaniwskyj NA, Hall K, Lipinski MR, Marian JEAR, Nishiguchi M, Sakai M, et al. Ethical and welfare considerations when using cephalopods as experimental animals. Rev. Fish Biol. Fish. 2007;17:455–76.

25. Patel RK, Jain M. NGS QC Toolkit: A Toolkit for Quality Control of Next Generation Sequencing Data. Liu Z, editor. PLoS One. 2012;7:e30619.

26. Martin M. Cutadapt removes adapter sequences from high-throughput sequencing reads. EMBnet.journal. 2011;17:10–2.

27. Haas BJ, Papanicolaou A, Yassour M, Grabherr M, Blood PD, Bowden J, et al. De novo transcript sequence reconstruction from RNA-seq using the Trinity platform for reference generation and analysis. Nat. Protoc. 2013;8:1494–512.

28. Gilbert D. Gene-omes built from mRNA seq not genome DNA. Notre Dame: 7th annual arthropod genomics symposium; 2013. Available from: http://globalhealth.nd.edu/7th-annual-arthropod-genomics-symposium/

29. Kleffmann T, Russenberger D, von Zychlinski A, Christopher W, Sjölander K, Gruissem W, et al. The Arabidopsis thaliana chloroplast proteome reveals pathway abundance and novel protein functions. Curr. Biol. 2004;14:354–62.

30. Bradford MM. A rapid and sensitive method for the quantitation of microgram quantities of protein utilizing the principle of protein-dye binding. Anal. Biochem. 1976;72:248–54.

31. Santos R, da Costa G, Franco C, Gomes-Alves P, Flammang P, Coelho A V. First Insights into the Biochemistry of Tube Foot Adhesive from the Sea Urchin Paracentrotus lividus (Echinoidea, Echinodermata). Mar. Biotechnol. 2009;11:686–98.

32. Neuhoff V, Arold N, Taube D, Ehrhardt W. Improved staining of proteins in polyacrylamide gels including isoelectric focusing gels with clear background at nanogram sensitivity using Coomassie Brilliant Blue G-250 and R-250. Electrophoresis. 1988;9:255–62.

33. Brejová B, Vinar T, Chen Y, Wang S, Zhao G, Brown DG, et al. Finding genes in Schistosoma japonicum: annotating novel genomes with help of extrinsic evidence. Nucleic Acids Res. 2009;37:e52.

34. Musacchia F, Basu S, Petrosino G, Salvemini M, Sanges R. Annocript: a flexible pipeline for the annotation of transcriptomes able to identify putative long noncoding RNAs. Bioinformatics. 2015;31:2199–201.

35. Camacho C, Coulouris G, Avagyan V, Ma N, Papadopoulos J, Bealer K, et al. BLAST+: architecture and applications. BMC Bioinformatics. 2009;10:421.

36. Burge SW, Daub J, Eberhardt R, Tate J, Barquist L, Nawrocki EP, et al. Rfam 11.0: 10 years of RNA families. Nucleic Acids Res. 2013;41:D226–32.

37. Gardner PP, Daub J, Tate J, Moore BL, Osuch IH, Griffiths-Jones S, et al. Rfam: Wikipedia, clans and the "decimal" release. Nucleic Acids Res. 2011;39:D141–5.

38. Freyhult EK, Bollback JP, Gardner PP. Exploring genomic dark matter: a critical assessment of the performance of homology search methods on noncoding RNA. Genome Res. 2007;17:117–25.

39. Chan PP, Lowe TM. GtRNAdb: a database of transfer RNA genes detected in genomic sequence. Nucleic Acids Res. 2009;37:D93–7.

40. Lowe TM, Eddy SR. tRNAscan-SE: a program for improved detection of transfer RNA genes in genomic sequence. Nucleic Acids Res. 1997;25:955–64.

41. Smit AFA, Hubley RR, Green PR. RepeatMasker Open-4.0. 2013.

42. Bao W, Kojima KK, Kohany O. Repbase Update, a database of repetitive elements in eukaryotic genomes. Mob. DNA. 2015;6:11.

43. Smit A, Hubley R. RepeatModeler Open-1.0. 2015.

44. Bao Z, Eddy SR. Automated de novo identification of repeat sequence families in sequenced genomes. Genome Res. 2002;12:1269–76.

45. Price AL, Jones NC, Pevzner PA. De novo identification of repeat families in large genomes. Bioinformatics. 2005;21:i351–8.

46. Platt RN, Blanco-Berdugo L, Ray DA. Accurate Transposable Element Annotation Is Vital When Analyzing New Genome Assemblies. Genome Biol. Evol. 2016;8:403–10.

47. Schnable PS, Ware D, Fulton RS, Stein JC, Wei F, Pasternak S, et al. The B73 Maize Genome: Complexity, Diversity, and Dynamics. Science. 2009;326:1112–5.

48. Wu C, Twort VG, Crowhurst RN, Newcomb RD, Buckley TR. Assembling large genomes: analysis of the stick insect (Clitarchus hookeri) genome reveals a high repeat content and sex-biased genes associated with reproduction. BMC Genomics. 2017;18:884.

49. Initial sequencing and analysis of the human genome. Nature. 2001;409:860–921.

50. Kim B-M, Kang S, Ahn D-H, Jung S-H, Rhee H, Yoo JS, et al. The genome of common long-arm octopus

Octopus minor. Gigascience. 2018;7.

51. Belcaid M, Casaburi G, McAnulty SJ, Schmidbaur H, Suria AM, Moriano-Gutierrez S, et al. Symbiotic organs shaped by distinct modes of genome evolution in cephalopods. Proc. Natl. Acad. Sci. U. S. A. 2019;116:3030–5.

52. Schrader L, Schmitz J. The impact of transposable elements in adaptive evolution. Mol. Ecol. 2018;28:1537-1549.

53. Zarrella I, Herten K, Maes GE, Tai S, Yang M, Seuntjens E, et al. The survey and reference assisted assembly of the Octopus vulgaris genome. Sci. Data. 2019;6:13.

54. Nawrocki EP, Burge SW, Bateman A, Daub J, Eberhardt RY, Eddy SR, et al. Rfam 12.0: updates to the RNA families database. Nucleic Acids Res. 2015;43:D130–7.

55. Cadigan KM, Nusse R. Wnt signaling: a common theme in animal development. Genes Dev. 1997;11:3286–305.

56. Cho S-J, Valles Y, Giani VC, Seaver EC, Weisblat DA. Evolutionary Dynamics of the wnt Gene Family: A Lophotrochozoan Perspective. Mol. Biol. Evol. 2010;27:1645–58.

57. Simakov O, Marletaz F, Cho S-J, Edsinger-Gonzales E, Havlak P, Hellsten U, et al. Insights into bilaterian evolution from three spiralian genomes. Nature. 2012;493:526–31.

58. Chen W V, Maniatis T. Clustered protocadherins. Development. 2013;140:3297–302.

59. Zipursky SL, Sanes JR. Chemoaffinity revisited: dscams, protocadherins, and neural circuit assembly. Cell. 2010;143:343–53.

60. Pratihar S, Prasad Nath R, Kumar Kundu J. Hox genes and its role in animal development. Int. J. Science and Nature. 2010;1:101-103

61. Fröbius AC, Matus DQ, Seaver EC. Genomic Organization and Expression Demonstrate Spatial and Temporal Hox Gene Colinearity in the Lophotrochozoan Capitella sp. I. Butler G, editor. PLoS One. 2008;3:e4004.

62. Mallo M, Wellik DM, Deschamps J. Hox genes and regional patterning of the vertebrate body plan. Dev. Biol. 2010;344:7–15.

63. Pernice M, Deutsch JS, Andouche A, Boucher-Rodoni R, Bonnaud L. Unexpected variation of Hox genes' homeodomains in cephalopods. Mol. Phylogenet. Evol. 2006;40:872–9.

64. Barucca M, Canapa A, Biscotti MA, Zappavigna V. An Overview of Hox Genes in Lophotrochozoa: Evolution and Functionality. J. Dev. Biol. 2016;4:1-15.

65. Crookes WJ, Ding L-L, Huang QL, Kimbell JR, Horwitz J, McFall-Ngai MJ. Reflectins: The Unusual Proteins of Squid Reflective Tissues. Science. 2004;303:235–8.

66. Wardill TJ, Gonzalez-Bellido PT, Crook RJ, Hanlon RT. Neural control of tuneable skin iridescence in squid. Proc. R. Soc. B Biol. Sci. 2012;279:4243–52.

## Tables

**Table 1.** Statistics of the genome assembly, gene prediction and functional annotation of giant squid. The transcript evidence was confirmed by blastp hits with e-value < $10E^{-6}$ using the transcriptomes of three other species of squid (see the "Transcriptome sequencing" section).

| Global Statistics | Meraculous + Dovetail |
|---|---|
| **Genome assembly*** | |
| Input assembly | Meraculous |
| Contig N50 length (Mb) | 0.005 |
| Longest contig (Mb) | 0.120 |
| Scaffold N50 length (Mb) | 4.852 |
| Longest scaffold (Mb) | 32.889 |
| Total length (Gb) | 2.693 |
| **Busco statistics ([1]Euk / [2]Met)** | |
| Complete BUSCOs, (%) | 86.1 / 88.5 |
| Complete and single-copy, (%) | 85.1 / 87.6 |
| Complete and duplicated, (%) | 1.0 / 0.9 |
| Partial, (%) | 4.3 / 3.6 |
| Missing, (%) | 9.6 / 7.9 |
| Total Buscos found, (%) | 90.4 / 92.1 |
| **Genome annotation / Gene Prediction** | |
| Protein-coding gene number | 51,225 |
| Transcript evidence | 30,472 |
| Average Protein length, (aa) | 253 |
| Longest Protein, (aa) | 17,047 |
| Average CDS length, (bp) | 758 |
| Longest CDS, (bp) | 51,138 |
| Average exon length, (bp) | 186 |

| | |
|---|---|
| Average exons per gene | 4 |
| **Functional annotation (Number of Hits)** | |
| Swissprot | 15,749 |
| Uniref90 | 29,553 |
| GO Terms | 4,712 |
| Conserved Domains Database (CDD) | 15,280 |

*****The presented statistics are to contigs/scaffolds with length >= 500 bp.

[1]Euk: Database of Eukaryota orthologs genes, containing a total of 303 BUSCO groups.

[2]Met: Database of Metazoa orthologs genes, containing a total of 978 BUSCO groups.

## Figure legends

**Figure 1.** Comparison of genome repeat content among available cephalopod genomes with assembled genomes (repeat data for *O. minor* and *O. bimaculoides* from [50] and for *E. scolopes* from [51]). The tree indicates evolutionary relationships among the two available octopod cephalopods and the two available decapod cephalopods. Pie charts are scaled according to genome size (*O. bimaculoides*: 2.7Gb, *O. minor*: 5.09Gb, *E. scolopes*: 5.1Gb, *Architeuthis dux*: 2.7Gb), with different repeat types indicated by the colours presented in the key.

**Figure 2. A)** Stacked bar chart illustrating the proportions (expressed as percentage of the total genome) of repeats found in genic (≤2kb from an annotated gene) and intergenic regions (>2kb from an annotated gene) for the giant squid genome. **B)** Transposable element (TE) accumulation history in the giant squid genome, based on a Kimura distance-based copy divergence analysis of TEs, with Kimura substitution level (CpG adjusted) illustrated on the x-axis, and percentage of the genome represented by each repeat type on the y-axis. Repeat type is indicated by the colour chart below the x-axis.

**Figure 3.** Schematic representation of the Hox gene cluster chromosomal organization in various invertebrates. Different scaffolds are separated by two slashes. Scaffold length is shown underneath. Unlike in other coleoids, for *Architeuthis dux* all Hox genes were found in the same scaffold. However, the distance between the genes was larger than expected for invertebrate organisms, and non-homeobox genes were also present within the cluster. Hox 2 remains undetected in coleoids.

Click here to download Figure Figure_1.pdf

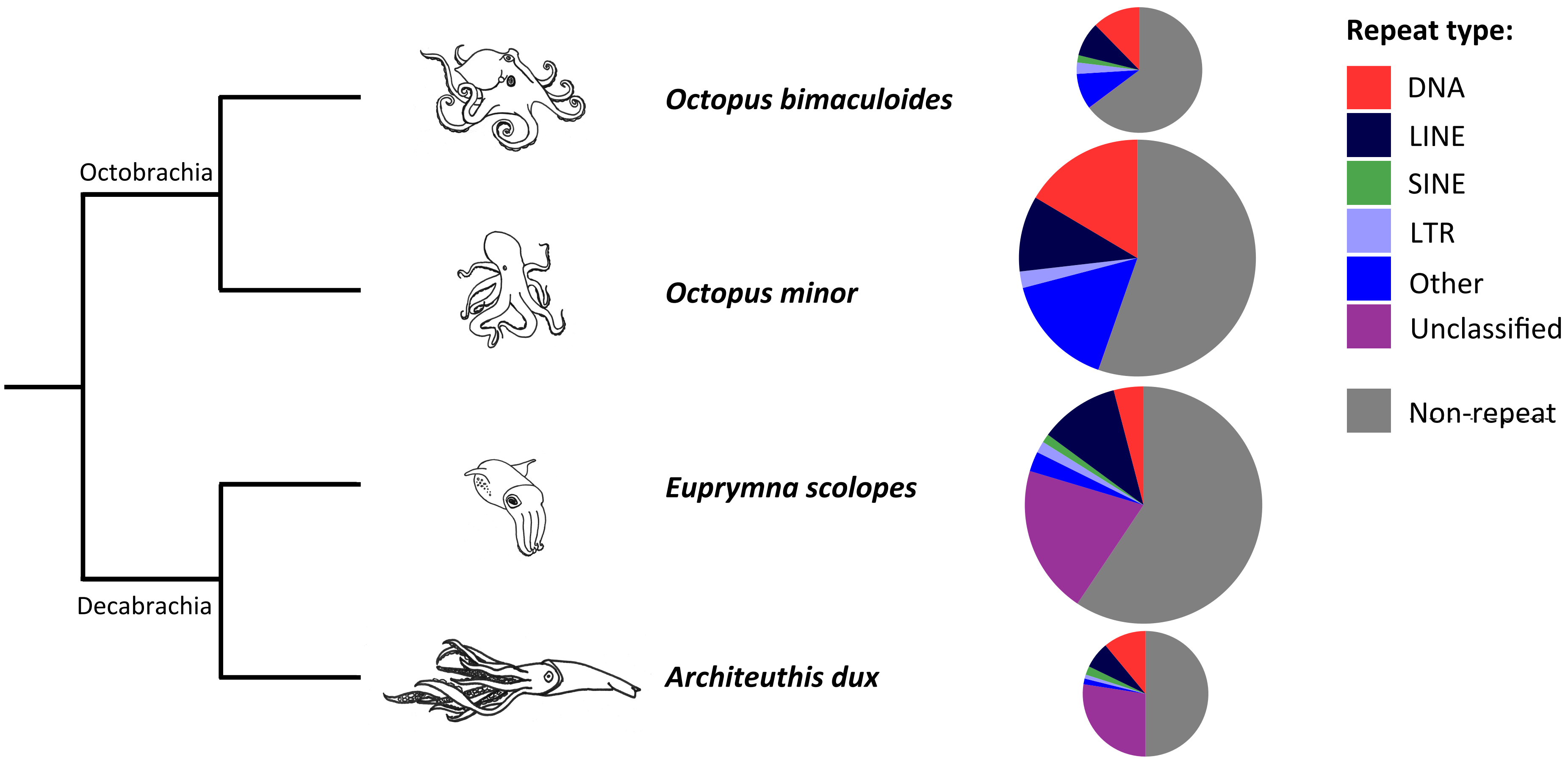

Figure 2

Click here to download Figure Figure_2.pdf

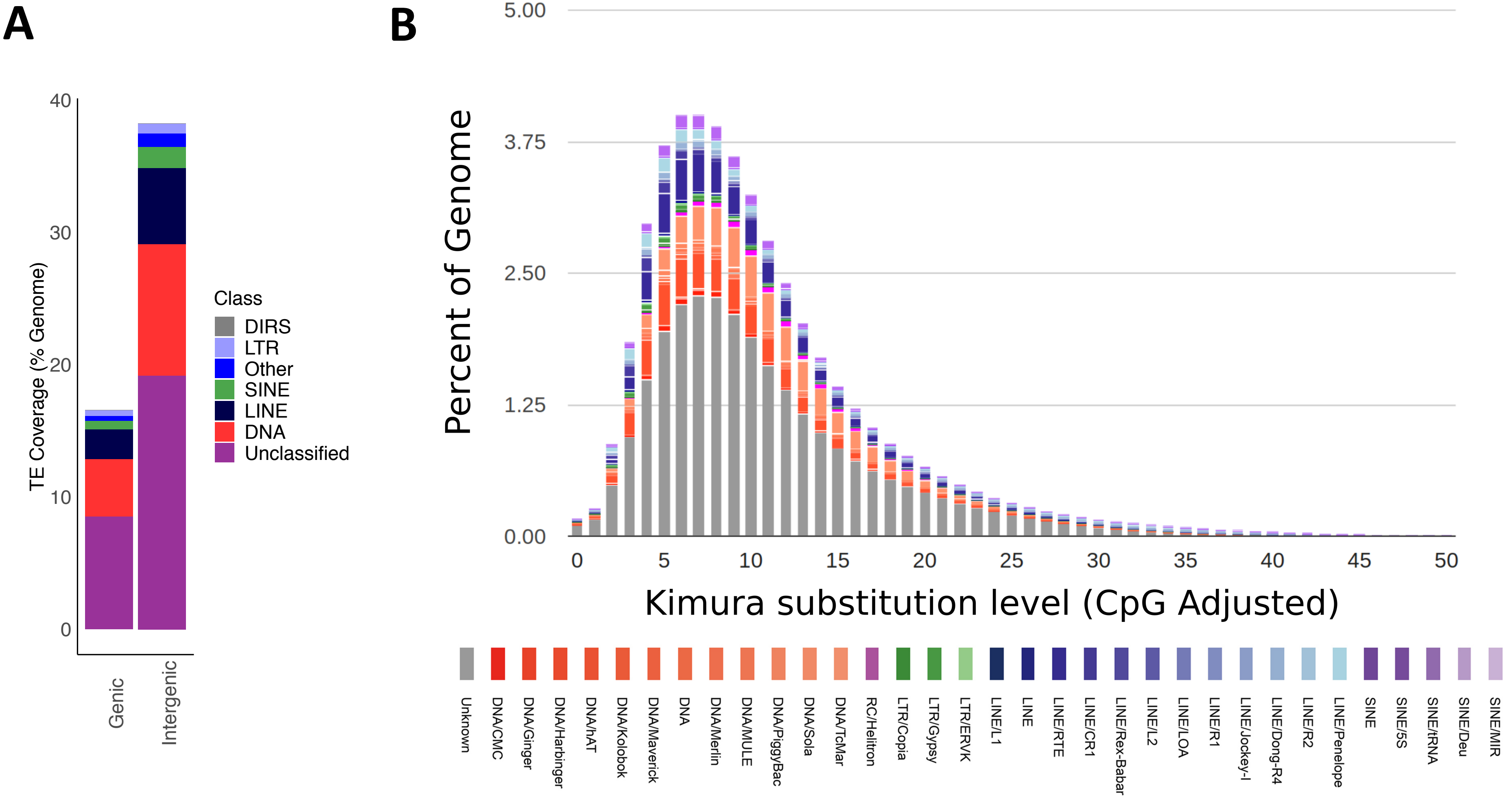

Figure 3 Click here to download Figure Figure_3.pdf

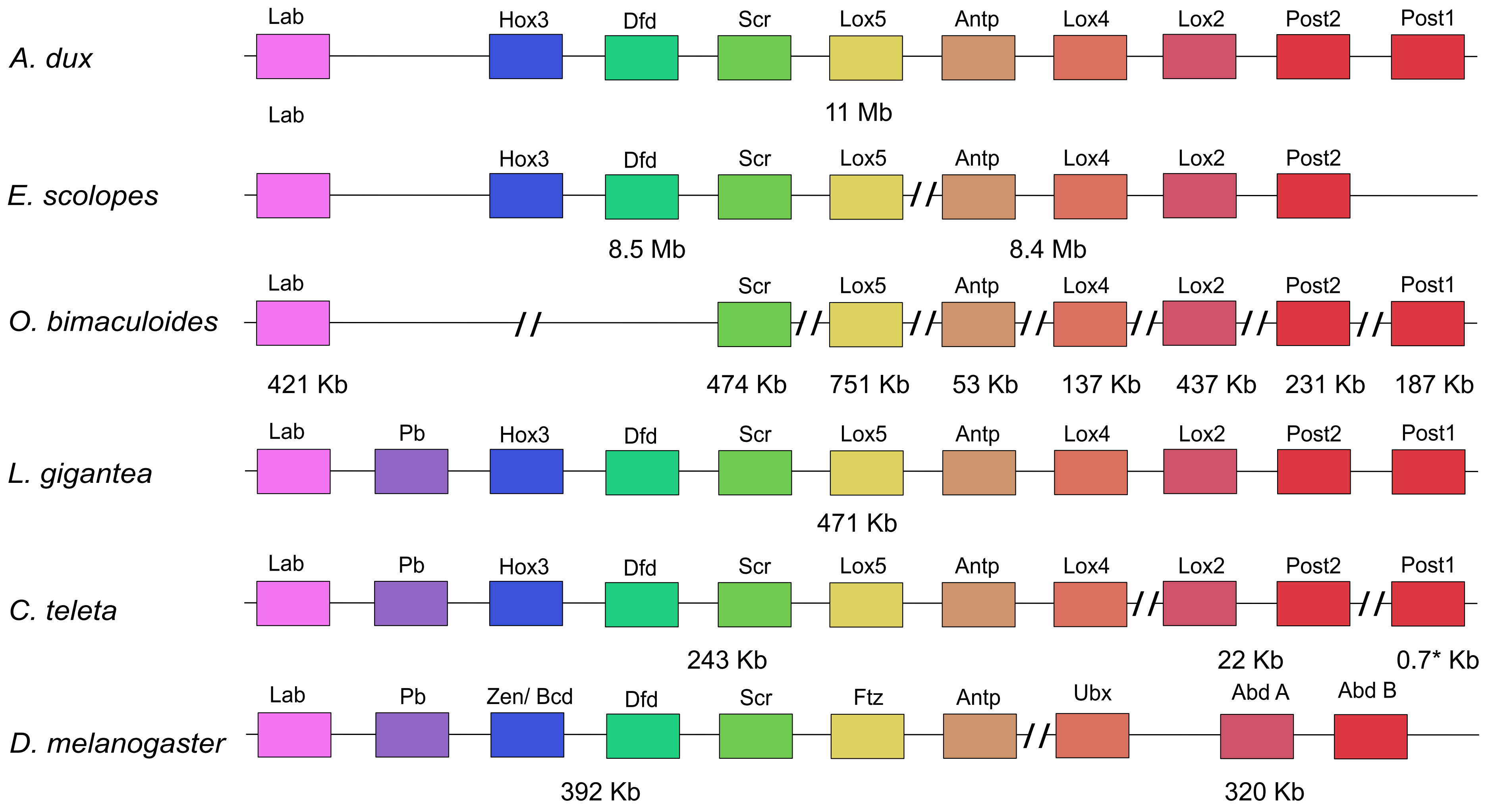


*Gene size only.

Click here to access/download
**Supplementary Material**
RFonseca_supplement.docx

Dear Editor,

We herewith submit our manuscript ‘A draft genome sequence of the elusive giant squid, *Architeuthis dux*’ as Data Note for your formal consideration as a publication in GigaScience.

We present a draft genome assembly with a scaffold N50 of 4.8 Mb (estimated genome size of 2.7 Gb) produced using Illumina, Moleculo and Chicago libraries. We also provide the corresponding gene, RNAs and transposable element annotations, as well as the results of a comparative genomics analyses with other available cephalopod genomes.

Besides providing the community with an important resource for further studying this enigmatic animal, given the paucity of available cephalopod genomes, this is a valuable contribution to the genomic description of cephalopods, and therefore we believe it has the potential to be published in GigaScience.

The sequence data and annotations have been submitted to the NCBI database as Bioproject PRJNA534469, which will be made available upon request from your journal.

We have no competing interests and all authors have approved the manuscript for submission.

We look forward to your assessment.

Best wishes,

Rute Fonseca on behalf of all the authors
