# Supplementary material for: A draft genome sequence of the elusive giant squid, Architeuthis dux
Source: Gigascience. 2020 Jan 16;9(1):giz152. doi: 10.1093/gigascience/giz152 (PMC6962438; doi:10.1093/gigascience/giz152)
Supplement: giz152_GIGA-D-19-00236_Revision_2 [file giz152_giga-d-19-00236_revision_2.pdf]

GigaScience

A draft genome sequence of the elusive giant squid, Architeuthis dux

--Manuscript Draft--

**Manuscript Number:**	GIGA-D-19-00236R2
**Full Title:**	A draft genome sequence of the elusive giant squid, Architeuthis dux
**Article Type:**	Data Note

Funding Information:

Villum Fonden (VKR023446)	Dr Rute R. da Fonseca
FP7 People: Marie-Curie Actions (272927)	Dr Rute R. da Fonseca
Fundação para a Ciência e a Tecnologia (PTDC/MAR/115347/2009)	Dr Rute R. da Fonseca
Danmarks Grundforskningsfond (DNRF96)	Dr Rute R. da Fonseca
Programa Operacional Temático Factores de Competitividade (PT) (COMPETE-FCOMP-01-012)	Dr Rute R. da Fonseca
Rede Nacional de Espectrometria de Massa (ROTEIRO/0028/2013)	Dr Hugo Osório
Fundação para a Ciência e a Tecnologia (UID/Multi/04423/2019)	Dr Alexandre Campos
Wellcome Trust (WT108749/Z/15/Z)	Dr Mateus Patricio
Danmarks Grundforskningsfond (DNRF94)	Dr M. Thomas P. Gilbert
Lundbeckfonden (R52-5062)	Dr M. Thomas P. Gilbert
Novo Nordisk Fonden (NNF14CC0001)	Dr Simon Rasmussen
Biotechnology and Biological Sciences Research Council (BB/N020146/1)	Dr Alex Hayward
Biotechnology and Biological Sciences Research Council (BB/M009122/1)	Dr Tobias Baril
Lundbeckfonden (R52-A4895)	Dr Blagoy Blagoev
Nederlandse Organisatie voor Wetenschappelijk Onderzoek (NL) (#825.09.016)	Dr Henk-Jan Hoving
Deutsche Forschungsgemeinschaft (DE) (HO 5569/2-1)	Dr Rute R. da Fonseca
Slovak grant agency VEGA (VEGA 1/0684/16)	Dr Brona Brejova
Slovak grant agency VEGA (VEGA 1/0458/18)	Dr Tomas Vinar

Abstract:

Background

The giant squid (Architeuthis dux ; Steenstrup, 1857) is an enigmatic giant mollusk with a circumglobal distribution in the deep ocean, except in the high Arctic and Antarctic waters. The elusiveness of the species makes it difficult to study. Thus, having a genome assembled for this deep-sea dwelling species will allow unlocking several pending evolutionary questions.Findings

We present a draft genome assembly that includes 200 Gb of Illumina reads, 4 Gb of Moleculo synthetic long-reads and 108 Gb of Chicago libraries, with a final size matching the estimated genome size of 2.7 Gb, and a scaffold N50 of 4.8 Mb. We also present an alternative assembly including 27 Gb raw reads generated using the Pacific Biosciences platform. In addition, we sequenced the proteome of the same individual

Powered by Editorial Manager® and ProduXion Manager® from Aries Systems Corporation

	and RNA from three different tissue types from three other species of squid species (Onychoteuthis banksii , Dosidicus gigas , and Sthenoteuthis oualaniensis) to assist genome annotation. We annotated 33,406 protein coding genes supported by evidence and the genome completeness estimated by BUSCO reached 92%. Repetitive regions cover 49.17% of the genome.Conclusions<br><br>This annotated draft genome of A. dux provides a critical resource to investigate the unique traits of this species, including its gigantism and key adaptations to deep-sea environments.
**Corresponding Author:**	Rute R. da Fonseca<br>University of Copenhagen<br>Copenhagen, DENMARK
**Corresponding Author Secondary Information:**	
**Corresponding Author's Institution:**	University of Copenhagen
**Corresponding Author's Secondary Institution:**	
**First Author:**	Rute R. da Fonseca
**First Author Secondary Information:**	
**Order of Authors:**	Rute R. da Fonseca
	Alvarina Couto
	Andre Machado
	Brona Brejova
	Caroline B. Albertin
	Filipe Silva
	Paul Gardner
	Tobias Baril
	Alex Hayward
	Alexandre Campos
	Angela Ribeiro
	Inigo Barrio Hernandez
	Henk-Jan Hoving
	Ricardo Tafur-Jimenez
	Chong Chu
	Barbara Frazão
	Bent Petersen
	Fernando Peñaloza
	Francesco Musacchia
	Graham C. Alexander Jr.
	Hugo Osório
	Inger Winkelmann
	Oleg Simakov
	Simon Rasmussen
	M. Ziaur Rahman

Powered by Editorial Manager® and ProduXion Manager® from Aries Systems Corporation

	Davide Pisani
	Erich Jarvis
	Guojie Zhang
	Jakob Vinther
	Jan Strugnell
	L. Filipe C. Castro
	Olivier Fedrigo
	Mateus Patricio
	Qiye Li
	Sara Rocha
	Agostinho Antunes
	Yufeng Wu
	Bin Ma
	Remo Sanges
	Tomas Vinar
	Blagoy Blagoev
	Thomas Sicheritz-Ponten
	Rasmus Nielsen
	M. Thomas P. Gilbert
**Order of Authors Secondary Information:**	
**Response to Reviewers:**	Dear Editor,<br><br>We herewith submit our revised manuscript 'A draft genome sequence of the elusive giant squid, Architeuthis dux'. We have edited the manuscript to clarify the issues raised by you and Reviewer #2, uploaded the files with the filtered annotations to the GigaScience server and updated the README file accordingly. Please find the answers to all comments below.<br><br>Best regards,<br>Rute Fonseca on behalf of all the authors.<br><br>####################<br><br>Editor's comments:<br><br>Reviewer 2 is still concerned regarding the uncertainty of the gene models and says that, ideally, transcriptome data should be used to address this. The reviewer and I are aware that this may not be possible in this species. In this case, I agree with the reviewer that a good way forward would be to provide both, the original and the filtered versions, and discuss the uncertainties around the gene models in the paper.<br><br>We now make it clearer that transcriptomes of closely related squid species were used to guide the annotation process (since it is impossible to get that type of data from a giant squid). We also provide the two sets of annotations and extended our discussion regarding the gene models in the main text (added information from Lines 252 to 278).<br><br>####################<br><br>Reviewer reports:<br>Reviewer #2: The authors have addressed most of my comments. However, I am still cautious about their gene model prediction. Running gene prediction using parameters from other species, especially Drosophila usually gives rise to very inaccurate results.

Powered by Editorial Manager® and ProduXion Manager® from Aries Systems Corporation

	The best situation would be using the transcriptome from the same species to train the gene model predictor. I understand there might be a technical limitation, but applying a random filter threshold to reduce the numbers of gene models is also problematic. This filtering may remove lineage-specific genes (i.e., novel genes in this species) and neural peptide genes that are usually very short. If having a good gene model is not possible, I would recommend the authors providing both versions of their gene models (i.e., original and filtered). And the authors should address this weakness in their manuscript.<br><br>Please note that the model parameters that were used for the final gene prediction were A. dux specific, they were definitely not D. melanogaster parameters. D. melanogaster parameters were used only as a starting point in the iterative process that has been guided, among other things, by RNA-seqs and proteomes from closely-related oegopsid squid species (unfortunately, we cannot obtain RNA-seq from A. dux due to difficulties of obtaining RNA from long-dead specimens). The RNA-seq and proteome information has also been used in the final stage of gene predictions. In this setup, the gene finder can adapt to new species (even species distant from the original parameters) and can give predictions that is in high concordance with related RNA-seq / proteome information where such information is available, while still predicting novel genes in the areas not covered by such evidence.<br><br>Methodology of iterative adaptation of gene finding parameters to new species has been previously rigorously evaluated by us (see reference [32] in the paper) as well as others (see e.g. Korf 2004, Lomsadze et al. 2005) and has been confirmed to lead to fast adaptation of the parameters to new species. We have made additional changes to the text describing gene finding to make this more apparent.<br><br>As to the high number of gene predictions, we think that this is mostly artefact of low contiguity of the assembly (lots of sequencing gaps) that leads to shorter gene models. (This issue is already discussed in the paper.) You are, of course, correct in pointing out that filtering for “supported” genes may lead to exclusion of truly novel genes. Based on your suggestion, we now provide both original and filtered data sets of gene models.<br><br>We base the downstream functional analysis on the filtered gene set, which is done based on sequence similarity to transcriptomes and proteomes of related species (not based on a length cutoff). Note that we are unable to assign putative functional characterization to genes without any additional evidence, since such assignment is done based mostly on sequence similarity. Thus, genes that were filtered out are unlikely to affect downstream analysis in significant ways, yet we agree that they may be a useful resource for other subsequent studies.<br><br>Please note the added information within the text extending from line 252 to line 278, which includes the extra references (below for details).<br><br>Korf I. Gene finding in novel genomes. BMC bioinformatics. 2004 Dec;5(1):59.<br><br>Lomsadze A, Ter-Hovhannisyan V, Chernoff YO, Borodovsky M. Gene identification in novel eukaryotic genomes by self-training algorithm, Nucleic Acids Res. , 2005, vol. 33 (pg. 6494-6496)<br><br>Minor comments:<br><br>Lines 261-262: "Drosophila melanogaster" -> use italic type<br>Done.<br><br>Line 265: "A. dux" -> use italic type<br>Done.<br><br>Line 266: "A. dux" -> use italic type<br>Done.
**Additional Information:**	
**Question**	**Response**

Powered by Editorial Manager® and ProduXion Manager® from Aries Systems Corporation

Are you submitting this manuscript to a special series or article collection?	No
**Experimental design and statistics**<br><br>Full details of the experimental design and statistical methods used should be given in the Methods section, as detailed in our Minimum Standards Reporting Checklist. Information essential to interpreting the data presented should be made available in the figure legends.<br><br>Have you included all the information requested in your manuscript?	Yes
**Resources**<br><br>A description of all resources used, including antibodies, cell lines, animals and software tools, with enough information to allow them to be uniquely identified, should be included in the Methods section. Authors are strongly encouraged to cite Research Resource Identifiers (RRIDs) for antibodies, model organisms and tools, where possible.<br><br>Have you included the information requested as detailed in our Minimum Standards Reporting Checklist?	Yes
**Availability of data and materials**<br><br>All datasets and code on which the conclusions of the paper rely must be either included in your submission or deposited in publicly available repositories (where available and ethically appropriate), referencing such data using a unique identifier in the references and in the "Availability of Data and Materials" section of your manuscript.<br><br>Have you have met the above requirement as detailed in our Minimum	Yes

Standards Reporting Checklist?	

Powered by Editorial Manager® and ProduXion Manager® from Aries Systems Corporation

Manuscript

Click here to access/download;Manuscript;RFonseca_main_inclRRID_DOI.do

Click here to view linked References

A draft genome sequence of the elusive giant squid, *Architeuthis dux*

Rute R. da Fonseca*[1,2], Alvarina Couto[3], Andre M. Machado[4], Brona Brejova[5], Carolin B. Albertin[6], Filipe Silva[4,36], Paul Gardner[7], Tobias Baril[8], Alex Hayward[8], Alexandre Campos[4], Ângela M. Ribeiro[4], Inigo Barrio-Hernandez[9], Henk-Jan Hoving[10], Ricardo Tafur-Jimenez[11], Chong Chu[12], Barbara Frazão[4,13], Bent Petersen[14,15], Fernando Peñaloza[16], Francesco Musacchia[17], Graham C. Alexander Jr. [18], Hugo Osório[19,20,21], Inger Winkelmann[22], Oleg Simakov[23], Simon Rasmussen[24], M. Ziaur Rahman[25], Davide Pisani[26], Jakob Vinther[26], Erich Jarvis[27], Guojie Zhang[30,31,32,33], Jan M. Strugnell[34,35], L. Filipe C. Castro[4,36], Olivier Fedrigo[28], Mateus Patricio[29], Qiye Li[37], Sara Rocha[3,16], Agostinho Antunes[4,36], Yufeng Wu[38], Bin Ma[39], Remo Sanges[40,41], Tomas Vinar[5], Blagoy Blagoev[9], Thomas Sicheritz-Ponten[14,15], Rasmus Nielsen[22,42], M. Thomas P. Gilbert[22,43]

[1]Center for Macroecology, Evolution and Climate, Natural History Museum of Denmark, University of Copenhagen, Copenhagen, Denmark.

[2]The Bioinformatics Centre, Department of Biology, University of Copenhagen, Copenhagen, Denmark.

[3]Department of Biochemistry, Genetics and Immunology, University of Vigo, Vigo, Spain.

[4]CIIMAR, Interdisciplinary Centre of Marine and Environmental Research, University of Porto, Porto, Portugal.

[5]Faculty of Mathematics, Physics and Informatics, Comenius University in Bratislava, Bratislava, Slovak Republic.

[6]Department of Organismal Biology and Anatomy, University of Chicago, Chicago, USA.

[7]Department of Biochemistry, University of Otago, New Zealand.

[8]Centre for Ecology and Conservation, University of Exeter, Penryn Campus, Cornwall, UK.

[9]Department of Biochemistry and Molecular Biology, University of Southern Denmark, Odense, Denmark.

[10]GEOMAR Helmholtz Centre for Ocean Research Kiel, Kiel, Germany.

[11]Instituto del Mar del Perú.

[12]Department of Biomedical Informatics, Harvard Medical School, Boston, USA.

[13]IPMA, Fitoplâncton Lab, Lisboa, Portugal.

[14]Centre of Excellence for Omics-Driven Computational Biodiscovery (COMBio), Faculty of Applied Sciences, AIMST University, Kedah, Malaysia.

[15]Evolutionary Genomics Section, Globe Institute, University of Copenhagen, Copenhagen, Denmark

[16]Biomedical Research Center (CINBIO), University of Vigo, Vigo, Spain

[17]Genomic Medicine, Telethon Institute of Genetics and Medicine, Pozzuoli, Naples, Italy

[18]GCB Sequencing and Genomic Technologies Shared Resource, Duke University, Durham, NC, USA.

[19]i3S-Instituto de Investigação e Inovação em Saúde, Universidade do Porto, Porto, Portugal.

[20]IPATIMUP -Institute of Molecular Pathology and Immunology, University of Porto, Porto, Portugal.

[21]Faculty of Medicine of the University of Porto, Porto, Portugal.

[22]Centre for GeoGenetics, Natural History Museum of Denmark, University of Copenhagen, Copenhagen, Denmark.

[23]Department of Molecular Evolution and Development, University of Vienna, Vienna, Austria.

[24]Novo Nordisk Foundation Center for Protein Research, Faculty of Health and Medical Sciences, University of Copenhagen, Copenhagen, Denmark

[25]Bioinformatics Solutions Inc, Waterloo, Ontario, Canada.

[26]Departments of Biological sciences and Earth Sciences, University of Bristol, Bristol, UK.

School of Biological Sciences and School of Earth Sciences, University of Bristol, Bristol, UK.

[27]The Rockefeller University, New York, USA and Howard Hughes Medical Institute, Maryland, USA.

[28]The Rockefeller University, New York, USA

[29]European Molecular Biology Laboratory, European Bioinformatics Institute (EMBL-EBI), Wellcome Genome Campus, Hinxton, UK.

[30]Section for Ecology and Evolution, Department of Biology, University of Copenhagen, Copenhagen, Denmark.

[31]China National Genebank, BGI-Shenzhen, Shenzhen, China.

[32]State Key Laboratory of Genetic Resources and Evolution, Kunming Institute of Zoology, Chinese Academy of Sciences, Kunming, China.

[33]CAS Center for Excellence in Animal Evolution and Genetics, Chinese Academy of Sciences, Kunming, China.

[34]Centre for Sustainable Tropical Fisheries & Aquaculture, James Cook University, Townsville, Queensland, Australia

[35]Department of Ecology, Environment and Evolution, School of Life Sciences, La Trobe University, Melbourne, Victoria, Australia

[36]Department of Biology, Faculty of Sciences, University of Porto, Portugal.

[37]BGI-Shenzhen, Shenzhen, China

[38]Department of Computer Science and Engineering, University of Connecticut, Storrs, USA.

[39]School of Computer Science, University of Waterloo, Canada.

[40]Area of Neuroscience, Scuola Internazionale Superiore di Studi Avanzati (SISSA), Trieste, Italy.

[41]Biology and Evolution of Marine Organisms, Stazione Zoologica Anton Dohrn, Napoli, Italy.

[42]Departments of Integrative Biology and Statistics, University of California, Berkeley, U.S.A.

[43]Norwegian University of Science and Technology, University Museum, Trondheim, Norway

Email addresses:

Rute R da Fonseca: rfonseca@bio.ku.dk (corresponding author)
Alvarina Couto: alvarinacouto@gmail.com
Andre M. Machado: andre.machado@ciimar.up.pt
Brona Brejova: brejova@fmph.uniba.sk
Caroline B.Albertin: calbertin@mbl.edu
Filipe Silva: filipecgsilva@gmail.com
Paul Gardner: paul.gardner@otago.ac.nz
Tobias Baril: tb529@exeter.ac.uk
Alex Hayward Hayward: Alex.Hayward@exeter.ac.uk
Alexandre Campos: acampos@ciimar.up.pt
Ângela M. Ribeiro: ribeiro.angela@gmail.com
Inigo Barrio-Hernandez: ibarrioh@ebi.ac.uk
Henk-Jan Hoving: hhoving@geomar.de
Ricardo Tafur-Jiménez: rtafur@imarpe.gob.pe
Chong Chu: Chong_Chu@hms.harvard.edu
Barbara Frazão: bmfrazao@gmail.com
Bent Petersen: bent.petersen@bio.ku.dk
Fernando Peñaloza: fpenaloz@lcg.unam.mx
Francesco Musacchia: f.musacchia@tigem.it
Graham C. Alexander Jr.:gca2@duke.edu
Hugo Osório: hosorio@ipatimup.pt
Inger E. Winkelmann: inger.winkelmann@gmail.com
Oleg Simakov: oleg.simakov@univie.ac.at
Simon Rasmussen: simon.rasmussen@cpr.ku.dk
M. Ziaur Rahman: zrahman@bioinfor.com
Davide Pisani: Davide.Pisani@bristol.ac.uk
Erich D. Jarvis: ejarvis@rockefeller.edu
Guojie Zhang: zhanggjconi@gmail.com
Jakob Vinther: jakob.vinther@bristol.ac.uk
Jan M. Strugnell: jan.strugnell@jcu.edu.au
L. Filipe C. Castro: filipe.castro@ciimar.up.pt
Olivier Fedrigo: ofedrigo@rockefeller.edu
Mateus Patricio: mateus@ebi.ac.uk
Qiye Li: liqiye@genomics.cn
Sara Rocha: sprocha@gmail.com
Agostinho Antunes: aantunes@ciimar.up.pt
Yufeng Wu: ywu@engr.uconn.edu
Bin Ma: binma@uwaterloo.ca
Remo Sanges: remo.sanges@gmail.com
Tomas Vinar: tomas.vinar@fmph.uniba.sk
Blagoy Blagoev: bab@bmb.sdu.dk
Thomas Sicheritz-Ponten: thomassp@bio.ku.dk

Rasmus Nielsen: rasmus_nielsen@berkeley.edu
M. Thomas P. Gilbert: tgilbert@snm.ku.dk

Abstract

Background

The giant squid (*Architeuthis dux*; Steenstrup, 1857) is an enigmatic giant mollusc with a circumglobal distribution in the deep ocean, except in the high Arctic and Antarctic waters. The elusiveness of the species makes it difficult to study. Thus, having a genome assembled for this deep-sea dwelling species will allow unlocking several pending evolutionary questions.

Findings

We present a draft genome assembly that includes 200 Gb of Illumina reads, 4 Gb of Moleculo synthetic long-reads and 108 Gb of Chicago libraries, with a final size matching the estimated genome size of 2.7 Gb, and a scaffold N50 of 4.8 Mb. We also present an alternative assembly including 27 Gb raw reads generated using the Pacific Biosciences platform. In addition, we sequenced the proteome of the same individual and RNA from three different tissue types from three other species of squid species (*Onychoteuthis banksii*, *Dosidicus gigas*, and *Sthenoteuthis oualaniensis*) to assist genome annotation. We annotated 33,406 protein coding genes supported by evidence and the genome completeness estimated by BUSCO reached 92%. Repetitive regions cover 49.17% of the genome.

Conclusions

This annotated draft genome of *A. dux* provides a critical resource to investigate the unique traits of this species, including its gigantism and key adaptations to deep-sea environments.

Keywords

Cephalopod, invertebrate, genome assembly.

Data description

Context

Cephalopods are the most behaviourally complex of the invertebrate protostomes [1]. Their large, highly differentiated brains are comparable in relative size and complexity to those of vertebrates [2], as are their cognitive capabilities [1]. Cephalopods are distributed worldwide from tropical to polar marine habitats, from benthic to pelagic zones and from intertidal areas down to the abyssal parts of the deep sea, with the only exception being the Black Sea. Cephalopod populations are thought to be currently increasing in some regions for a variety of reasons [3], including potential predator release as a consequence of the depletion of fish stocks [4]. The class Cephalopoda contains approximately 800 species, with the vast majority belonging to the soft-bodied subclass Coleoidea (cuttlefishes, octopuses and squids), and a small handful belonging to the Nautiloidea (nautiluses) [5]. Cephalopods are ecologically important as a primary food source for marine mammals, birds and for many fish species. They are also increasingly important as a high-protein food source for humans and are a growing target for commercial fisheries and farming [6].

Cephalopods show a wide variety of morphologies, lifestyles and behaviours [7], but with the exception of the nautiluses they are characterized by having rapid growth and short lifespans, despite a considerable investment in costly sensory adaptations [2]. They range in size from the tiny pygmy squids (~2 cm) to animals that are nearly three orders of magnitude larger, such as the giant squid, *A. du*x (average length 10–12 m, and reported up to 20 m total length) [6,8,9], to the colossal squid, *Mesonychoteuthis hamiltoni* (maximum length remains unclear, but a recorded weight of 500 kg makes it the largest known invertebrate [10]). Cephalopods can rapidly alter the texture, pattern, colour and brightness of their skin, and this both enables a complex communication system, as wells as provides exceptional camouflage and mimicry [11]. Together these allow cephalopods to both avoid predators, and hunt prey highly efficiently, making them some of the top predators in the ocean. The remarkable adaptations of cephalopods also

extend to their genome, with recent work demonstrating increased levels of RNA editing to diversify proteins involved in neural functions [12].

Over recent years, oceanic warming and acidification, pollution, expanding hypoxia and fishing [13–15] have been shown to affect cephalopod populations. Mercury has been found in high concentrations in the tissue of giant squid specimens [16], and accumulation of flame retardant chemicals has also been detected in the tissue of deep-sea cephalopods [17]. Consequently, there is an urgent need for greater biological understanding of these important, but rarely encountered animals, in order to aid conservation efforts and ensure their continued existence. A genome is an important resource for future population genomics studies aiming at characterizing the diversity of the legendary giant squid, the species which has inspired generations to tell tales of the fabled Kraken.

Methods

DNA extraction, library building, and de novo genome assembly

High-molecular-weight genomic DNA was extracted from a single *A. du*x individual (NCBI taxon id: 256136) using a cetyl trimethylammonium bromide (CTAB) based buffer followed by organic solvent purification, following Winkelmann et al [18] (details in the Supplementary Information). We generated 116 Gb of raw reads from Illumina short-insert libraries, 76 Gb of paired-end reads from libraries ranging from 500 bp to 800 bp in insert size, and 5.4 Gb of mate-pair with a 5 kb insert (Table S1). Furthermore, we generated 3.7 Gb of paired-end reads using Moleculo libraries (3 High-Throughput libraries and 4 High-Fidelity libraries). The kmer distribution of the reads under a diploid model in kmergenie [19] predicted the genome size to be 2.7 Gb.

An initial assembly generated with Meraculous (Meraculous, RRID:SCR_010700) [20] using Illumina and Moleculo data (N50 of 32 Kb, assembly statistics in Table S2) was used as input for Dovetail Genomic's HiRise scaffolding software together with the Hi-C data generated from two Chicago libraries

corresponding to a physical coverage of the genome of 52.1X. This "Meraculous + Dovetail" assembly (statistics in Table 1) was the one used for the genome annotation (non-coding RNAs, protein-coding genes and repeats) and comparative genomics analyses presented in this paper. Further scaffolding was done using 23.38 Gb of PacBio reads (19 SMRT cells, average read length is 14.79 kb) using the default parameters in PBJelly (PBJelly, RRID:SCR_012091) [21] (see assembly statistics in Table S2). The genome gene content completeness was evaluated through the Benchmarking Universal Single-Copy Orthologs (BUSCO, RRID:SCR_015008) v.3.0.2, datasets: Eukaryota, Metazoan) [22].

Transcriptome sequencing and de novo assembly

Given the extreme rarity of live giant squid sightings, we were unable to collect fresh organ samples (following the recommendations in [23]) containing intact RNA from the species to assist with the genome annotation. As an alternative, we extracted total RNA from gonad, liver and brain tissue from live caught specimens of three other oegopsid squid species (*Onychoteuthis banksii*, *Dosidicus gigas*, and *Sthenoteuthis oualaniensis*; NCBI taxon ids 392296, 346249 and 34553, respectively; Supplementary Figure S1), using the Qiagen RNeasy extraction kit (Qiagen,CA, USA). The RNA integrity and quantity was measured on a Qubit fluorometer (Invitrogen, OR, USA) and on the Agilent Bioanalyzer 2100 (Agilent, CA, USA). The Illumina TruSeq Kit v.2.0 was used to isolate the mRNA and prepare cDNA libraries for sequencing, following the recommended protocol. Compatible index sequences were assigned to individual libraries to allow for multiplexing on four lanes of 100bp paired-end technology on an Illumina HiSeq 2000 flow cell. Sequencing of the cDNA libraries was done at the National High-Throughput Sequencing Center at the University of Copenhagen in Denmark. We assessed the quality of the raw reads using FastQC (FastQC, RRID:SCR_014583) v0.10.0 [24]. After removing indexes and adaptors with CutAdapt [25], we trimmed the reads with the FASTX-toolkit (http://hannonlab.cshl.edu/fastx_toolkit) removing bases with a Phred-scale quality score lower than 25. Reference transcriptomes for each species were built after pooling the reads from all tissues and using these as input in Trinity (Trinity,

RRID:SCR_013048) [26]. This software was used with the default settings including a fixed kmer size of 25 as suggested by the authors. Annotation of coding regions was done with the EvidentialGene pipeline [27].

Protein extraction, separation by 1D SDS–PAGE, MALDI-TOF/TOF and Protein Identification

Given the practical impossibility of obtaining RNA from a giant squid specimen, we produced a library of giant squid peptide sequences to guide the gene annotation process.

Proteins were solubilised from a giant squid mantle tissue sample according to the procedure described by Kleffmann et al. [28] and employing the following buffers: (1) 40 mM Tris–HCl, 5 mM MgCl2 and 1 mM DTT, pH 8.5; (2) 8 M urea, 20 mM Tris, 5 mM $MgCl_2$ and 20 mM DTT; (3) 7 M urea, 2 M thiourea, 20 mM Tris, 40 mM DTT, 2% CHAPS (w ⁄ v) and 1% Triton X-100 (v ⁄ v) and (4) 40 mM Tris, 4% SDS (w ⁄ v) and 40 mm DTT. All buffers were augmented with protease inhibitors (Halt™ Protease Inhibitor Cocktail, EDTA-Free, Thermo Scientific). Tissue samples were ground in liquid nitrogen before homogenization, or homogenized directly with ultrasound (probe sonication at 60 Hz, for 3 min) in buffer 1. Solubilised proteins were collected by ultracentrifugation at 100,000xg and 4 ºC. Each extraction was performed in duplicate for each specific buffer and extracts were pooled. Protein extracts were subsequently stored at -20 ºC. Total protein content was estimated according to the Bradford (1976) method [29].

Protein separation by 1D SDS-PAGE electrophoresis was carried out as described in Santos et al. [30]. 53 µL of sample (39 µg protein) was diluted in 72 µL of Loading Buffer (0.01% bromophenol blue, 2% SDS (Sodium-DodecylSulfate), 20% glycerol, 5% β-mercaptoethanol (w/v/v) in 62.5 mM Tris-HCl, pH 6.8). The resulting solution was heated for 3 min at 99°C. Proteins were separated by SDS–PAGE with 12% (w/v) polyacrylamide gels. Electrophoresis was carried out using the mini Protean Cell (BioRad) at a constant voltage of 150 V. The separated proteins were visualized by staining with Colloidal Coomassie Brilliant Blue (CCB) [31], and lanes were cut into 15 gel sections for subsequent LC-MS/MS analysis.

LC-MS/MS analyses

All samples were analysed with the Easy-nLC system (Thermo Fisher Scientific), connected online to a Q Exactive mass spectrometer (Thermo Fisher Scientific) equipped with a nanoelectrospray ion source (Thermo Fisher Scientific). Tryptic peptides were loaded in a fused silica column (75 µm inner diameter) packed with C18 resin (3-µm beads, Reprosil, Dr. Maisch), with solvent A (0.5% acetic acid). They were then eluted with a 120 minute gradient of solvent B (80% ACN, 0.5% acetic acid) with a constant flow of 250 nL/min. The Q exactive was operated in positive mode with a capillary temperature of 250 °C, using the data dependent acquisition method, which switches from full MS scans to MS/MS scans for the 12 most intense ions. Fragmentation was achieved by higher-energy collisional dissociation (HCD) with a normalized collisional energy (NCE) of 25. Full MS ranged from 300 to 1750 m/z at a resolution of 70,000, an Automatic Gain Control (AGC) of 1e6 and a maximum injection time of 120 ms, whereas MS/MS events were scanned at a resolution of 35,000, an AGC of 1e5, maximum injection time of 124 ms, isolation windows of 2 m/z and an exclusion window of 45 seconds.

de novo peptide prediction

Raw LC-MS/MS data were read using Thermo Fisher MSRawFileReader 2.2 library and imported into PEAKS Studio 7.0 and subsequently pre-processed for precursor mass and charge correction, MS/MS de-isotoping, and deconvolution. PEAKS de novo sequencing [31] was performed on each refined MS/MS spectrum with a precursor and fragment ion error tolerance of 7 ppm and 0.02 da respectively. Carbamidomethylation (Cys) was set as a fixed modification and oxidation (Met) and N-terminal Acetylation as variable modifications. At most, five variable modifications per peptide were allowed. For each tandem spectrum, five *de novo* candidates were reported along with their Local Confidence Scores (the likelihood of each amino acid assignment in a de novo candidate peptide). This score was used to determine the accuracy of the de novo peptide sequences. The top de novo peptide for each spectrum was determined by the highest Average Local Confidence score (ALC) among the candidates for that spectrum.

Genome annotation

Protein-coding genes were predicted by ExonHunter [32] , which combines probabilistic models of sequence features with external evidence from alignments. As external evidence, we have used the transcriptomes of oegopsid squid species obtained as a part of this project (*O. banksii*, *D. gigas*, and *S. oualaniensis*); these transcripts were translated into proteins in order to facilitate cross-species comparison. In addition, known proteins from *Octopus bimaculatus*, *Crassostrea gigas* (Pacific oyster) and *Lottia gigantea* (Giant owl limpet) were used to inform the gene prediction process. The proteins were aligned to the genome by BLASTX. De-novo identified MS/MS-based peptides were initially also considered as external evidence but were later omitted due to low coverage. Evidence from predicted repeat locations was used to discourage the model to predict genes overlapping repeats. Since no sufficiently close annotated genome was available for training gene finding parameters, ExonHunter was first run using *Drosophila melanogaster* parameters on a randomly chosen subset of 118 scaffolds longer than 200kb (total length 199 Mb). Out of 12,912 exons predicted in this run, 5,716 were supported by protein alignment data and selected to train the parameters of the gene finding model for *A. dux*, using the methods described in [32]. Such iterative training has been previously shown to yield similar gene prediction results as training on curated gene sets [32–34] [32,xx,yy]. Rerunning ExonHunter with the resulting *A. dux* model parameters on the entire genome yielded 51,225 candidate gene predictions genes. Gene prediction in *A. dux* is challenging due to the fragmentary nature of the genome assembly (60% of predictions span a sequencing gap). This results in a significant number of artifacts, for example short genes with long introns spanning gaps in the assembly. 18,054 predictions yield protein product shorter than 100 amino acids, yet the median span of these predictions is more than 4kb and only 32% of them are supported by transcript or protein alignments. In contrast, 83% of genes with product longer than 100aa are supported. Another factor contributing negatively to gene prediction quality is the lack of RNA-seq data from *A. dux* due to unavailability of fresh organ samples. In most of the analyses below, we consider only 33,406 genes that were found to have transcript evidence (blastp

match to a sequence from a cephalopod transcriptome, with at least 50% of the giant squid coding region covered) and/or matches in Swissprot or UniRef90 databases (Table 1). This supported set contains much fewer extremely short genes (Figure S4).

The function of the protein-coding genes was inferred with Annocript 0.2 [35], which is based on the results from blastp [36] runs against the SwissProt (SP) and UniRef90 (Uf). In addition, we performed a rpsblast search using matrices from the conserved domain database (CDD) to annotate specific domains present on the protein queries.

Non-coding RNAs were annotated using the cmsearch program from INFERNAL 1.1 (INFERNAL, RRID:SCR_011809) and the covariance models (CMs) from the Rfam database v12.0 [37,38]. All matches above the curated GA threshold were included. INFERNAL was selected because it implements the CMs that provide the most accurate bioinformatic annotation tool for ncRNAs available [39]. tRNA-scan v.1.3.1 was subsequently used to refine the annotation of tRNA genes (Table S3). The method uses a number of heuristics to increase the search-speed, annotates the Isoacceptor Type of each prediction, infers if predictions are likely to be functional or tRNA-derived pseudogenes [40,41]. This method uses CMs to identify tRNAs. Rfam matches and the tRNA-scan results for families belonging to the same clan were then "competed", so that only the best match was retained for any genomic region [38].

Transposable element annotation

Repetitive elements were first identified using RepeatMasker (RepeatMasker, RRID:SCR_012954) v.4.0.8 [42] with the eukaryota RepBase [43] repeat library. Low-complexity repeats were ignored (-nolow) and a sensitive (-s) search was performed. Following this, a de novo repeat library was constructed using RepeatModeler (RepeatModeler, RRID:SCR_015027) v.1.0.11 [44] , including RECON v.1.08 [45] and RepeatScout (RepeatScout, RRID:SCR_014653) v.1.0.5 [46]. Novel repeats identified by RepeatModeler were analyzed with a 'BLAST, Extract, Extend' process to characterise elements along their entire length

[47]. Consensus sequences and classification information for each repeat family were generated. The resulting de novo repeat library was utilized to identify repetitive elements using RepeatMasker.

Data analyses

We present a main draft genome assembly produced using 200 Gb of Illumina reads, 4 Gb of Moleculo synthetic long-reads and 108 Gb of Chicago libraries, with a final size matching the estimated genome size of 2.7 Gb, and a scaffold N50 of 4.8 Mb (assembly and annotation statistics in Table 1). Genome completeness estimated by BUSCO reached 90.4% (Eukaryota) and 92.1% (Metazoa), and the completeness for the 33,406 protein-coding genes was 91.2% (Eukaryota) and 84.0 (Metazoa).

We also produced an alternative assembly including 27 Gb raw reads generated using the Pacific Biosciences platform, but this showed minimal improvement in assembly statistics, genome size larger than the predicted and lower BUSCO completeness (Table S2).

Comparative analyses of transposable elements

We estimated the total repeat content of the giant squid genome to be approximately half its total size (~49.1%) (Figure 1, Supplementary Table S4). Out of all the repeats present in the giant squid genome, only a few were predicted to be small RNAs, satellites, simple or low complexity repeats (~0.89% of the total genome), with the vast majority (~48.21%) instead consisting of transposable elements (TEs; i.e. SINEs, LINEs, LTR retrotransposons, and DNA transposons; Figure 1, Supplementary Table S4). Of the TE portion of the giant squid genome, the main contribution from annotated TEs is from DNA elements (11.06%) and LINEs (6.96%), with only a small contribution from SINEs (1.99%) and LTR elements (0.72%). TEs are a nearly universal feature of eukaryotic genomes, often comprising a large proportion of the total genomic DNA (e.g. the maize genome is ~85% TEs [48], stick insect genome is ~52% TEs [49], and the human genome is >45% TEs [50]), consequently these account for the majority of observed genome size variation among animals.

In Figure 1, we summarise the recently reported TE analyses performed on assembled cephalopod

genomes, as follows: California two-spot octopus (*Octopus bimaculatus*) [11] and long-arm octopus (*O. minor*) [51], Hawaiian bobtail squid (*Euprymna scolopes*) [52], and giant squid (*A. dux*). The varying sequencing strategies employed to generate currently available cephalopod genomes (and accompanying variation in assembly quality) complicates the comparative analysis of TE content for this group. However, notwithstanding this caveat, it does seem clear that TEs make up a large fraction of the total genomic content across all cephalopod genomes published to date (Figure 1). DNA transposons and LINEs dominate in available cephalopod genomes, while LTR elements and SINEs generally represent a minor portion of cephalopod TEs (Figure 1). Within decapod cephalopods (i.e. squid and cuttlefish), patterns in TE content are generally similar, however, the giant squid has a notably larger proportion of DNA transposons (1,626,482 elements, 11.06% of the total genome) than the Hawaiian bobtail squid (855,308 elements, 4.05% of the total genome), with the bobtail squid in turn having a similar proportion of LINEs (752,629 elements, 6.83% of the total genome) than the giant squid (766,382 elements, 6.96% of the total genome; Figure 1).

The defining ability of TEs to mobilise, in other words, to transfer copies of themselves into other parts of the genome, can result in harmful mutations. However, TEs can also facilitate the generation of genomic novelty, and there is increasing evidence of their importance for the evolution of host-adaptive processes [53]. In the giant squid genome, all classes of TEs were more frequent (~38.23) in intergenic regions (here defined as regions >2kb upstream or downstream of an annotated gene), than in genic regions versus % of the genome in intergenic regions (~16.6%; Figure 2A). These findings are broadly similar to those reported for other cephalopods, although a larger proportion of the giant squid genome is composed of repeats located within genic regions (percentage of the genome represented by TEs for *O. bimaculoides*: ~6% genic versus ~30% intergenic, and for *O. minor* ~6% genic versus ~40% intergenic [51]).

A Kimura distance-based copy divergence analysis revealed that the most frequent TE sequence divergence relative to the TE consensus sequence in the giant squid genome was ~5–8% across all repeat classes, suggesting a relatively recent transposition burst across all major TE types (Figure 2B). Divergence peaks were most pronounced in LINE RTE elements, Tc/Mar and hAT DNA transposons, and unclassified TEs, with smaller divergence peaks in SINE tRNA elements and Penelope LINE elements (Figure 2B). Divergence peaks were most pronounced in LINE RTE elements, Tc/Mar and hAT DNA transposons, and unclassified TEs, with smaller divergence peaks in SINE tRNA elements and Penelope LINE elements (Figure 2B). In comparison to observations from other cephalopods, these results suggest a shorter and more intense burst of recent TE activity in the giant squid genome. Overall, further genomic sampling within each of the cephalopod clades will be needed to understand TE evolution, as closely related species can show significant differences (*e.g.*, *O. bimaculoides* to *O. vulgaris*) [54].

Non-coding RNAs

We identified 50,598 ncRNA associated loci in the squid sequencing data, using curated homology-based probabilistic models from the Rfam database [55] and the specialized tRNAscan-SE (tRNAscan-SE, RRID:SCR_010835) transfer RNA annotation tool [40]. The essential and well conserved Metazoan ncRNAs: tRNAs, rRNAs (5S, 5.8S, SSU and LSU), RNase P, RNase MRP, SRP and the major spliceosomal snRNAs (U1, U2, U4, U5, U6), as well as the minor spliceosomal snRNAs (U11, U12, U4atac & U6atac), are all found in the *A. dux* genome. Some of the copy numbers associated with the core ncRNAs are extreme. For example, we identified: i) approximately 24,000 loci that appear to derive from 5S rRNA; ii) approximately 17,000 loci that are predicted to be tRNA derived; iii) approximately 3,200 Valine tRNAs isotypes and approximately 1,300 U2 spliceosomal RNAs. The microRNA mir-598 also exhibits high copy-numbers at 172. Many of these are likely to be SINEs derived by transposition. All 20 tRNA isotypes were identified in *A. dux* genome. Again, many of these had relatively large copy numbers (summarised in Table 1). These ranged from 46 (Cys) up to 2,541 (Val). We identified 174 loci that share homology with

34 known snoRNA families, these included 15 scaRNA, 41 H/ACA box and 118 C/D box snoRNA associated loci [10]. The snoRNAs are predominantly involved in rRNA maturation. We identified 7,049 loci that share homology with 283 families of microRNA. Some of these may be of limited reliability, as CMs for simple hairpin structures can also match other, non-homologous, hairpin-like structures in the genome e.g. inverted repeats. A number of cis-regulatory elements were also identified. These included 235 hammerhead 1 ribozymes, 133 Histone 30 UTR stem-loops, and 14 Potassium channel RNA editing signal sequences. There are very few matches to obvious non-metazoan RNA families in the current assemblies. The only notable exceptions are bablM, IMES-2, PhotoRC-II and rspL. Each of these families are also found in marine metagenomic datasets, possibly explaining their presence as “contamination” from the environment.

Analyses of specific gene families

Several gene families involved in development, such as transcription factors or signaling ligands, are highly conserved across metazoans and may therefore reveal signatures of genomic events, such as a whole genome duplication.

WNT is a family of secreted lipid-modified signaling glycoproteins with a key role during development [56]. Comparative analysis of molluscan genomes indicates that the ancestral state was 12 *WNT* genes, as *Wnt3* is absent in all protostomes examined thus far [57]. The giant squid has the typical 12 lophotrochozoan WNTs (1, 2, 4, A, 5, 6, 7, 8, 9, 10, 11 and 16; Supplementary Figure S2), and therefore has retained the ancestral molluscan complement, including *Wnt8*, which is absent, for instance, in the genome of the slipper snail *Lottia gigantea* [58].

Protocadherins are a family of cell adhesion molecules that appear to play an important role in vertebrate brain development [59]. It is thought that they act as multimers at the cell surface in a manner akin to DSCAM in flies, which lack protocadherins [60]. Cephalopods have massively expanded

this family, with 168 identified in the *O. bimaculoides* genome, whereas only 17-25 protocadherins have been identified in the genomes of annelids and non-cephalopod molluscs [11]. We identified approximately 135 protocadherin genes in *A. dux*, many of which are located in clusters in the genome. The possibility that this gene family plays a developmental role parallel to that of protocadherins in vertebrate neurodevelopment thus remains a compelling hypothesis.

Development organisation of the highly diverse body plans found in the Metazoa is controlled by a conserved cluster of homeotic genes, which includes, among others, the Hox genes. These are characterized by a DNA sequence referred to as the homeobox, comprising 180 nucleotides that encode the homeodomain [61]. Hox genes are usually found in tight physical clusters in the genome and are sequentially expressed in the same chronological order as they are physically located in the DNA (temporal and spatial collinearity) [62]. Different combinations of Hox gene expression in the same tissue type can lead to a wide variety of different structures [63]. This makes the Hox genes a key subject for understanding the origins of the multitude of forms found in the cephalopods. In *O. bimaculoides* genome assembly no scaffold contained more than a single Hox gene, meaning that they are fully atomised [11]. However, in *E. scolopes*, the Hox cluster was found spanning two scaffolds [52]. In the giant squid, we recovered a full Hox gene cluster in a single scaffold (Figure 3-B). The Hox gene organization found in the giant squid genome suggests either the presence of a disorganised cluster, so-called type D, or atomised clusters, type A [63], or possibly a combination of the two (the genes are still organized, but physically distant from each other). The existence of a "true" cluster seems unlikely, given the presence of other unrelated genes in between and the relatively large distances (Figure 3-C). The classification as type A (atomised) might seem most obvious, despite the co-presence of the genes in a single scaffold, due to these large distances. However, the definition of type D (disorganised) does allow for the presence of non-Hox genes in between members of the cluster (Figure 3-A). Thus, it is difficult to clearly categorise the recovered "cluster", but it does remain clear that these genes are not as tightly

bundled as they are in other Bilateria lineages. The *A. dux* Hox "cluster" is spread across 11 Mb of a 38 Mb scaffold, and this suggests a far larger size range in the cephalopods than in other described animals, as recently suggested based on the genome of *E. scolopes* [52]. It is possible that this is the reason for the apparent atomisation of Hox genes in the more fragmented *O. bimaculoides* assembly. Hox clusters are usually found in contigs of around 100 kb length in vertebrates [6, 7] and between 500 – 10,000 kb in invertebrates [8] An assembled contig easily containing the complete cluster for these smaller cluster sizes, would manage to cover only one member of the Hox gene cluster in the studied coleoids. As such, our results suggest that the Hox cluster may not be fully atomised in *O. bimaculoides* as previously hypothesised. Further improvements of genome assemblies in cephalopods will be required to address this question. The biological reason for this dramatic increase in the distance between the genes in the Hox cluster presents an intriguing avenue of future research. The homeodomain of all the obtained Hox genes in cephalopods were compared with those of other mollusks. Few differences were found relative to a previous study [64], as no significant modifications were observed in Hox1, Hox4, ANTP, Lox2, Lox5, Post1 and Post2. Hox1 did, however, show reduced conservation in residues 22 to 25 in the *A. dux* sequence. This observation for Hox1 in *A. dux* is visible only in the Pacbio assembly. Additionally, the Hox3 homeodomain analysis supports a basal placement of the nautiloids within cephalopods. The Lox4 gene was the most variable among all groups. As of to date, Hox2 still remains undetected in the coleoid cephalopods [65]. Assembly errors notwithstanding, gain and loss of Hox genes has been attributed to fundamental changes in animal body plans, and the apparent loss of Hox2 may therefore be significant. For example, Hox gene loss has been associated with the reduced body-plan segmentation of spider mites [42]. The circumstance that Hox2 has been readily found in *Nautilus*, but remains undetected in all coleoids sequenced thus far, might signify an important developmental split within the Cephalopoda. Alternatively, and equally intriguing, this Hox gene may have undergone such drastic evolutionary modifications that it is presently undetectable by conventional means.

On a final note, we analyzed genes encoding reflectins, a class of cephalopod-specific proteins first described in *E. scolopes* [66]. Reflectins form flat structures that reflect ambient light (other marine animals use purine-based platelets), thus modulating iridescence for communication or camouflage purposes [67]. The giant squid genome contains seven reflectin genes and three reflectin-like genes (Supplementary Figure S3). All of these genes, with the exception of one reflectin gene, appear on the same scaffold, which corresponds very well with the distribution pattern of octopus reflectin genes [11]).

Conclusions

Not only because of its astonishing proportions, but also for the lack of knowledge of the key facets of its deep-sea lifestyle, the giant squid has long captured the imagination of scientists and the general public alike. With the release of this annotated giant squid genome, we set the stage for future research into the enigmas that enshroud this truly awe-inspiring creature. Further, given the paucity of available cephalopod genomes, we provide a valuable contribution to the genomic description of cephalopods, and more widely to the growing number of fields that are recognizing the potential, which this group of behaviourally advanced invertebrates holds for improving our understanding of the diversity of life on Earth in general.

Availability of supporting data

The data sets supporting the results of this article are available in the NCBI database via Bioproject PRJNA534469. The three transcriptome data sets (TSA) have ids GHKK01000000, GHKL01000000 and GHKH01000000 and the sequence data used for the genome assemblies has id VCCN01000000.Proteomics data are available via ProteomeXchange with identifier PXD016522. Supporting data is also available via the *Gigascience* repository GigaDB [69].

Additional files

Supplement.txt. Supplementary methods, tables and figures.

Declarations

Abbreviations

Gb: gigabase pairs; Mb: megabase pairs; BUSCO: Benchmarking Universal Single-copy Orthologs; bp: base pair; NCBI: National Center for Biotechnology Information; LC-MS/MS: liquid chromatography (LC) tandem mass spectrometry (MS); CCB: Colloidal Coomassie Brilliant Blue; HCD: higher-energy collisional dissociation; NCE: normalized collisional energy; AGC: Automatic Gain Control; ALC: Average Local Confidence; SP: SwissProt; Uf: UniRef90; CDD: conserved domain database; CM: covariance model; TE: transposable element; LINE: Long interspersed nuclear element; SINE: Short interspersed nuclear element; LRT: long terminal repeat.

Ethics statement

Sampling followed the recommendations from Moltschaniwskyj et al., 2007 [23].

Consent for publication

Not applicable.

Competing interests

The authors declare that they have no competing interests.

Funding

R.R.F. thanks the Villum Fonden for grant VKR023446 (Villum Fonden Young Investigator Grant), the Portuguese Science Foundation (FCT) for grant PTDC/MAR/115347/2009;COMPETE-FCOMP-01-012; FEDER-015453, Marie Curie Actions (FP7-PEOPLE-2010-IEF, Proposal 272927), and the Danish National Research Foundation (DNRF96) for its funding of the Center for Macroecology, Evolution, and Climate.

H.O. thanks the Rede Nacional de Espectrometria de Massa, ROTEIRO/0028/2013, ref. LISBOA-01-0145-FEDER-022125, supported by COMPETE and North Portugal Regional Operational Programme (Norte2020), under the PORTUGAL 2020 Partnership Agreement, through the European Regional Development Fund (ERDF). A.C. thanks FCT for project UID/Multi/04423/2019. M.P. acknowledges the support from the Wellcome Trust (grant number WT108749/Z/15/Z) and the European Molecular Biology Laboratory. M.P.T.G. thanks the Danish National Research Foundation for its funding of the Center for GeoGenetics (grant DNRF94) and Lundbeck Foundation for grant R52-5062 on Pathogen Palaeogenomics. S.R. was supported by the Novo Nordisk Foundation grant NNF14CC0001. A.H. is supported by a Biotechnology and Biological Sciences Research Council David Phillips Fellowship [fellowship reference: BB/N020146/1]. T.B. is supported by the Biotechnology and Biological Sciences Research Council-funded South West Biosciences Doctoral Training Partnership [training grant reference BB/M009122/1]. This work was partially funded by the Lundbeck Foundation (R52-A4895 to BB).

HJTH was supported by the David and Lucile Packard Foundation, the Netherlands Organization for Scientific Research (#825.09.016) and currently by the Deutsche Forschungsgemeinschaft (DFG) under grant HO 5569/2-1 (Emmy Noether Junior Research Group). T.V. and B.Br. were supported by grants from the Slovak grant agency VEGA (1/0684/16, 1/0458/18). F.S. was supported by a PhD grant (SFRH/BD/126560/2016) from FCT. A.A. was partly supported by the FCT project PTDC/CTA-AMB/31774/2017. C.C. and Y.W. are partly supported by grant IIS-1526415 from US National Science Foundation. Computation for the work described in this paper was partially supported by the DeiC National Life Science Supercomputer at DTU.

Authors contributions

R.D.F. and M.T.P.G. designed the study. J.S., H-J.H. AND R.T. carried out the sampling. Alex.C., A.R., B.F., G.C.A.Jr, H.O. and I.W. performed the laboratory work. R.D.F., Alv.C., A.M., C.B.A., F.S., P.G., T.B., A.H., I.B.H., C.C., B.P., F.P., M.P., F.M., O.S., S.R., M.Z.R. and D.P. analyzed the data. E.J., G.Z., J.V., O.F. and Q.L.

contributed with genomic resources. R.D.F., L.F.C.C., A.A., Y.W., B.M., R.S., T.V., B.B., T.S-P., M.T.P.G. contributed with supervision and computational resources. R.R.F., T.S-P., R.N., M.T.P.G paid for sequencing. R.D.F. wrote the manuscript with contributions from all authors. All authors have read and approved the manuscript.

Acknowledgments

We would like to thank Anders Hansen, Tobias Mourier, Kristin Rós Kjartansdóttir and Lars Hestbjerg Hansen for help with generating sequencing data; Shawn Hoon for sharing transcriptome data; Annie Lingren for help with sample shipping; Peter Smith for providing samples and the support of the entire team at Dovetail.

References

1. Zullo L, Hochner B. A new perspective on the organization of an invertebrate brain. Commun. Integr. Biol. [Internet]. Taylor & Francis; 2011 [cited 2019 Feb 19];4:26–9. Available from: http://www.ncbi.nlm.nih.gov/pubmed/21509172

2. Nixon M, Young JZ. The brains and lives of cephalopods. Oxford: Oxford University Press, Oxford; 2003.

3. Doubleday ZA, Prowse TAA, Arkhipkin A, Pierce GJ, Semmens J, Steer M, et al. Global proliferation of cephalopods. Curr. Biol. [Internet]. 2016 [cited 2019 May 2];26:R406–7. Available from: http://www.ncbi.nlm.nih.gov/pubmed/27218844

4. Vecchione M, Allcock L, Piatkowski U, Jorgensen E, Barratt I. Persistent Elevated Abundance of Octopods in an Overfished Antarctic Area. Smithson. Poles Contrib. to Int. Polar Year Sci. [Internet]. Smithsonian Institution Scholarly Press; 2009 [cited 2019 May 2]. p. 197–204. Available from:

https://repository.si.edu/handle/10088/6827

5. Young RE, Vecchione M, Mangold KM. Cephalopoda, Cuvier 1797 [Internet]. Tree Life. 2018. Available from: http://tolweb.org/Cephalopoda/19386

6. Roper CF, Sweeney MJ, Nauen CE. FAO Species Catalogue Vol. 3. Cephalopods of the world. An annotated and illustrated catalogue of species of interest to fisheries. FAO Fish. Synopsis [Internet]. Rome; 1984;125:277. Available from: http://www.fao.org/3/ac479e/ac479e00.htm

7. Jereb P, Roper CFE. Cephalopods of the world. An annotated and illustrated catalogue of cephalopod species known to date. Myopsid and Oegopsid Squids. FAO Species Cat. Fish. Purp. [Internet]. Food and Agriculture Organization of the United Nations; 2010 [cited 2019 Feb 19];2:605. Available from: http://www.fao.org/3/i1920e/i1920e00.htm

8. McClain CR, Balk MA, Benfield MC, Branch TA, Chen C, Cosgrove J, et al. Sizing ocean giants: patterns of intraspecific size variation in marine megafauna. PeerJ [Internet]. PeerJ Inc.; 2015 [cited 2019 May 15];3:e715. Available from: https://peerj.com/articles/715

9. Paxton CGM. Unleashing the Kraken: on the maximum length in giant squid (Architeuthis sp.). J. Zool. [Internet]. 2016 [cited 2019 May 15];300:82–8. Available from: https://zslpublications.onlinelibrary.wiley.com/doi/pdf/10.1111/jzo.12347

10. Rosa R, Seibel BA. Slow pace of life of the Antarctic colossal squid. J. Mar. Biol. Assoc. United Kingdom [Internet]. Cambridge University Press; 2010 [cited 2019 May 2];90:1375–8. Available from: https://www.cambridge.org/core/product/identifier/S0025315409991494/type/journal_article

11. Albertin CB, Simakov O, Mitros T, Wang ZY, Pungor JR, Edsinger-Gonzales E, et al. The octopus genome and the evolution of cephalopod neural and morphological novelties. Nature [Internet]. Nature Publishing Group; 2015 [cited 2018 May 2];524:220–4. Available from:

http://www.nature.com/articles/nature14668

12. Liscovitch-Brauer N, Alon S, Porath HT, Elstein B, Unger R, Ziv T, et al. Trade-off between Transcriptome Plasticity and Genome Evolution in Cephalopods. Cell [Internet]. 2017 [cited 2019 May 2];169:191–202.e11. Available from: http://www.ncbi.nlm.nih.gov/pubmed/28388405

13. Gilly WF, Beman JM, Litvin SY, Robison BH. Oceanographic and Biological Effects of Shoaling of the Oxygen Minimum Zone. Ann. Rev. Mar. Sci. [Internet]. 2013 [cited 2019 Feb 19];5:393–420. Available from: http://www.ncbi.nlm.nih.gov/pubmed/22809177

14. Golikov AV, Sabirov RM, Lubin PA, Jørgensen LL. Changes in distribution and range structure of Arctic cephalopods due to climatic changes of the last decades. Biodiversity [Internet]. Taylor & Francis Group ; 2013 [cited 2019 Feb 19];14:28–35. Available from: http://www.tandfonline.com/doi/abs/10.1080/14888386.2012.702301

15. Balmaseda MA, Trenberth KE, Källén E. Distinctive climate signals in reanalysis of global ocean heat content. Geophys. Res. Lett. [Internet]. John Wiley & Sons, Ltd; 2013 [cited 2019 Feb 19];40:1754–9. Available from: http://doi.wiley.com/10.1002/grl.50382

16. Bustamante P, González AF, Rocha F, Miramand P, Guerra A. Metal and metalloid concentrations in the giant squid Architeuthis dux from Iberian waters. Mar. Environ. Res. [Internet]. 2008 [cited 2019 Feb 19];66:278–87. Available from: http://www.ncbi.nlm.nih.gov/pubmed/18514304

17. Unger MA, Harvey E, Vadas GG, Vecchione M. Persistent pollutants in nine species of deep-sea cephalopods. Mar. Pollut. Bull. [Internet]. 2008 [cited 2019 Feb 19];56:1498–500. Available from: https://linkinghub.elsevier.com/retrieve/pii/S0025326X0800218X

18. Winkelmann I, Campos PF, Strugnell J, Cherel Y, Smith PJ, Kubodera T, et al. Mitochondrial genome diversity and population structure of the giant squid Architeuthis: genetics sheds new light on one of the

most enigmatic marine species. Proceedings. Biol. Sci. [Internet]. 2013 [cited 2019 Apr 4];280:20130273. Available from: http://rspb.royalsocietypublishing.org/cgi/doi/10.1098/rspb.2013.0273

19. Chikhi R, Medvedev P. Informed and automated k-mer size selection for genome assembly. Bioinformatics [Internet]. Oxford University Press; 2014 [cited 2016 Aug 11];30:31–7. Available from: http://www.ncbi.nlm.nih.gov/pubmed/23732276

20. Chapman J a, Ho I, Sunkara S, Luo S, Schroth GP, Rokhsar DS. Meraculous: de novo genome assembly with short paired-end reads. PLoS One [Internet]. 2011 [cited 2013 Feb 28];6:e23501. Available from: http://www.pubmedcentral.nih.gov/articlerender.fcgi?artid=3158087&tool=pmcentrez&rendertype=abstract

21. English AC, Richards S, Han Y, Wang M, Vee V, Qu J, et al. Mind the Gap: Upgrading Genomes with Pacific Biosciences RS Long-Read Sequencing Technology. Liu Z, editor. PLoS One [Internet]. 2012 [cited 2019 Apr 4];7:e47768. Available from: http://www.ncbi.nlm.nih.gov/pubmed/23185243

22. Simão FA, Waterhouse RM, Ioannidis P, Kriventseva E V., Zdobnov EM. BUSCO: assessing genome assembly and annotation completeness with single-copy orthologs. Bioinformatics [Internet]. 2015 [cited 2019 Apr 8];31:3210–2. Available from: https://academic.oup.com/bioinformatics/article-lookup/doi/10.1093/bioinformatics/btv351

23. Moltschaniwskyj NA, Hall K, Lipinski MR, Marian JEAR, Nishiguchi M, Sakai M, et al. Ethical and welfare considerations when using cephalopods as experimental animals. Rev. Fish Biol. Fish. [Internet]. Kluwer Academic Publishers; 2007 [cited 2019 Jun 22];17:455–76. Available from: http://link.springer.com/10.1007/s11160-007-9056-8

24. Patel RK, Jain M. NGS QC Toolkit: A Toolkit for Quality Control of Next Generation Sequencing Data. Liu Z, editor. PLoS One [Internet]. Public Library of Science; 2012 [cited 2018 Jul 31];7:e30619. Available

from: http://dx.plos.org/10.1371/journal.pone.0030619

25. Martin M. Cutadapt removes adapter sequences from high-throughput sequencing reads. EMBnet.journal [Internet]. 2011;17:10–2. Available from: http://journal.embnet.org/index.php/embnetjournal/article/view/200

26. Haas BJ, Papanicolaou A, Yassour M, Grabherr M, Blood PD, Bowden J, et al. De novo transcript sequence reconstruction from RNA-seq using the Trinity platform for reference generation and analysis. Nat. Protoc. [Internet]. NIH Public Access; 2013 [cited 2018 Jun 24];8:1494–512. Available from: http://www.ncbi.nlm.nih.gov/pubmed/23845962

27. Gilbert D. Gene-omes built from mRNA seq not genome DNA [Internet]. Notre Dame: 7th annual arthropod genomics symposium; 2013. Available from: http://globalhealth.nd.edu/7th-annual-arthropod-genomics-symposium/

28. Kleffmann T, Russenberger D, von Zychlinski A, Christopher W, Sjölander K, Gruissem W, et al. The Arabidopsis thaliana chloroplast proteome reveals pathway abundance and novel protein functions. Curr. Biol. [Internet]. 2004 [cited 2014 Aug 23];14:354–62. Available from: http://www.ncbi.nlm.nih.gov/pubmed/15028209

29. Bradford MM. A rapid and sensitive method for the quantitation of microgram quantities of protein utilizing the principle of protein-dye binding. Anal. Biochem. [Internet]. 1976 [cited 2019 Mar 28];72:248–54. Available from: http://www.ncbi.nlm.nih.gov/pubmed/942051

30. Santos R, da Costa G, Franco C, Gomes-Alves P, Flammang P, Coelho A V. First Insights into the Biochemistry of Tube Foot Adhesive from the Sea Urchin Paracentrotus lividus (Echinoidea, Echinodermata). Mar. Biotechnol. [Internet]. 2009 [cited 2019 Mar 28];11:686–98. Available from: http://www.ncbi.nlm.nih.gov/pubmed/19221839

31. Neuhoff V, Arold N, Taube D, Ehrhardt W. Improved staining of proteins in polyacrylamide gels including isoelectric focusing gels with clear background at nanogram sensitivity using Coomassie Brilliant Blue G-250 and R-250. Electrophoresis [Internet]. John Wiley & Sons, Ltd; 1988 [cited 2019 Mar 28];9:255–62. Available from: http://doi.wiley.com/10.1002/elps.1150090603

32. Brejová B, Vinar T, Chen Y, Wang S, Zhao G, Brown DG, et al. Finding genes in Schistosoma japonicum: annotating novel genomes with help of extrinsic evidence. Nucleic Acids Res. [Internet]. 2009 [cited 2016 Mar 10];37:e52. Available from: http://www.pubmedcentral.nih.gov/articlerender.fcgi?artid=2673418&tool=pmcentrez&rendertype=abstract

33. Korf I. Gene finding in novel genomes. BMC Bioinformatics [Internet]. BioMed Central; 2004 [cited 2016 Feb 17];5:59. Available from: http://bmcbioinformatics.biomedcentral.com/articles/10.1186/1471-2105-5-59

34. Lomsadze A, Ter-Hovhannisyan V, Chernoff YO, Borodovsky M. Gene identification in novel eukaryotic genomes by self-training algorithm. Nucleic Acids Res. [Internet]. 2005 [cited 2019 Nov 13];33:6494–506. Available from: http://www.ncbi.nlm.nih.gov/pubmed/16314312

35. Musacchia F, Basu S, Petrosino G, Salvemini M, Sanges R. Annocript: a flexible pipeline for the annotation of transcriptomes able to identify putative long noncoding RNAs. Bioinformatics [Internet]. 2015 [cited 2016 Mar 14];31:2199–201. Available from: http://bioinformatics.oxfordjournals.org/content/early/2015/02/19/bioinformatics.btv106

36. Camacho C, Coulouris G, Avagyan V, Ma N, Papadopoulos J, Bealer K, et al. BLAST+: architecture and applications. BMC Bioinformatics [Internet]. 2009 [cited 2014 Jul 9];10:421. Available from: http://www.pubmedcentral.nih.gov/articlerender.fcgi?artid=2803857&tool=pmcentrez&rendertype=abstract

37. Burge SW, Daub J, Eberhardt R, Tate J, Barquist L, Nawrocki EP, et al. Rfam 11.0: 10 years of RNA families. Nucleic Acids Res. [Internet]. Narnia; 2013 [cited 2019 Apr 4];41:D226–32. Available from: http://academic.oup.com/nar/article/41/D1/D226/1050811/Rfam-110-10-years-of-RNA-families

38. Gardner PP, Daub J, Tate J, Moore BL, Osuch IH, Griffiths-Jones S, et al. Rfam: Wikipedia, clans and the "decimal" release. Nucleic Acids Res. [Internet]. Narnia; 2011 [cited 2019 Apr 4];39:D141–5. Available from: https://academic.oup.com/nar/article-lookup/doi/10.1093/nar/gkq1129

39. Freyhult EK, Bollback JP, Gardner PP. Exploring genomic dark matter: a critical assessment of the performance of homology search methods on noncoding RNA. Genome Res. [Internet]. Cold Spring Harbor Laboratory Press; 2007 [cited 2019 Apr 4];17:117–25. Available from: http://www.ncbi.nlm.nih.gov/pubmed/17151342

40. Chan PP, Lowe TM. GtRNAdb: a database of transfer RNA genes detected in genomic sequence. Nucleic Acids Res. [Internet]. Narnia; 2009 [cited 2019 Apr 4];37:D93–7. Available from: https://academic.oup.com/nar/article-lookup/doi/10.1093/nar/gkn787

41. Lowe TM, Eddy SR. tRNAscan-SE: a program for improved detection of transfer RNA genes in genomic sequence. Nucleic Acids Res. [Internet]. 1997 [cited 2019 Apr 4];25:955–64. Available from: http://www.ncbi.nlm.nih.gov/pubmed/9023104

42. Smit AFA, Hubley RR, Green PR. RepeatMasker Open-4.0. 2013.

43. Bao W, Kojima KK, Kohany O. Repbase Update, a database of repetitive elements in eukaryotic genomes. Mob. DNA [Internet]. 2015 [cited 2019 Apr 17];6:11. Available from: http://www.ncbi.nlm.nih.gov/pubmed/26045719

44. Smit A, Hubley R. RepeatModeler Open-1.0. 2015.

45. Bao Z, Eddy SR. Automated de novo identification of repeat sequence families in sequenced

genomes. Genome Res. [Internet]. 2002 [cited 2019 Apr 17];12:1269–76. Available from: http://www.genome.org/cgi/doi/10.1101/gr.88502

46. Price AL, Jones NC, Pevzner PA. De novo identification of repeat families in large genomes. Bioinformatics [Internet]. 2005 [cited 2019 Apr 17];21:i351–8. Available from: http://www.ncbi.nlm.nih.gov/pubmed/15961478

47. Platt RN, Blanco-Berdugo L, Ray DA. Accurate Transposable Element Annotation Is Vital When Analyzing New Genome Assemblies. Genome Biol. Evol. [Internet]. 2016 [cited 2019 Apr 17];8:403–10. Available from: https://academic.oup.com/gbe/article-lookup/doi/10.1093/gbe/evw009

48. Schnable PS, Ware D, Fulton RS, Stein JC, Wei F, Pasternak S, et al. The B73 Maize Genome: Complexity, Diversity, and Dynamics. Science (80-.). [Internet]. 2009 [cited 2019 Apr 17];326:1112–5. Available from: http://www.ncbi.nlm.nih.gov/pubmed/19965430

49. Wu C, Twort VG, Crowhurst RN, Newcomb RD, Buckley TR. Assembling large genomes: analysis of the stick insect (Clitarchus hookeri) genome reveals a high repeat content and sex-biased genes associated with reproduction. BMC Genomics [Internet]. 2017 [cited 2019 Apr 17];18:884. Available from: http://www.ncbi.nlm.nih.gov/pubmed/29145825

50. Initial sequencing and analysis of the human genome. Nature [Internet]. Nature Publishing Group; 2001 [cited 2019 Apr 17];409:860–921. Available from: http://www.nature.com/articles/35057062

51. Kim B-M, Kang S, Ahn D-H, Jung S-H, Rhee H, Yoo JS, et al. The genome of common long-arm octopus Octopus minor. Gigascience [Internet]. 2018 [cited 2019 Apr 17];7. Available from: http://www.ncbi.nlm.nih.gov/pubmed/30256935

52. Belcaid M, Casaburi G, McAnulty SJ, Schmidbaur H, Suria AM, Moriano-Gutierrez S, et al. Symbiotic organs shaped by distinct modes of genome evolution in cephalopods. Proc. Natl. Acad. Sci. U. S. A.

[Internet]. National Academy of Sciences; 2019 [cited 2019 Apr 17];116:3030–5. Available from: http://www.ncbi.nlm.nih.gov/pubmed/30635418

53. Schrader L, Schmitz J. The impact of transposable elements in adaptive evolution. Mol. Ecol. [Internet]. 2018 [cited 2019 Apr 17];28:1537–49. Available from: http://www.ncbi.nlm.nih.gov/pubmed/30003608

54. Zarrella I, Herten K, Maes GE, Tai S, Yang M, Seuntjens E, et al. The survey and reference assisted assembly of the Octopus vulgaris genome. Sci. Data [Internet]. Nature Publishing Group; 2019 [cited 2019 Jun 9];6:13. Available from: http://www.nature.com/articles/s41597-019-0017-6

55. Nawrocki EP, Burge SW, Bateman A, Daub J, Eberhardt RY, Eddy SR, et al. Rfam 12.0: updates to the RNA families database. Nucleic Acids Res. [Internet]. 2015 [cited 2019 Apr 4];43:D130–7. Available from: http://www.ncbi.nlm.nih.gov/pubmed/25392425

56. Cadigan KM, Nusse R. Wnt signaling: a common theme in animal development. Genes Dev. [Internet]. Cold Spring Harbor Laboratory Press; 1997 [cited 2019 May 8];11:3286–305. Available from: http://www.ncbi.nlm.nih.gov/pubmed/9407023

57. Cho S-J, Valles Y, Giani VC, Seaver EC, Weisblat DA. Evolutionary Dynamics of the wnt Gene Family: A Lophotrochozoan Perspective. Mol. Biol. Evol. [Internet]. 2010 [cited 2019 May 16];27:1645–58. Available from: http://www.ncbi.nlm.nih.gov/pubmed/20176615

58. Simakov O, Marletaz F, Cho S-J, Edsinger-Gonzales E, Havlak P, Hellsten U, et al. Insights into bilaterian evolution from three spiralian genomes. Nature [Internet]. 2012 [cited 2019 May 16];493:526–31. Available from: http://www.ncbi.nlm.nih.gov/pubmed/23254933

59. Chen W V, Maniatis T. Clustered protocadherins. Development [Internet]. Company of Biologists; 2013 [cited 2018 Oct 21];140:3297–302. Available from:

http://www.ncbi.nlm.nih.gov/pubmed/23900538

60. Zipursky SL, Sanes JR. Chemoaffinity revisited: dscams, protocadherins, and neural circuit assembly. Cell [Internet]. 2010 [cited 2019 May 16];143:343–53. Available from: https://linkinghub.elsevier.com/retrieve/pii/S0092867410011451

61. Pratihar S, Prasad Nath R, Kumar Kundu J. Hox genes and its role in animal development. Int. J. Sci. Nat. [Internet]. 2010 [cited 2019 Apr 4];1:101–3. Available from: http://www.scienceandnature.org/IJSN_V1(2)_D2010/IJSN_V1(2)_2.pdf

62. Fröbius AC, Matus DQ, Seaver EC. Genomic Organization and Expression Demonstrate Spatial and Temporal Hox Gene Colinearity in the Lophotrochozoan Capitella sp. I. Butler G, editor. PLoS One [Internet]. 2008 [cited 2019 Apr 4];3:e4004. Available from: http://www.ncbi.nlm.nih.gov/pubmed/19104667

63. Mallo M, Wellik DM, Deschamps J. Hox genes and regional patterning of the vertebrate body plan. Dev. Biol. [Internet]. 2010 [cited 2019 Apr 4];344:7–15. Available from: http://www.ncbi.nlm.nih.gov/pubmed/20435029

64. Pernice M, Deutsch JS, Andouche A, Boucher-Rodoni R, Bonnaud L. Unexpected variation of Hox genes' homeodomains in cephalopods. Mol. Phylogenet. Evol. [Internet]. Academic Press; 2006 [cited 2019 Apr 4];40:872–9. Available from: https://www.sciencedirect.com/science/article/pii/S1055790306001369?via%3Dihub

65. Barucca M, Canapa A, Biscotti MA, Zappavigna V. An Overview of Hox Genes in Lophotrochozoa: Evolution and Functionality. J. Dev. Biol. [Internet]. Multidisciplinary Digital Publishing Institute (MDPI); 2016 [cited 2019 Apr 4];4:1–15. Available from: http://www.ncbi.nlm.nih.gov/pubmed/29615580

66. Crookes WJ, Ding L-L, Huang QL, Kimbell JR, Horwitz J, McFall-Ngai MJ. Reflectins: The Unusual

Proteins of Squid Reflective Tissues. Science (80-.). [Internet]. 2004 [cited 2019 Feb 19];303:235–8. Available from: http://www.ncbi.nlm.nih.gov/pubmed/14716016

67. Wardill TJ, Gonzalez-Bellido PT, Crook RJ, Hanlon RT. Neural control of tuneable skin iridescence in squid. Proc. R. Soc. B Biol. Sci. [Internet]. The Royal Society; 2012 [cited 2019 May 16];279:4243–52. Available from: http://rspb.royalsocietypublishing.org/cgi/doi/10.1098/rspb.2012.1374

68. Pace RM, Grbić M, Nagy LM. Composition and genomic organization of arthropod Hox clusters. Evodevo [Internet]. BioMed Central; 2016 [cited 2019 Oct 27];7:11. Available from: http://evodevojournal.biomedcentral.com/articles/10.1186/s13227-016-0048-4

69. Fonseca RR, Couto A, Machado AM, Brejova B, Albertin CB, Silva F, et al. Supporting data for "A draft genome sequence of the elusive giant squid, Architeuthis dux" GigaScience Database 2019. http://dx.doi.org/10.5524/100676

Tables

Table 1. Statistics of the giant squid genome assembly (Meraculous + Dovetail) and corresponding gene prediction and functional annotation. The transcript evidence was confirmed by blastp hits with e-value < $10E^{-6}$ using the transcriptomes of three other species of squid (see the "Transcriptome sequencing" section).

**Global Statistics**		
**Genome assembly***	**Genome**	**Gene models with evidence**
Input assembly	Meraculous	
Contig N50 length (Mb)	0.005	
Longest contig (Mb)	0.120	
Scaffold N50 length (Mb)	4.852	
Longest scaffold (Mb)	32.889	
Total length (Gb)	2.693	
**BUSCO statistics ([1]Euk / [2]Met)**		
Complete BUSCOs, (%)	86.1 / 88.5	81.6 / 78.3
Complete and single-copy, (%)	85.1 / 87.6	79.9 / 77.7
Complete and duplicated, (%)	1.0 / 0.9	1.7 / 0.6
Partial, (%)	4.3 / 3.6	9.6 / 5.7
Missing, (%)	9.6 / 7.9	8.8 / 16.0
Total Buscos found, (%)	90.4 / 92.1	91.2 / 84.0
**Genome annotation / Gene Prediction**		
Protein-coding gene number	33,406	
Transcript evidence	30,472	
Average Protein length, (aa)	339	
Longest Protein, (aa)	17,047	
Average CDS length, (bp)	1,015	
Longest CDS, (bp)	51,138	

Average exon length, (bp)	199
Average exons per gene	5
**Functional annotation (Number of Hits)**	
Swissprot	15,749
Uniref90	29,553
GO Terms	4,712
Conserved Domains Database (CDD)	15,280

*****The presented statistics are to contigs/scaffolds with length >= 500 bp.

[1]Euk: Database of Eukaryota orthologs genes, containing a total of 303 BUSCO groups.

[2]Met: Database of Metazoa orthologs genes, containing a total of 978 BUSCO groups.

Figure legends

Figure 1. Comparison of genome repeat content among available cephalopod genomes with assembled genomes (repeat data for *O. minor* and *O. bimaculoides* from [51] and for *E. scolopes* from [52]). The tree indicates evolutionary relationships among the two available octopod cephalopods and the two available decapod cephalopods. Pie charts are scaled according to genome size (*O. bimaculoides*: 2.7Gb, *O. minor*: 5.09Gb, *E. scolopes*: 5.1Gb, *A. dux*: 2.7Gb), with different repeat types indicated by the colours presented in the key.

Figure 2. A) Stacked bar chart illustrating the proportions (expressed as percentage of the total genome) of repeats found in genic (≤2kb from an annotated gene) and intergenic regions (>2kb from an annotated gene) for the giant squid genome. **B)** Transposable element (TE) accumulation history in the giant squid genome, based on a Kimura distance-based copy divergence analysis of TEs, with Kimura substitution level (CpG adjusted) illustrated on the x-axis, and percentage of the genome represented by each repeat type on the y-axis. Repeat type is indicated by the colour chart below the x-axis.

Figure 3. Schematic representation of the Hox gene clusters. Different scaffolds are separated by two slashes. **A)** Simplified classification of the Hox clusters genomic organisation. Type A identifies the lack of a “typical” Hox cluster configuration, i.e. genes are scattered through the genome (not closely placed); Type S indicates a Hox cluster that is separated by a chromosomal breakpoint; Type D clusters comprehend all the genes in the same location but encompassing a larger region than in organised clusters and may display non-Hox genes and repeats in between; Type O indicates a very compact cluster embracing a short region with only Hox genes. Non-coding RNA and miRNA can be found. **B)** Simplified scheme of the chromosomal organisation in various invertebrates. Scaffold length is shown underneath. Unlike in other coleoids, for *Architeuthis dux* all Hox genes were found in the same scaffold. However, the distance between the genes was larger than expected for invertebrate organisms, and

non-homeobox genes were also present within the cluster. Hox2 remains undetected in coleoids. *A. dux* cluster can be found in scaffold25. *E. scolopes*, *O. bimaculoides*, *L. gigantea*, *C. teleta* and *D. melanogaster* assemblies and Hox cluster details can be found in [11,52,58,68]. (*) This gene was reported in a different scaffold, adjacent to non-Hox genes (the length corresponds to the size of the gene). **C)** Complete representation of the Hox cluster found in *A. dux* including the non-Hox genes. PO – Predicted open reading frame; TATDN2 – Putative deoxyribonuclease TATDN2; ZMYM1 – Zinc finger MYM-type protein 1; POGK – Pogo transposable element with KRAB; Zinc finger – Zinc finger protein; MYB-like – Putative Myb-like DNA-binding domain protein; MAPRE1 – Microtubule-associated protein RP/EB family member 1; MGC12965 – Similar to Cytochrome c, somatic.

Click here to access/download;Figure;Figure_1.pdf

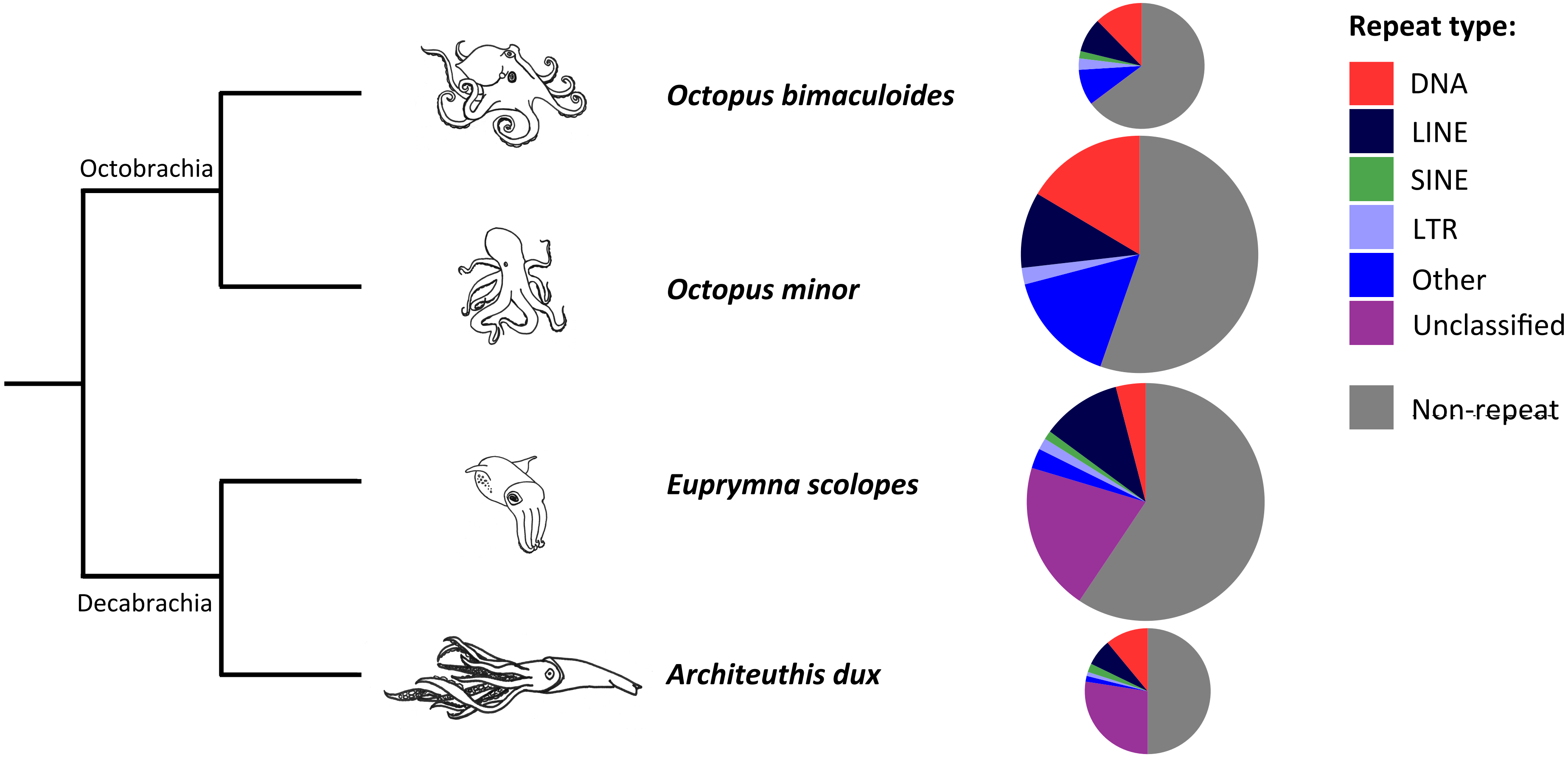

Figure 2

Click here to access/download;Figure;Figure_2.pdf

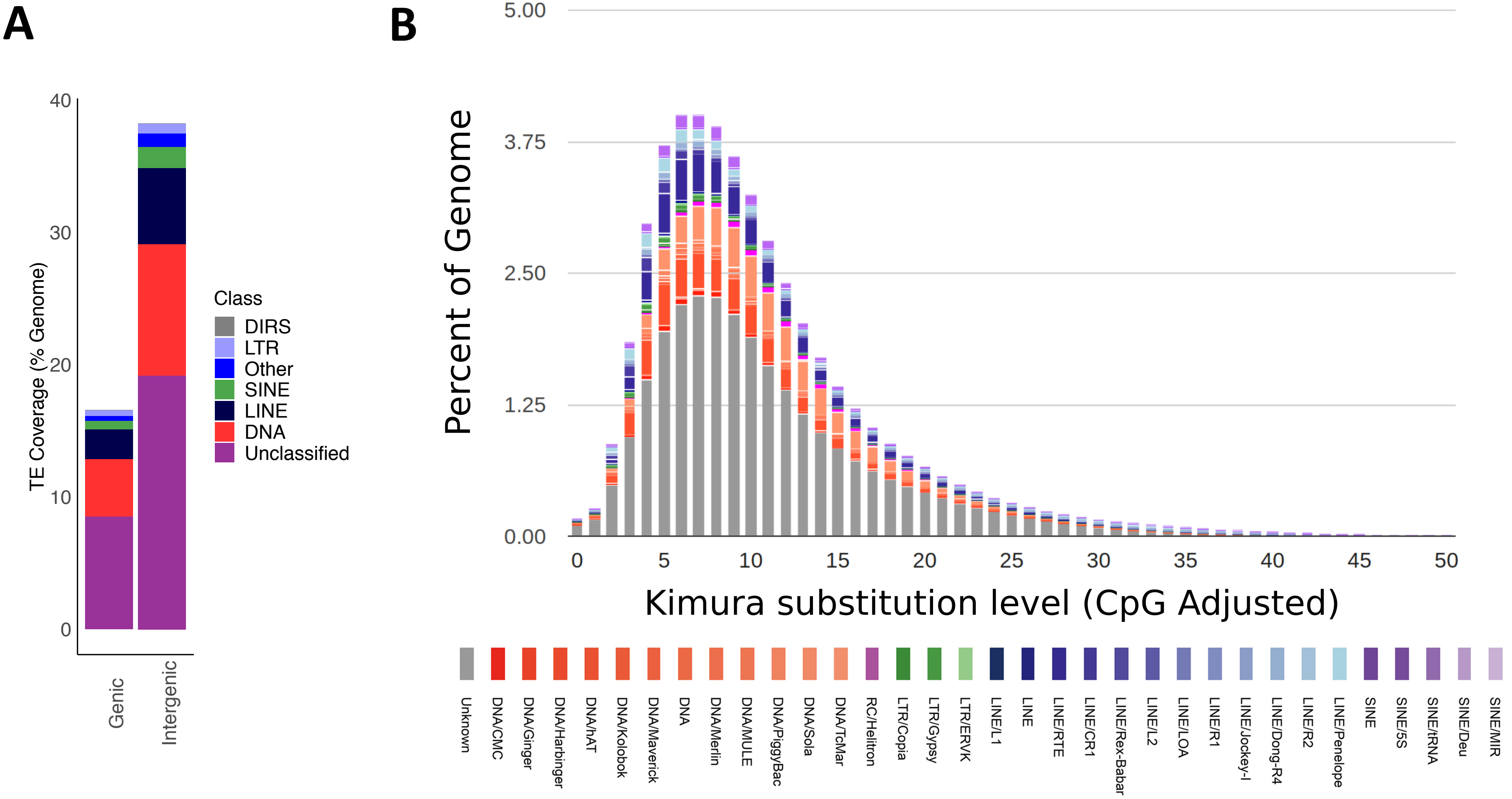

Figure 3

Click here to access/download;Figure;Fig3-HoxImg7b.pdf

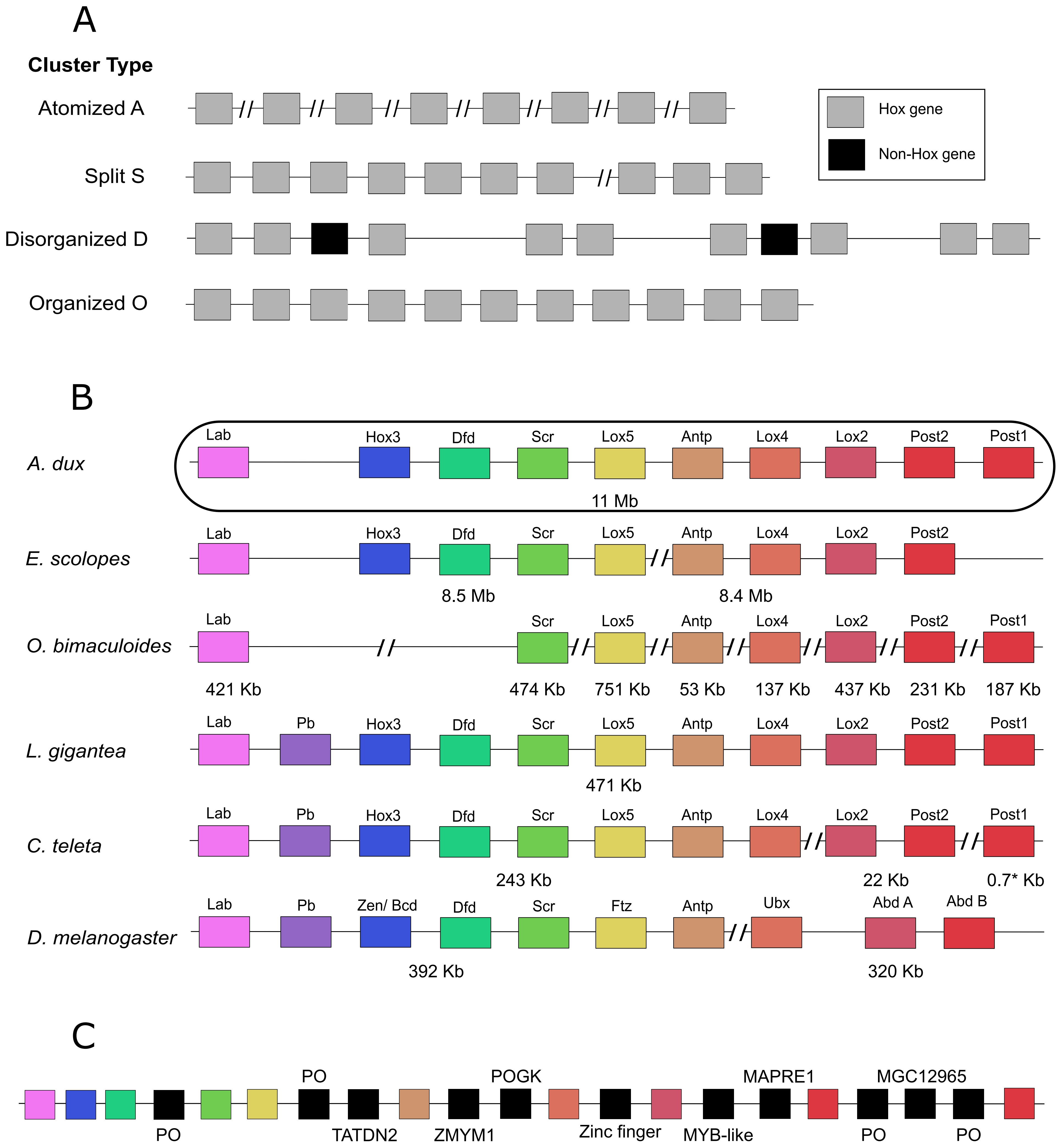

Click here to access/download
Supplementary Material
RFonseca_supplement_RF1.docx

Personal Cover
Click here to access/download;Personal Cover;replyToReviewers_rev3.docx

Dear Editor,

We herewith submit our revised manuscript 'A draft genome sequence of the elusive giant squid, *Architeuthis dux*'. We have edited the manuscript to clarify the issues raised by you and Reviewer #2, uploaded the files with the filtered annotations to the GigaScience server and updated the README file accordingly. Please find the answers to all comments below.

Rute Fonseca on behalf of all the authors.

Editor's comments:

Reviewer 2 is still concerned regarding the uncertainty of the gene models and says that, ideally, transcriptome data should be used to address this. The reviewer and I are aware that this may not be possible in this species. In this case, I agree with the reviewer that a good way forward would be to provide both, the original and the filtered versions, and discuss the uncertainties around the gene models in the paper.

We now make it clearer that transcriptomes of closely related squid species were used to guide the annotation process (since it is impossible to get that type of data from a giant squid). We do provide the two sets of annotations and extended our discussion regarding the gene models in the main text (added information from Lines 252 to 278).

Reviewer reports:
Reviewer #2: The authors have addressed most of my comments. However, I am still cautious about their gene model prediction. Running gene prediction using parameters from other species, especially Drosophila usually gives rise to very inaccurate results. The best situation would be using the transcriptome from the same species to train the gene model predictor. I understand there might be a technical limitation, but applying a random filter threshold to reduce the numbers of gene models is also problematic. This filtering may remove lineage-specific genes (i.e., novel genes in this species) and neural peptide genes that are usually very short. If having a good gene model is not possible, I would recommend the authors providing both versions of their gene models (i.e., original and filtered). And the authors should address this weakness in their manuscript.

Please note that the model parameters that were used for the final gene prediction were *A. dux* specific, they were definitely not *D. melanogaster* parameters. *D. melanogaster* parameters were used only as a starting point in the iterative process that has been guided, among other things, by RNA-seqs and proteomes from closely-related oegopsid squid species (unfortunately, we cannot obtain RNA-seq from *A. dux* due to difficulties of obtaining RNA from long-dead specimens). The RNA-seq and proteome information has also been used in the final stage of gene predictions. In this setup, the gene finder can adapt to new species (even species distant from the original parameters) and can give predictions that is in high concordance with related RNA-seq / proteome information where such information is available, while still predicting novel genes in the areas not covered by such evidence.

Methodology of iterative adaptation of gene finding parameters to new species has been previously rigorously evaluated by us (see reference [32] in the paper) as well as others (see e.g. Korf 2004, Lomsadze et al. 2005) and has been confirmed to lead to fast adaptation of the parameters to new species. **We have made additional changes to the text describing gene finding to make this more apparent.**

As to the high number of gene predictions, we think that this is mostly artefact of low contiguity of the assembly (lots of sequencing gaps) that leads to shorter gene models. (This issue is already discussed in the paper.) You are, of course, correct in pointing out that filtering for “supported” genes may lead to exclusion of truly novel genes. **Based on your suggestion, we now provide both original and filtered data sets of gene models.**

We base the downstream functional analysis on the filtered gene set, which is done based on sequence similarity to transcriptomes and proteomes of related species (not based on a length cutoff). Note that we are unable to assign putative functional characterization to genes without any additional evidence, since such assignment is done based mostly on sequence similarity. Thus, genes that were filtered out are unlikely to affect downstream analysis in significant ways, yet **we agree that they may be a useful resource for other subsequent studies**.

Please note the added information within the text extending from line 252 to line 278, which includes the extra references (below for details).

Korf I. Gene finding in novel genomes. BMC bioinformatics. 2004 Dec;5(1):59.

Lomsadze A, Ter-Hovhannisyan V, Chernoff YO, Borodovsky M. Gene identification in novel eukaryotic genomes by self-training algorithm, Nucleic Acids Res. , 2005, vol. 33 (pg. 6494-6496)

Minor comments:

Lines 261-262: "Drosophila melanogaster" -> use italic type

Done.

Line 265: "A. dux" -> use italic type
Done.

Line 266: "A. dux" -> use italic type

Done.
