# Supplementary material for: A draft genome sequence of the elusive giant squid, Architeuthis dux
Source: Gigascience. 2020 Jan 16;9(1):giz152. doi: 10.1093/gigascience/giz152 (PMC6962438; doi:10.1093/gigascience/giz152)
Supplement: giz152_Supplemental_File [file giz152_supplemental_file.docx]

## Supplementary Methods.

###

### Details on the DNA extraction

Details from Winkelman et al ^1^: DNA extraction from cephalopods is challenging owing to high concentrations of mucopolysaccharides found in their soft tissues, which tend to co-separate with DNA during extraction, and subsequently can inhibit many downstream enzymatic processes. Furthermore, the muscular tissues of Architeuthis (as well as squid belonging to 15 other families of the order Oegopsida) naturally contain high levels of ammonia, conferring additional complications to DNA extraction ^2^. Tissue samples were initially digested at 56C with agitation, in a cetyltrimethylammonium bromide (CTAB) containing buffer (100 mM Tris–HCl, pH 8.0; 1.4 M NaCl; 20 mM EDTA; 2% w/v CTAB; 0.2% w/v dithiothreitol; 500 mg/ml proteinase K). Subsequently, the digests underwent three purifications, each of one volume of 25:24:1 phenol:chloroform:isoamyl alcohol saturated with 10 mM Tris–HCl (pH 8.0) and 1 mM EDTA. One volume of ddH2O was subsequently added to the aqueous phase in order to dilute the salt concentration, prior to precipitation of the DNA by adding one volume of 100 percent isopropanol and centrifugation at 20000 r.p.m., followed by a 70 percent ethanol wash step. The extracted DNA was eluted in 1x TE buffer pH 8.0 (10 mM Tris –HCl, pH 8.0; 1 mM EDTA). The quality of all extracts was assessed by electrophoresis on 0.7 percent agarose gels with ethidium bromide, as well as by fluorometric quantitation on the Qubit1.0 fluorometer (Invitrogen, Carlsbad, CA, USA).

### Proteomics

#### Proteomics: MALDI‑TOF/TOF and Protein Identification

MALDI-TOF/TOF analysis was performed according to ^3^ with modifications. Peptide samples were concentrated in a Centrivap (Labconco) and re-suspended in trifluoroacetic acid 0.1% (v/v). Samples were concentrated and cleaned according to the manufacturer’s instructions on a micro C18 column (ZipTip, Millipore, Bedford, MA, USA). The proteins were eluted in 1.7 µL directly onto the MALDI plate using the matrix α-cyano-4-hydroxycinnamic acid (α-CHCA) at 7 mg/mL prepared in ACN (50%), trifluoroacetic acid (0.1%) and ammonium phosphate (6 mM). Peptide mass spectrometry analyses were performed by MALDI-TOF/TOF (4700 Proteomics Analyzer MALDI-TOF/ TOF, SCIEX, Framingham, MA, USA) in reflector positive mode (700–4000 Da). The experimental mass spectra were searched against the UniprotKB protein sequence database with the MASCOT (Matrix-Science, London, UK) algorithm, integrated in the GPS Explorer software (AB SCIEX, Framingham, MA, USA). Moreover, the peek list was also searched against the NCBI (Metazoa, Mollusca). The search parameters were up to two maximum trypsin missed cleavages, mass tolerance of 50 ppm, cysteine carbamidomethylation (fixed modification), methionine oxidation (variable modification) and a charge state of +1. For the sequences that gave uncharacterized, predicted or putative proteins, a blast search was performed in the NCBI to obtain more information by homology. Searches on PFam ^4^ were done for one entry, and for the rest of the proteins we the molecular function, biological process and cellular component were obtained from the Gene Ontology (GO) ^5^ in UniprotKb ^6^.

### Transposable element annotation from raw reads

#### Repeat assembly from raw reads with REPdenovo

A repeat library was generated with REPdenovo ^7^. Briefly, raw reads from 500bp insert libraries were cut to kmers (by default 19mer, 29mer, and 39mer are used) and the kmer frequency was assessed. Then, high frequency kmers (by default 10 times the average coverage as cutoff) were collected and used as input of the repeats assembly step. A two stage assembly step is applied: firstly, raw contigs are assembled from the high frequency kmers with a genome assembler (by default velvet), and then an overlap-graph based approach is used to merge the raw contigs to longer contigs. Raw reads are then aligned to the merged contigs to scaffold them, after which the scaffolded contigs are used as the final assembled repeats. Also, based on the number of aligned reads , repeat copy number is estimated for each assembled repeat.

#### Repeat assembly from raw reads with ReAS

We subsampled trimmed Illumina reads and applied ReAS ^8^ with parameters “-d 100 -m 80 -size 50 -min_extend 5 -max_extend 50” to select reads that were corresponding to repetitive sequences over their full length. Those reads were then assembled with Trinity and annotated by similarity to known repeat elements in RepeatMasker, as well as BLAST similarity to a custom library of repeats using an in-house pipeline. After further filtering of unassembled sequences this library contained 1444 sequences.

## Supplementary Figures

**Figure S1.** Photographs (by Ricardo Tafur) depicting the three squid specimens for which we generated transcriptomes and information regarding the specimen collection.

| 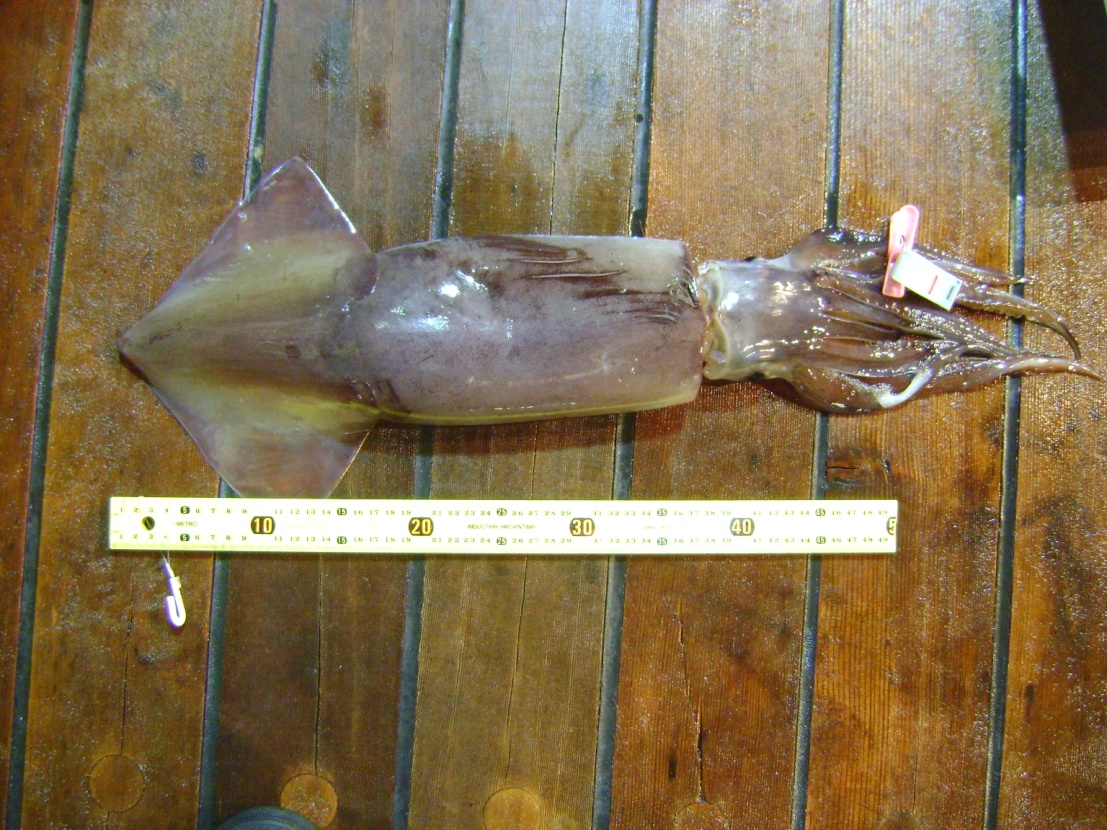 | *Dosidicus gigas*  LM: 206 mm  TW: 220 g  Sx: ♂ | Date: 22/12/11  Lat: 3º59.9’ S  Long: 86º0.8’ W  METHOD OF CAPTURE: JIGGING |
| --- | --- | --- |
| 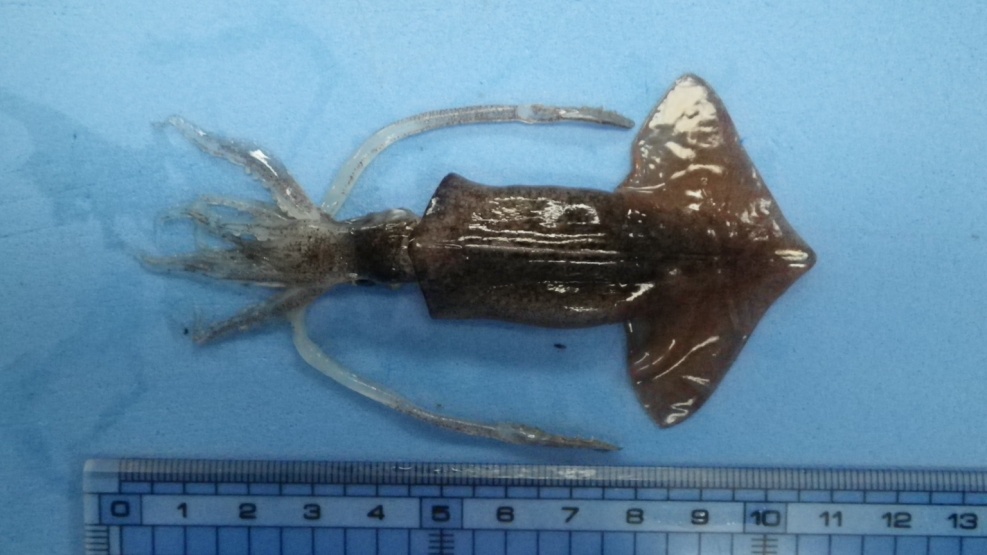 | *Onychoteuthis sp. Aff. Banksia*  LM: 61 mm  TW: 9 g | Date: 9/01/12  Lat: 13º59.46’ S  Long: 80º00.03’ W  METHOD OF CAPTURE:  MIDWATER TRAWL NET NICHIMO LC-100 m^2^ – R3 (LC) |
| 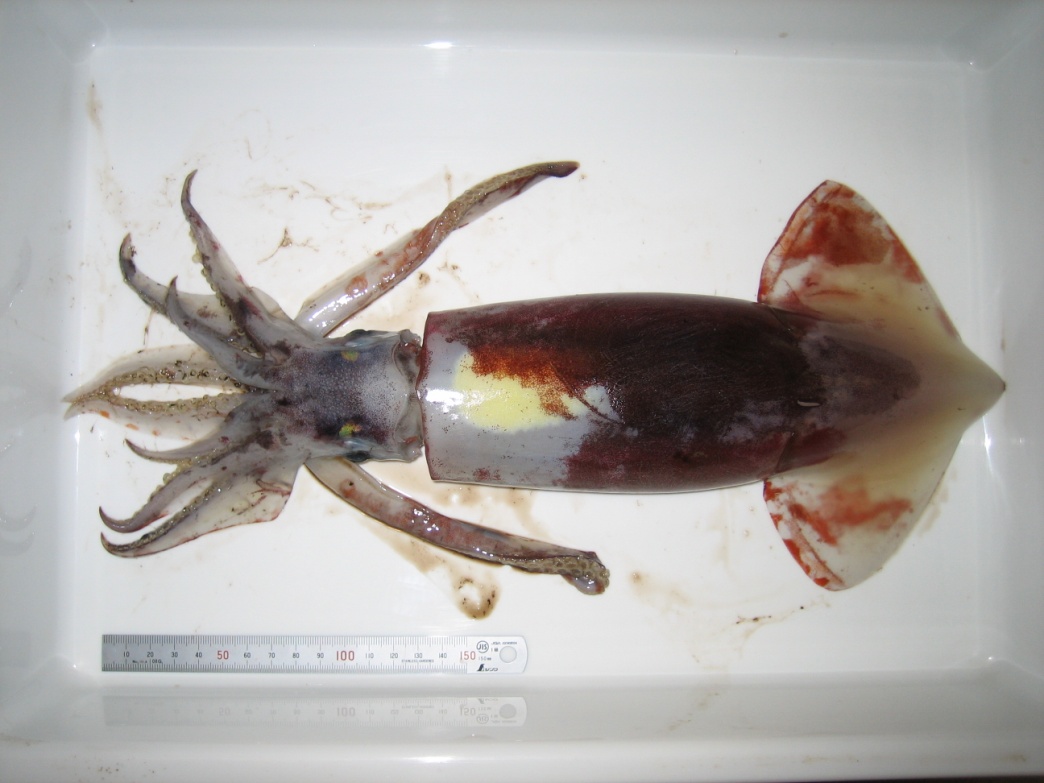 | *Sthenoteuthis oualaniensis*  LM: 250 mm  TW: 631.5 g  Sx: ♀ | Date: 24/12/11  Lat: 5º59.9’ S  Long: 85º06.94’ W  METHOD OF CAPTURE: JIGGING |

##
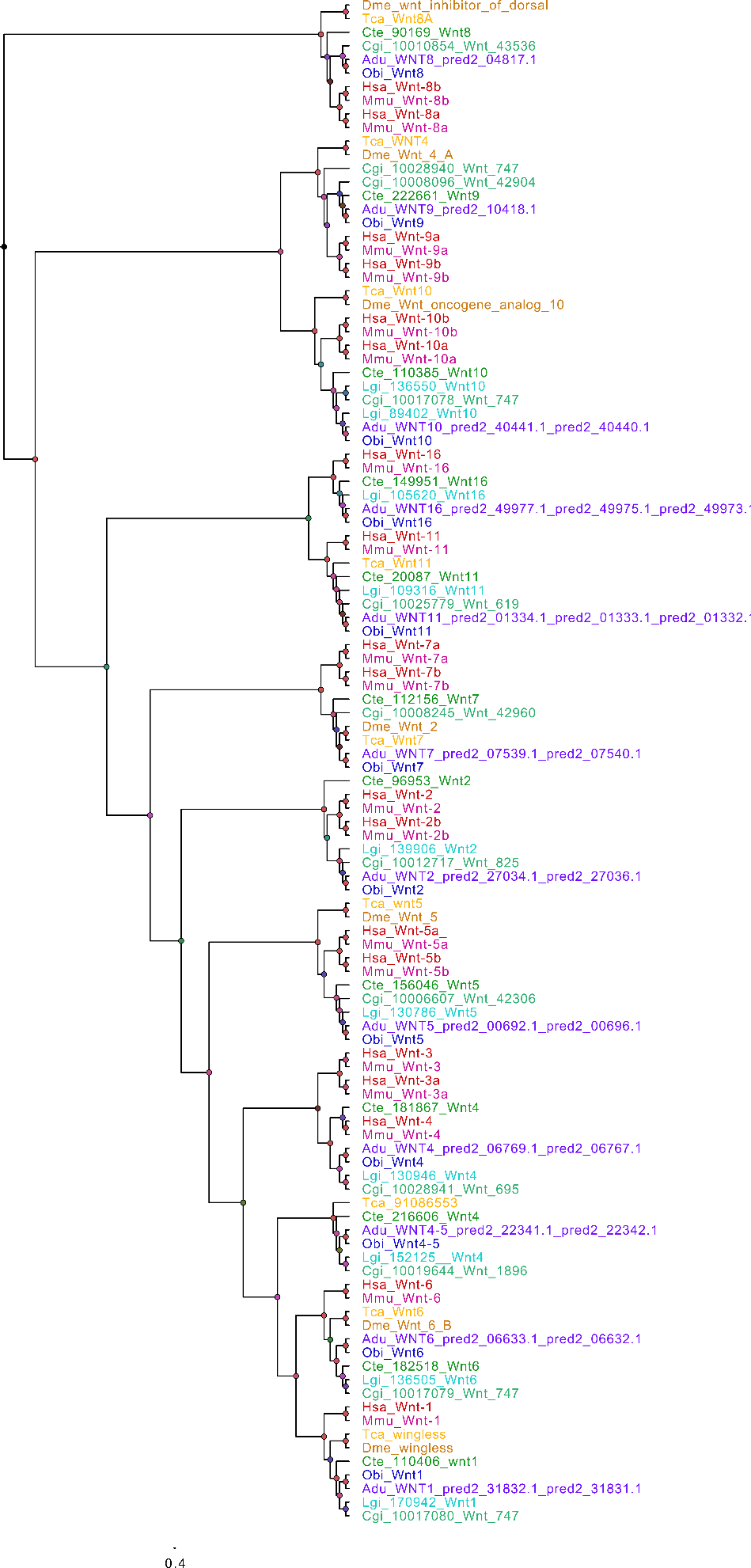


**Figure S2.** Phylogenetic tree obtained with FastTree ^9^ showing the relationship between WNTs across Metazoa. WNT are a family of secreted lipid-modified signaling glycoproteins with a key role during development ^10^. The giant squid has the typical 12 lophotrochozoan WNTs (1, 2, 4, A, 5, 6, 7, 8, 9, 10, 11 and 16). The tips are colored by species, and the names correspond to abbreviated Genus and species names. *Architeuthis dux* (Adu, purple), *Octopus bimaculoides* (Obi, dark blue), *Lottia gigantea* (Lgi, teal), *Crassostrea gigas* (Cgi, sky blue), *Capitella teleta* (Cte, green), *Mus musculus* (Mmu, pink), *Homo sapiens* (Hsa, red), *Tribolium castaneum* (Tca, burnt orange), *Drosophila melanogaster* (Dme, orange).


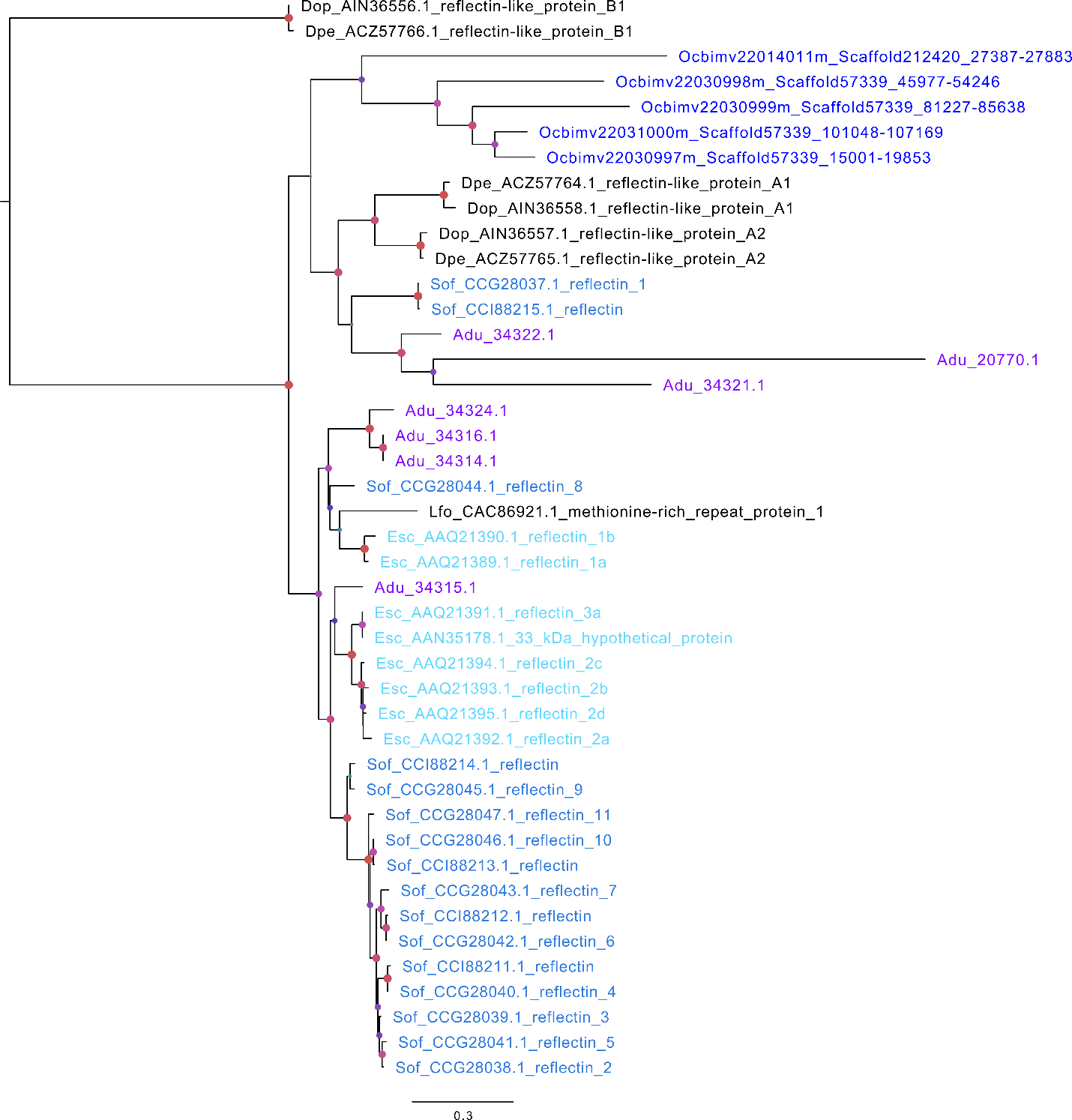


**Figure S3.** Phylogenetic tree showing the relationship between reflectin genes across cephalopods, with *Octopus bimaculoides* (Obi, dark blue) representing class Octopoda, cuttlefishes being represented by *Sepia officinalis* (Sof, blue) and *Euprymna scolopes* (Esc, sky blue), and including three species of squid *(Loligo forbesii* (Lfo), *Doryteuthis pealeii* (Dpe), *Doryteuthis opalescens* (Dop)) besides the giant squid (*Architeuthis dux* (Adu, purple)).


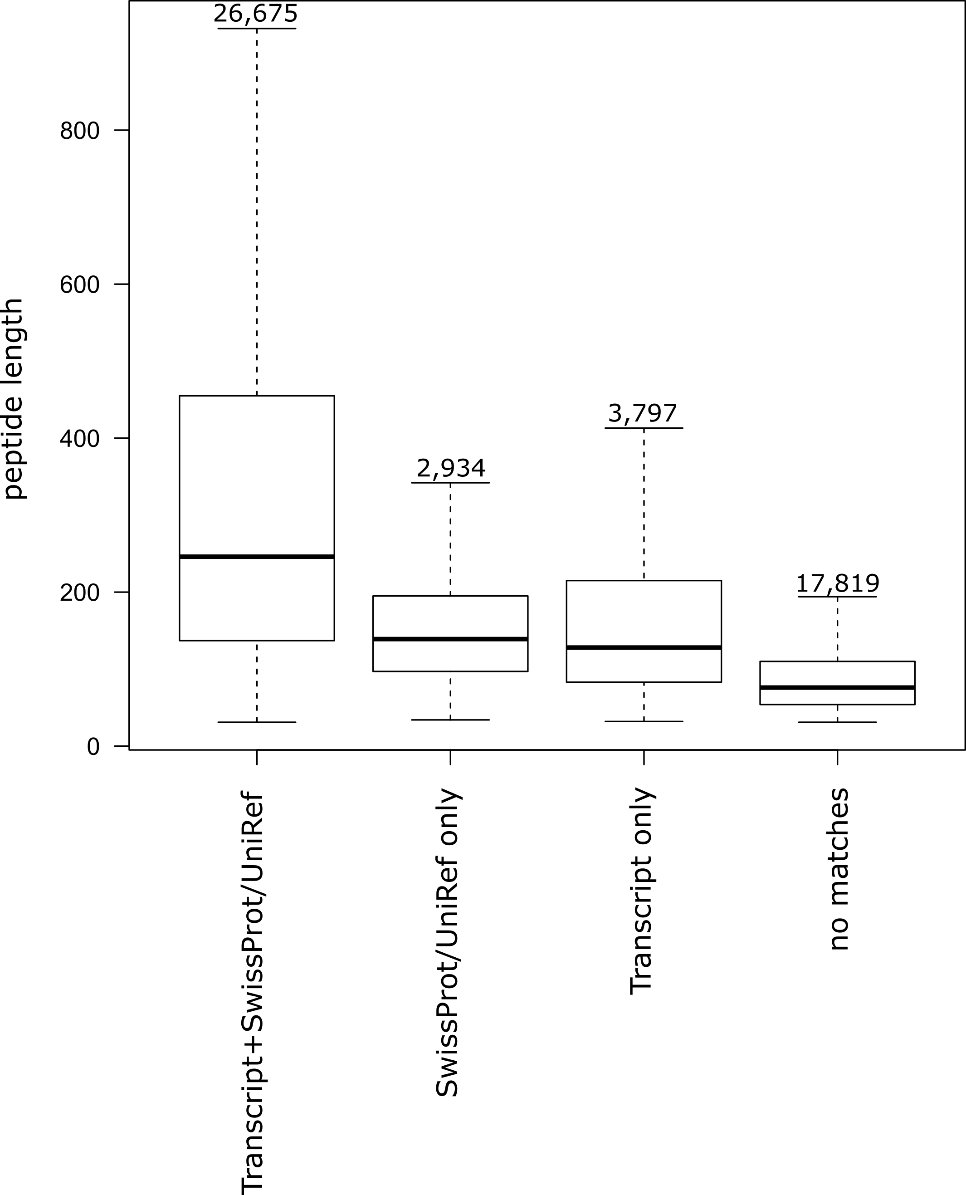


**Figure S4.** Size distribution of the 51,225 sequences initially annotated as protein-coding. 33,406 sequences were found to have transcript evidence (blastp match to a sequence from a cephalopod transcriptome, with at least 50% of the giant squid coding region covered) and/or matches in Swissprot or UniRef90 databases (Table 1). The remaining 17,819 sequences were found to be fragmentary and were discarded from the main annotation set.

## Supplementary Tables

**Table S1.** Summary of the sequence data used in this project.

| Type | Notes | Number of reads | Coverage (X) | Total (Gb) |
| --- | --- | --- | --- | --- |
| Illumina (170bp insert) | 6 libraries | 1,166,547,216 | 116 | 116 |
| Illumina (500bp insert) | 3 libraries | 382,595,608 | 38 | 38 |
| Illumina (800bp insert) | 3 libraries | 380,827,014 | 38 | 38 |
| Illumina (5 kb insert) | 1 libraries | 54,929,603 | 2 | 5.4 |
| MOLECULO | 7 libraries | 1,292,292 | 1.4 | 3.8 |
| Chicago libraries | 2 libraries |  | 40 | 108.0 |
| PACBIO | 19 smrtcells |  | 10 | 27.0 |

Table S2. Statistics of the additional Giant Squid genome assemblies produced by this study.

| **Global Statistics** | **Meraculous** | **Meraculous + Dovetail + Pacbio** |
| --- | --- | --- |
| **Genome assembly*** |  |  |
| Input assembly | - | Meraculous + Dovetail |
| Contig N50 length (Mb) | 0.005 | 0.009 |
| Longest contig (Mb) | 0.120 | 0.166 |
| Scaffold N50 length (Mb) | 0.032 | 5.478 |
| Longest scaffold (Mb) | 0.683 | 38.303 |
| Total length (Gb) | 2.673 | 3.155 |
| **Busco Statistics (^1^Euk / ^2^Met)** |  |  |
| Complete BUSCOs (%) | 74.3 / 79.1 | 85.5 / 88.9 |
| Complete and single-copy (%) | 73.6 / 78.3 | 84.5 / 88.0 |
| Complete and duplicated (%) | 0.7 / 0.8 | 1.0 / 0.9 |
| Partial (%) | 13.5 / 10.4 | 5.0 / 3.0 |
| Missing (%) | 12.2 / 10.5 | 9.5 / 8.1 |
| Total Buscos found (%) | 87.8 / 89,4 | 90.5 / 91.9 |

**Table S3.** The number of genomic copy numbers of the different transfer RNA isotypes predicted by tRNAscan-SE version 1.3.1 and Rfam v. 12.0 with the Infernal v. 1.1. The Rfam predictions that did not overlap with tRNAscan predictions are in parentheses.

| Isotype | Num | Isotype | Num | Isotype | Num | Isotype | Num | Isotype | Num |
| --- | --- | --- | --- | --- | --- | --- | --- | --- | --- |
| Ala | 322 | Gln | 129 | Leu | 198 | Ser | 186 | Val | 3203 |
| Arg | 202 | Glu | 110 | Lys | 85 | Sup | 42 | Pseudo | 7996 |
| Asn | 224 | Gly | 123 | Met | 334 | Thr | 284 | SeC | 51(+23) |
| Asp | 47 | His | 55 | Phe | 65 | Trp | 61 | Undet | 402 |
| Cys | 77 | Ile | 178 | Pro | 106 | Tyr | 860 | Rfam | 8328 |

**Table S4.** Summary of repeat types present in the genome of the giant squid (*Architeuthis dux*).

| **Summary Genome Information** | **Meraculous + Dovetail** |
| --- | --- |
| Sequences in Query Fasta | 7,376 |
| Total Genome Length (bp) | 2,693,623,328 (2,242,358,460 excl N/X-runs) |
| GC level | 36.10% |
| Bases masked | 1,324,572,421 (49.17%) |

| **Class/Family** | **Number of Elements** | **Length Occupied (bp)** | **Percentage of Sequence (%)** |
| --- | --- | --- | --- |
| **SINEs** | 354,924 | 53,472,360 | 1.99 |
| ALUs | 0 | 0 | 0.00 |
| MIRs | 0 | 0 | 0.00 |
| **LINEs** | 766,382 | 187,367,738 | 6.96 |
| LINE1 | 26,666 | 4,980,003 | 0.18 |
| LINE2 | 25,624 | 4,603,689 | 0.17 |
| L3/CR1 | 71,358 | 19,166,237 | 0.71 |
| **LTR elements** | 90,553 | 19,503,842 | 0.72 |
| ERVL | 0 | 0 | 0.00 |
| ERVL-MaLRs | 0 | 0 | 0.00 |
| ERV_classI | 9,073 | 1,403,201 | 0.05 |
| ERV_classII | 867 | 207,457 | 0.01 |
| **DNA elements** | 1,626,482 | 298,004,768 | 11.06 |
| hAT-Charlie | 215,409 | 42,311,790 | 1.57 |
| TcMar-Tigger | 173,131 | 29,451,995 | 1.09 |
| **Unclassified** | 4,556,131 | 740,142,866 | 27.48 |
| **Total interspersed repeats** | 0 | 1,298,491,574 | 48.21 |
| Small RNA | 168,903 | 24,891,018 | 0.87 |
| Satellites | 118,384 | 564,234 | 0.02 |
| Simple Repeats | 4,395 | 0 | 0.00 |
| Low complexity | 0 | 0 | 0.00 |

## References

1. Winkelmann I, Campos PF, Strugnell J, et al. Mitochondrial genome diversity and population structure of the giant squid Architeuthis: genetics sheds new light on one of the most enigmatic marine species. *Proceedings Biol Sci*. 2013;280(1759):20130273. doi:10.1098/rspb.2013.0273.

2. Voight JR, Pörtner HO, O’Dor RK. A review of ammonia‐mediated buoyancy in squids (cephalopoda: Teuthoidea). *Mar Freshw Behav Physiol*. 1995;25(1-3):193-203. doi:10.1080/10236249409378917.

3. Frazão B, Campos A, Osório H, et al. Analysis of Pelagia noctiluca proteome Reveals a Red Fluorescent Protein, a Zinc Metalloproteinase and a Peroxiredoxin. *Protein J*. 2017;36(2):77-97. doi:10.1007/s10930-017-9695-0.

4. Finn RD, Tate J, Mistry J, et al. The Pfam protein families database. *Nucleic Acids Res*. 2008;36(Database issue):D281-8. doi:10.1093/nar/gkm960.

5. Ashburner M, Ball CA, Blake JA, et al. Gene Ontology: tool for the unification of biology. *Nat Genet*. 2000;25(1):25-29. http://dx.doi.org/10.1038/75556.

6. Boutet E, Lieberherr D, Tognolli M, et al. UniProtKB/Swiss-Prot, the Manually Annotated Section of the UniProt KnowledgeBase: How to Use the Entry View. In: *Methods in Molecular Biology (Clifton, N.J.)*. Vol 1374. ; 2016:23-54. doi:10.1007/978-1-4939-3167-5_2.

7. Chu C, Nielsen R, Wu Y. REPdenovo: Inferring De Novo Repeat Motifs from Short Sequence Reads. Antoniewski C, ed. *PLoS One*. 2016;11(3):e0150719. doi:10.1371/journal.pone.0150719.

8. Li R, Ye J, Li S, et al. ReAS: Recovery of Ancestral Sequences for Transposable Elements from the Unassembled Reads of a Whole Genome Shotgun. *PLoS Comput Biol*. 2005;1(4):e43. doi:10.1371/journal.pcbi.0010043.

9. Price MN, Dehal PS, Arkin AP. FastTree: computing large minimum evolution trees with profiles instead of a distance matrix. *Mol Biol Evol*. 2009;26(7):1641-1650. doi:10.1093/molbev/msp077.

10. Cadigan KM, Nusse R. Wnt signaling: a common theme in animal development. *Genes Dev*. 1997;11(24):3286-3305. doi:10.1101/GAD.11.24.3286.
