# Supplementary material for: A draft genome sequence of the elusive giant squid, Architeuthis dux
Source: Gigascience. 2020 Jan 16;9(1):giz152. doi: 10.1093/gigascience/giz152 (PMC6962438; doi:10.1093/gigascience/giz152)
Supplement: giz152_Response_to_Reviewer_Comments_Original_Submission [file giz152_response_to_reviewer_comments_original_submission.pdf]

# Author's Response To Reviewer Comments

Close

Dear Editor,

We herewith submit our revised manuscript ‘A draft genome sequence of the elusive giant squid, Architeuthis dux’.

Regarding the points that you have highlighted, please find the answers below:

1) Please clarify the rationale for the unconventional assembly strategy in the revised manuscript. If this has "historic" rather than scientific reasons, the reviewer feels this may be fine, but I agree that the reasons should be discussed in the manuscript, for the benefit of readers who are looking for best practice examples.

The reviewer is correct that there is some degree of history involved. We initially did the assembly without PacBio, and did the presented analyses on this. Later we were offered the chance to try and improve it with PacBio, which we did, but as you can see there was minimal improvement in the assembly statistics (Table 1 and Table S2), but i) an increase of the total genome size to 3.155 Gb, beyond the expected 2.7 Gb estimated in kmergenie, and ii) a slight decrease in the BUSCO completeness assessment. As such, we elected to retain the results based on the original assembly (based on Dovetail), but given that we assume others may wish to use the alternative assembly and explore the differences, we provide both.
In the beginning of the “Data analyses” section, we now clearly state which assembly was used in the comparative genomics analyses (from Line 297) and provide an explanation for that choice.

2) Please expand on the methods for protein-coding gene modelling and have another look at your data whether 50K genes may be an overestimate. I also agree with the reviewer's recommendation to analyze gene models in BUSCO to give readers a better idea of their completeness.

We now expanded the section detailing the filtering of the protein-coding gene set and present a total of 33,406 gene annotations in the final set, as these have validation by matching to cephalopod transcripts and/or SwissProt/UniRef90 proteins. We also provide the results from BUSCO when using the gene models as input for comparison (added to Table 1).

Answers to the reviewer’s comments:

Reviewer #1: In this study, de Fonseca et al. report the genome of the giant squid as a resource to investigate the unique traits of this fascinating organism. Two assemblies, which are of comparable contiguity to most other recently published molluscan genomes, as well as a set of over 51,000 gene models are reported. Analysis of the genome focuses on repetitive elements (e.g., TEs), non-coding RNAs, and gene families of interest to the authors (WNT genes, Protocadherins, Hox genes, and reflectins). Overall this is a straightforward study that provides a resource that will be broadly useful and I feel it should be published. However, I have a number of suggestions for improvement including a few important issues that need to be addressed.

Major points:

1.1. It is unclear why two different genome assemblies are presented instead of just one most optimal assembly. This is not the way I would have gone about assembling this combination of data but presumably Dovetail scaffolding and gene modelling were performed before PacBio sequencing and scaffolding? Re-doing the assembly would a more logical way would probably have relatively little improvement but a little more explanation of the rationale or 'historical' reasons for two different assemblies and/or this assembly strategy would be a helpful addition to readers looking in the literature

for examples on best practices for genome assembly.

Thank you for this comment. The reviewer is correct that there is some degree of history involved. We initially did the assembly without PacBio, and did the presented analyses on this. Later we were offered the chance to try and improve it with PacBio, which we did, but as you can see there was minimal improvement in the assembly statistics (Table 1 and Table S2), but i) an increase of the total genome size to 3.155 Gb, beyond the expected 2.7 Gb estimated in kmergenie, and ii) a slight decrease in the BUSCO completeness assessment. As such, we elected to retain the results based on the Dovetail assembly, but given that we assume others may wish to use the alternative assembly and explore the differences, we provide both.

1.2. Related to this issue, there is little comparison of the two genome assemblies and it is unclear which assembly was used for what analyses and even Table 1 and Table S2's titles are a bit ambiguous with respect to which assembly statistics are presented. Please explicitly state which assembly was used for which analyses.

In the beginning of the "Data analyses" section, we now clearly state which assembly was used in the comparative genomics analyses (from Line 297) and provide an explanation for that choice. Additionally, we also mention the choice in the Methods section (Lines 183 to 185) before describing the strategies for annotation and comparative analyses.

1.3. The approach used for gene annotation is unconventional and the inferred number of protein-coding gene models is very high. This does not mean the gene model set is bad, but I feel that data needed for the reader to assess the quality of the gene models are lacking. Please run BUSCO on the gene models and report these data as well.

We now also provide the results from BUSCO when using the gene models as input for comparison.

1.4. Specimen collection data are not reported in the manuscript.

This information has now been added to Table S1.

Minor points:

1.5. Scientific names of species need to be italicized throughout.

Done.

1.6. Did all the giant squid DNA come from the same individual?

Yes, this is now clear in Line 172.

1.7. Lines 140-141: "currently increasing locally" is a bit awkward and vague.

Replaced by "in some regions".

1.8. Line 176: Which reads? All Illumina reads? PE reads only?

This has now been clarified on Line 176.

1.9. Line 185: Again, this seems to me to be a strange assembly strategy and I think that it should be clearly stated that PacBio data became available 'late in the game' if that is the case. Otherwise, the logic behind this assembly strategy needs to be explained.

In the beginning of the "Data analyses" section, we now clearly state which assembly was used in the comparative genomics analyses (from Line 297) and provide an explanation for that choice.

1.10. Line 199: High-throughput is misspelled.

Done.

1.11. Line 203: Clarify what is meant by reference transcriptome. All reads from all tissues were pooled and assembled together?

This has now been clarified in Lines 203-204.

1.12. Line 205: "EvidencialGene" is a tyo.

Corrected.

1.13. Lines 261-262: Please provide details on exactly what was done in this study in the supplementary material. Description of how the final gene models were selected is vague.

We now further discuss the filters applied in lines 272-275. The total number of protein-coding genes passing all the filters is 33,406.

1.14. Line 277: What is meant by a "bespoke pipeline"? Custom scripts should be made available.

No custom analysis scripts were developed. We simply use ‘bespoke’ to mean 'tailored to our particular purpose'. Here this refers an analysis pipeline combining: a preliminary analysis using RepeatMasker, followed by a de novo analysis using RepeatModeler and a referenced and publicly available script by Platt et al, followed by a full annotation using RepeatMasker. These steps are fully outlined and referenced in the methods section. We have simplified the sentence which now reads:
“Repetitive elements were first identified using RepeatMasker v.4.0.8”

1.15. Line 450: Correct "Sampling was following"

Done.

1.16. BUSCO results are presented in the methods section (should be in the results by the way) for the pre-PacBio scaffolding genome but not the post-PacBio scaffolding genome.

The results of BUSCO for post-PacBio step are presented in Table S2 (as indicated in Line 186). We moved the description of the BUSCO results to the “Data analyses” section and added a clarification regarding the choice of the assembly for the overall comparative genomics analyses (from Line 297).

1.17. Table 1: BUSCO should be in all capital letters.

Done.

1.18. Figure 3: What does the note "Gene size only" mean?

This gene was reported to be fully isolated from other Hox genes in a different scaffold but was not alone in the scaffold. There were other non-Hox genes. Figure 3 aims to show both the organisation and the range occupied by Hox genes. Considering the organisation, the gene is isolated such as in O. bimaculoides. Regarding the size, the schematic representation indicates only the Hox “cluster” area. In O. bimaculoides, the scaffolds contain only the Hox genes. This means it could be possible for the cluster to be there but only when considering a very vast distance. In this scenario for C. teleta, the gene is found in the middle of the scaffold, surrounded by other genes. It is not part of the cluster. Indicating the full scaffold size could lead to a wrong interpretation of the gene size and of the Hox gene range. As such, only the gene size is indicated.

1.19. Table S1: Please provide total number of reads and somewhere it should be clarified how many different instrument runs were conducted and if different libraries were multiplexed on the Illumina platform.

This information has now been added to Table S1.

Reviewer #2: The authors present the genome of the giant squid Architeuthis dux. Several cephalopod genomes have been sequenced, but our genomic understanding of cephalopods living in the deep-sea environment is still poor. The authors sequenced a giant squid species A. dux together with several transcriptomes from the gonad, liver and brain tissues derived from three other squid species including

Onychoteuthis banksii, Dosidicus gigas, and Sthenoteuthis oualaniensis.

Having a giant squid genome is an important contribution to the field of cephalopod genomics, especially for further meaningful comparative genomics. The authors provide a decent genome assembly. And the observation of a non-tightly physically linked Hox cluster is interesting. The manuscript is well written in general, however, there are a lot of editing errors throughout the whole manuscript, which distracts the reading. The authors need to carefully fix all these typos and errors during the revision. Further comments are provided below.

Major comments:

2.1. In the Abstract/Findings, there is a lot of information about "Methods" (e.g. how many raw reads, sequencing of proteome and RNA) instead of what the authors found from the genome itself. Also, the statement "RNA from three different tissue types from three other species of squid to assist genome annotation." is very vague. What tissue types from what species should be clearly described. The authors need to rewrite this section.

In the abstract we followed the format that is usual in a data note, providing detailed information on the data provided by this work. We have now added the names of the three species of squid to the abstract.

2.2. Line 153: Body patterning system? Usage of body patterning is confusing here since body patterning often refers to the developmental process during embryogenesis but not the skin color pattern.

We have rephrased the sentence to: “Cephalopods can rapidly alter the texture, pattern, colour and brightness of their skin, and this both enables a complex communication system, as wells as provides exceptional camouflage and mimicry.”

2.3. The authors cited that there is a global proliferation of cephalopods (Lines 140 and 141) but later cited other studies saying that there is a regional extinction. It is a bit confusing whether cephalopods are undergoing proliferation or extinction. Given that the earlier citation is more recent (Doubleday et al., 2016) than others, it is wondering which condition is closer to the current situation.

We have removed the second statement to avoid confusion.

2.4. Although it is agreeable in general to have genome resources from unexplored species, the authors' argument in the last paragraph of Data description/Context is not convincing. The link between having a genome and aiding conservation efforts as well as ensuring continued existence is not clear.

Without a genome, population genomic studies that provide information regarding the genetic diversity and structure of populations becomes very challenging, with genome-wide data having to be produced from reduced-representation methods that have many biases. In this last paragraph, we state this specifically: “A genome is an important resource for future population genomics studies[…]”.

2.5. Do the authors have any idea why the genome contains so many protein-coding genes (51,225 genes predicted) in comparing to other cephalopod species usually having only 20,000-30,000 genes? For example, is it due to that A. dux has more lineage-specific genes or expansions of certain gene families?

We have revised our gene models and now further discuss the filters applied in lines 272-275. The total number of protein-coding genes passing all the filters is 33,406.

2.6. Given that genome size and polyploidy of the organisms are often correlated to increased body size (Session et al., 2016), have the authors checked if there is whole-genome duplication or polyploidy in the A. dux genome? Session et al. (2016) Genome evolution in the allotetraploid frog Xenopus laevis. Nature 538, 336-343.

We did confirm that the genome was not polyploid by testing for Hardy–Weinberg equilibrium using re-sequencing data from 32 giant squid individuals (Winkelman et al, unpublished results) and there is no evidence for an ancient duplication since we only found one intact Hox complement.

2.7. Figure 3: The authors should provide scaffold numbers for the Hox clusters from each species. Also, in most cases, Hox genes in the Hox cluster are adjacent to each other without the insertion of other non-Hox genes. If there is a special case in A. dux and E. scolopes, the authors should show the real gene arrangement on that scaffold, especially for the non-Hox genes (with brief annotation) that are in between Hox genes. This can be achieved by having an additional panel in the same figure. The authors are encouraged to show an illustration on the types of Hox gene organization in order to give the readers a better understanding of this context.

Figure 3 has received new panels. Scaffold information for A. dux was added in panel C (Figure 3-C). As the assemblies of the other species were retrieved from other studies, the readers are directed to the appropriate references for further detail. An extra panel depicting the Hox cluster organisation in more detail has been added. E. scolopes data is shown as reported in its published study. No non-Hox genes were indicated for the area covered in this representation. An additional panel with a simplified version of the various Hox "cluster" types was inserted in panel A (Figure 3-A).

Minor comments:

2.1.1. Line 149: ~2cm -> "~2 cm"
Done.

2.1.2. Line 150: 3 orders -> "three orders"
Done.

2.1.3. Line 150: Architeuthis dux -> "A. dux"
Done.

2.1.4. Lines 150 and 151: 10-12cm… 20m -> "10-12 cm… 20 m"
Done.

2.1.5. Line 152: 500kg -> "500 kg"
Done.

2.1.6. Line 171: a Architeuthis dux sample -> "an A. dux sample"
Done.

2.1.7. Line 172: What is CTAB?
CTAB = "cetyl trimethylammonium bromide"; this description has been included in the text (Line 172)

2.1.8. Line 184: For Eukaryota and Metazoa we identified… -> "For Eukaryota and Metazoa, we identified…"
Done.

2.1.9. Line 184: … 90.4 % and 92.1 %... -> "… 90.4% and 92.1%..."
Done.

2.1.10. Line 185: 23.38Gb -> "23.38 Gb"
Done.

2.1.11. Line 186: 14.79kb -> "14.79 kb"

Done.

2.1.12. "k-mer" (Line 204) or "kmer" (Line 176) to be consistent.
Chose to use "kmer".

2.1.13. Line 216: 100,000 g -> "100,000×g"
Done.

2.1.14. Lines 219 and 222: SDS-PAGE -> "SDS-PAGE" (hyphen but not en dash)
Done.

2.1.15. Line 221: Tris - HCl -> Tris-HCl (single hyphen but not en dash with spaces)

Done.

2.1.16. Line 226: LC-MS/MS analyses -> "LC-MS/MS analyses" (hyphen but not en dash)
Done.

2.1.17. Line 254: Using italic for scientific names (i.e. Octopus bimaculatus, Crassostrea gigas, and Lottia gigantea)
Done.

2.1.18. Line 260: … 200kb (total length 199Mb)… -> "… 200 kb (total length 199 Mb)…"
Done.

2.1.19. Line 290: Transposable elements -> "transposable elements"
Done.

2.1.20. Line 300: Architeuthis dux -> "A. dux"
Done.

2.1.21. Line 323: ~5-8% -> "~5¬-8%" (en dash but not hyphen for a range)
Done.

2.1.22. Line 381: Octopus bimaculoides -> "O. bimaculoides"
Done.

2.1.23. Line 383: Euprymna scolopes -> "E. scolopes" (in italic)
Done.

2.1.24. Line 395: Euprymna scolopes -> "E. scolopes"
Done.

2.1.25. Lines 397 & 398: 500 - 10,000 kb -> "500-10,000 kb" (en dash but not hyphen for a range)
Done.

2.1.26. Line 406: ... observed in Hox 1, Hox 4, ANTP, Lox 2, Lox 5, Post 1 and Post 2. Hox 1 did,... -> "... observed in Hox1, Hox4, ANTP, Lox2, Lox5, Post1 and Post2. Hox1 did,..."
Done.

2.1.27. Line 407: Hox 1 -> "Hox1"
Done.

2.1.28. Line 408: Hox 3 -> "Hox3"
Done.

2.1.29. Line 409: Lox 4 -> "Lox4"
Done.

2.1.30. Lines 410, 412 & 413: Hox 2 -> "Hox2"
Done.

2.1.31. Line 421: ... contains 7 reflectin genes and 3 reflectin-like genes… -> "... contains seven reflectin genes and three reflectin-like genes…"
Done.

2.1.32. Line 422: … exception of 1 reflectin gene, … -> "… exception of one reflectin gene, …"
Done.

2.1.33. Line 436: … (tsa)… -> "… (TSA)…"
Done.

2.1.34. Lines 647 & 657: Architeuthis dux -> "A. dux"
Done.

2.1.35. Line 659: Hox 2 -> "Hox2"
Done.

Close
