# Supplementary material for: A draft genome sequence of the elusive giant squid, Architeuthis dux
Source: Gigascience. 2020 Jan 16;9(1):giz152. doi: 10.1093/gigascience/giz152 (PMC6962438; doi:10.1093/gigascience/giz152)
Supplement: giz152_Response_to_Reviewer_Comments_Revision_1 [file giz152_response_to_reviewer_comments_revision_1.pdf]

Author's Response To Reviewer Comments

Close

Dear Editor,

We herewith submit our revised manuscript 'A draft genome sequence of the elusive giant squid, Architeuthis dux'. We have edited the manuscript to clarify the issues raised by you and Reviewer #2, uploaded the files with the filtered annotations to the GigaScience server and updated the README file accordingly. Please find the answers to all comments below.

Best regards,
Rute Fonseca on behalf of all the authors.

####################

Editor's comments:

Reviewer 2 is still concerned regarding the uncertainty of the gene models and says that, ideally, transcriptome data should be used to address this. The reviewer and I are aware that this may not be possible in this species. In this case, I agree with the reviewer that a good way forward would be to provide both, the original and the filtered versions, and discuss the uncertainties around the gene models in the paper.

We now make it clearer that transcriptomes of closely related squid species were used to guide the annotation process (since it is impossible to get that type of data from a giant squid). We also provide the two sets of annotations and extended our discussion regarding the gene models in the main text (added information from Lines 252 to 278).

####################

Reviewer reports:
Reviewer #2: The authors have addressed most of my comments. However, I am still cautious about their gene model prediction. Running gene prediction using parameters from other species, especially Drosophila usually gives rise to very inaccurate results. The best situation would be using the transcriptome from the same species to train the gene model predictor. I understand there might be a technical limitation, but applying a random filter threshold to reduce the numbers of gene models is also problematic. This filtering may remove lineage-specific genes (i.e., novel genes in this species) and neural peptide genes that are usually very short. If having a good gene model is not possible, I would recommend the authors providing both versions of their gene models (i.e., original and filtered). And the authors should address this weakness in their manuscript.

Please note that the model parameters that were used for the final gene prediction were A. dux specific, they were definitely not D. melanogaster parameters. D. melanogaster parameters were used only as a starting point in the iterative process that has been guided, among other things, by RNA-seqs and proteomes from closely-related oegopsid squid species (unfortunately, we cannot obtain RNA-seq from A. dux due to difficulties of obtaining RNA from long-dead specimens). The RNA-seq and proteome information has also been used in the final stage of gene predictions. In this setup, the gene finder can adapt to new species (even species distant from the original parameters) and can give predictions that is in high concordance with related RNA-seq / proteome information where such information is available, while still predicting novel genes in the areas not covered by such evidence.

Methodology of iterative adaptation of gene finding parameters to new species has been previously rigorously evaluated by us (see reference [32] in the paper) as well as others (see e.g. Korf 2004, Lomsadze et al. 2005) and has been confirmed to lead to fast adaptation of the parameters to new species. We have made additional changes to the text describing gene finding to make this more apparent.

As to the high number of gene predictions, we think that this is mostly artefact of low contiguity of the assembly (lots of sequencing gaps) that leads to shorter gene models. (This issue is already discussed in the paper.) You are, of course, correct in pointing out that filtering for “supported” genes may lead to exclusion of truly novel genes. Based on your suggestion, we now provide both original and filtered data sets of gene models.

We base the downstream functional analysis on the filtered gene set, which is done based on sequence similarity to transcriptomes and proteomes of related species (not based on a length cutoff). Note that we are unable to assign putative functional characterization to genes without any additional evidence, since such assignment is done based mostly on sequence similarity. Thus, genes that were filtered out are unlikely to affect downstream analysis in significant ways, yet we agree that they may be a useful resource for other subsequent studies.

Please note the added information within the text extending from line 252 to line 278, which includes the extra references (below for details).

Korf I. Gene finding in novel genomes. BMC bioinformatics. 2004 Dec;5(1):59.

Lomsadze A, Ter-Hovhannisyan V, Chernoff YO, Borodovsky M. Gene identification in novel eukaryotic genomes by self-training algorithm, Nucleic Acids Res. , 2005, vol. 33 (pg. 6494-6496)

Minor comments:

Lines 261-262: "Drosophila melanogaster" -> use italic type
Done.

Line 265: "A. dux" -> use italic type
Done.

Line 266: "A. dux" -> use italic type
Done.

Close
