# Supplementary material for: A draft genome sequence of the elusive giant squid, Architeuthis dux
Source: Gigascience. 2020 Jan 16;9(1):giz152. doi: 10.1093/gigascience/giz152 (PMC6962438; doi:10.1093/gigascience/giz152)
Supplement: giz152_Reviewer_1_Report_Original_Submission — Kevin Kocot -- 7/25/2019 Reviewed [file giz152_reviewer_1_report_original_submission.pdf]

Reviewer Report

Title: A draft genome sequence of the elusive giant squid, Architeuthis dux

Version: Original Submission **Date:** 7/25/2019

Reviewer name: Kevin Kocot

Reviewer Comments to Author:

In this study, de Fonseca et al. report the genome of the giant squid as a resource to investigate the unique traits of this fascinating organism. Two assemblies, which are of comparable contiguity to most other recently published molluscan genomes, as well as a set of over 51,000 gene models are reported. Analysis of the genome focuses on repetitive elements (e.g., TEs), non-coding RNAs, and gene families of interest to the authors (WNT genes, Protocadherins, Hox genes, and reflectins). Overall this is a straightforward study that provides a resource that will be broadly useful and I feel it should be published. However, I have a number of suggestions for improvement including a few important issues that need to be addressed.

Major points:

It is unclear why two different genome assemblies are presented instead of just one most optimal assembly. This is not the way I would have gone about assembling this combination of data but presumably Dovetail scaffolding and gene modelling were performed before PacBio sequencing and scaffolding? Re-doing the assembly would a more logical way would probably have relatively little improvement but a little more explanation of the rationale or 'historical' reasons for two different assemblies and/or this assembly strategy would be a helpful addition to readers looking in the literature for examples on best practices for genome assembly.

Related to this issue, there is little comparison of the two genome assemblies and it is unclear which assembly was used for what analyses and even Table 1 and Table S2's titles are a bit ambiguous with respect to which assembly statistics are presented. Please explicitly state which assembly was used for which analyses.

The approach used for gene annotation is unconventional and the inferred number of protein-coding gene models is very high. This does not mean the gene model set is bad, but I feel that data needed for the reader to assess the quality of the gene models are lacking. Please run BUSCO on the gene models and report these data as well.

Specimen collection data are not reported in the manuscript.

Minor points:

Scientific names of species need to be italicized throughout.

Did all the giant squid DNA come from the same individual?

Lines 140-141: "currently increasing locally" is a bit awkward and vague.

Line 176: Which reads? All Illumina reads? PE reads only?

Line 185: Again, this seems to me to be a strange assembly strategy and I think that it should be clearly stated that PacBio data became available 'late in the game' if that is the case. Otherwise, the logic behind this assembly strategy needs to be explained.

Line 199: High-throughput is misspelled.
Line 203: Clarify what is meant by reference transcriptome. All reads from all tissues were pooled and assembled together?
Line 205: "EvidencialGene" is a tyo.
Lines 261-262: Please provide details on exactly what was done in this study in the supplementary material. Description of how the final gene models were selected is vague.
Line 277: What is meant by a "bespoke pipeline"? Custom scripts should be made available.
Line 450: Correct "Sampling was following"
BUSCO results are presented in the methods section (should be in the results by the way) for the pre-PacBio scaffolding genome but not the post-PacBio scaffolding genome.
Table 1: BUSCO should be in all capital letters.
Figure 3: What does the note "Gene size only" mean?
Table S1: Please provide total number of reads and somewhere it should be clarified how many different instrument runs were conducted and if different libraries were multiplexed on the Illumina platform.

Level of Interest

Please indicate how interesting you found the manuscript: Choose an item.

Quality of Written English

Please indicate the quality of language in the manuscript: Choose an item.

Declaration of Competing Interests

Please complete a declaration of competing interests, considering the following questions:

- Have you in the past five years received reimbursements, fees, funding, or salary from an organisation that may in any way gain or lose financially from the publication of this manuscript, either now or in the future?
- Do you hold any stocks or shares in an organisation that may in any way gain or lose financially from the publication of this manuscript, either now or in the future?
- Do you hold or are you currently applying for any patents relating to the content of the manuscript?
- Have you received reimbursements, fees, funding, or salary from an organization that holds or has applied for patents relating to the content of the manuscript?
- Do you have any other financial competing interests?
- Do you have any non-financial competing interests in relation to this paper?

If you can answer no to all of the above, write 'I declare that I have no competing interests' below. If your reply is yes to any, please give details below.

I declare that I have no competing interests

I agree to the open peer review policy of the journal. I understand that my name will be included on my report to the authors and, if the manuscript is accepted for publication, my named report including any attachments I upload will be posted on the website along with the authors' responses. I agree for my report to be made available under an Open Access Creative Commons CC-BY license (http://creativecommons.org/licenses/by/4.0/). I understand that any comments which I do not wish to be included in my named report can be included as confidential comments to the editors, which will not be published.

Choose an item.

To further support our reviewers, we have joined with Publons, where you can gain additional credit to further highlight your hard work (see: https://publons.com/journal/530/gigascience). On publication of this paper, your review will be automatically added to Publons, you can then choose whether or not to claim your Publons credit. I understand this statement.

Yes Choose an item.
