# Supplementary material for: A draft genome sequence of the elusive giant squid, Architeuthis dux
Source: Gigascience. 2020 Jan 16;9(1):giz152. doi: 10.1093/gigascience/giz152 (PMC6962438; doi:10.1093/gigascience/giz152)
Supplement: giz152_Reviewer_2_Report_Original_Submission — Yi-Jyun Luo -- 7/25/2019 Reviewed [file giz152_reviewer_2_report_original_submission.pdf]

Reviewer Report

Title: A draft genome sequence of the elusive giant squid, Architeuthis dux

Version: Original Submission **Date:** 7/25/2019

Reviewer name: Yi-Jyun Luo

Reviewer Comments to Author:

The authors present the genome of the giant squid Architeuthis dux. Several cephalopod genomes have been sequenced, but our genomic understanding of cephalopods living in the deep-sea environment is still poor. The authors sequenced a giant squid species A. dux together with several transcriptomes from the gonad, liver and brain tissues derived from three other squid species including Onychoteuthis banksii, Dosidicus gigas, and Sthenoteuthis oualaniensis.
Having a giant squid genome is an important contribution to the field of cephalopod genomics, especially for further meaningful comparative genomics. The authors provide a decent genome assembly. And the observation of a non-tightly physically linked Hox cluster is interesting. The manuscript is well written in general, however, there are a lot of editing errors throughout the whole manuscript, which distracts the reading. The authors need to carefully fix all these typos and errors during the revision. Further comments are provided below.
Major comments:
1. In the Abstract/Findings, there is a lot of information about "Methods" (e.g. how many raw reads, sequencing of proteome and RNA) instead of what the authors found from the genome itself. Also, the statement "RNA from three different tissue types from three other species of squid to assist genome annotation." is very vague. What tissue types from what species should be clearly described. The authors need to rewrite this section.
2. Line 153: Body patterning system? Usage of body patterning is confusing here since body patterning often refers to the developmental process during embryogenesis but not the skin color pattern.
3. The authors cited that there is a global proliferation of cephalopods (Lines 140 and 141) but later cited other studies saying that there is a regional extinction. It is a bit confusing whether cephalopods are undergoing proliferation or extinction. Given that the earlier citation is more recent (Doubleday et al., 2016) than others, it is wondering which condition is closer to the current situation.
4. Although it is agreeable in general to have genome resources from unexplored species, the authors' argument in the last paragraph of Data description/Context is not convincing. The link between having a genome and aiding conservation efforts as well as ensuring continued existence is not clear.
5. Do the authors have any idea why the genome contains so many protein-coding genes (51,225 genes predicted) in comparing to other cephalopod species usually having only 20,000-30,000 genes? For example, is it due to that A. dux has more lineage-specific genes or expansions of certain gene families?
6. Given that genome size and polyploidy of the organisms are often correlated to increased body size (Session et al., 2016), have the authors checked if there is whole-genome duplication or polyploidy in

the A. dux genome?
Session et al. (2016) Genome evolution in the allotetraploid frog Xenopus laevis. Nature 538, 336-343.
7. Figure 3: The authors should provide scaffold numbers for the Hox clusters from each species. Also, in most cases, Hox genes in the Hox cluster are adjacent to each other without the insertion of other non-Hox genes. If there is a special case in A. dux and E. scolopes, the authors should show the real gene arrangement on that scaffold, especially for the non-Hox genes (with brief annotation) that are in between Hox genes. This can be achieved by having an additional panel in the same figure. The authors are encouraged to show an illustration on the types of Hox gene organization in order to give the readers a better understanding of this context.
Minor comments:
1. Line 149: ~2cm -> "~2 cm"
2. Line 150: 3 orders -> "three orders"
3. Line 150: Architeuthis dux -> "A. dux"
4. Lines 150 and 151: 10-12cm… 20m -> "10-12 cm… 20 m"
5. Line 152: 500kg -> "500 kg"
6. Line 171: a Architeuthis dux sample -> "an A. dux sample"
7. Line 172: What is CTAB?
8. Line 184: For Eukaryota and Metazoa we identified… -> "For Eukaryota and Metazoa, we identified…"
9. Line 184: … 90.4 % and 92.1 %... -> "… 90.4% and 92.1%..."
10. Line 185: 23.38Gb -> "23.38 Gb"
11. Line 186: 14.79kb -> "14.79 kb"
12. "k-mer" (Line 204) or "kmer" (Line 176) to be consistent.
13. Line 216: 100,000 g -> "100,000Ã—g"
14. Lines 219 and 222: SDS-PAGE -> "SDS-PAGE" (hyphen but not en dash)
15. Line 221: Tris - HCl -> Tris-HCl (single hyphen but not en dash with spaces)
16. Line 226: LC-MS/MS analyses -> "LC-MS/MS analyses" (hyphen but not en dash)
17. Line 254: Using italic for scientific names (i.e. Octopus bimaculatus, Crassostrea gigas, and Lottia gigantea)
18. Line 260: … 200kb (total length 199Mb)… -> "… 200 kb (total length 199 Mb)…"
19. Line 290: Transposable elements -> "transposable elements"
20. Line 300: Architeuthis dux -> "A. dux"
21. Line 323: ~5-8% -> "~5Â¬-8%" (en dash but not hyphen for a range)
22. Line 381: Octopus bimaculoides -> "O. bimaculoides"
23. Line 383: Euprymna scolopes -> "E. scolopes" (in italic)
24. Line 395: Euprymna scolopes -> "E. scolopes"
25. Lines 397 & 398: 500 - 10,000 kb -> "500-10,000 kb" (en dash but not hyphen for a range)
26. Line 406: ... observed in Hox 1, Hox 4, ANTP, Lox 2, Lox 5, Post 1 and Post 2. Hox 1 did,... -> "... observed in Hox1, Hox4, ANTP, Lox2, Lox5, Post1 and Post2. Hox1 did,..."
27. Line 407: Hox 1 -> "Hox1"
28. Line 408: Hox 3 -> "Hox3"
29. Line 409: Lox 4 -> "Lox4"

30. Lines 410, 412 & 413: Hox 2 -> "Hox2"
31. Line 421: ... contains 7 reflectin genes and 3 reflectin-like genes… -> "... contains seven reflectin genes and three reflectin-like genes…"
32. Line 422: … exception of 1 reflectin gene, … -> "… exception of one reflectin gene, …"
33. Line 436: … (tsa)… -> "… (TSA)…"
34. Lines 647 & 657: Architeuthis dux -> "A. dux"
35. Line 659: Hox 2 -> "Hox2"

Level of Interest

Please indicate how interesting you found the manuscript: Choose an item.

Quality of Written English

Please indicate the quality of language in the manuscript: Choose an item.

Declaration of Competing Interests

Please complete a declaration of competing interests, considering the following questions:

- Have you in the past five years received reimbursements, fees, funding, or salary from an organisation that may in any way gain or lose financially from the publication of this manuscript, either now or in the future?
- Do you hold any stocks or shares in an organisation that may in any way gain or lose financially from the publication of this manuscript, either now or in the future?
- Do you hold or are you currently applying for any patents relating to the content of the manuscript?
- Have you received reimbursements, fees, funding, or salary from an organization that holds or has applied for patents relating to the content of the manuscript?
- Do you have any other financial competing interests?
- Do you have any non-financial competing interests in relation to this paper?

If you can answer no to all of the above, write 'I declare that I have no competing interests' below. If your reply is yes to any, please give details below.

I declare that I have no competing interests.

I agree to the open peer review policy of the journal. I understand that my name will be included on my report to the authors and, if the manuscript is accepted for publication, my named report including any attachments I upload will be posted on the website along with the authors' responses. I agree for my report to be made available under an Open Access Creative Commons CC-BY license (http://creativecommons.org/licenses/by/4.0/). I understand that any comments which I do not wish to

be included in my named report can be included as confidential comments to the editors, which will not be published.

Choose an item.

To further support our reviewers, we have joined with Publons, where you can gain additional credit to further highlight your hard work (see: https://publons.com/journal/530/gigascience). On publication of this paper, your review will be automatically added to Publons, you can then choose whether or not to claim your Publons credit. I understand this statement.

Yes Choose an item.
